# PUMA: A Unified Framework for Penalized Multiple Regression Analysis of GWAS Data

**DOI:** 10.1371/journal.pcbi.1003101

**Published:** 2013-06-27

**Authors:** Gabriel E. Hoffman, Benjamin A. Logsdon, Jason G. Mezey

**Affiliations:** 1 Department of Biological Statistics and Computational Biology, Cornell University, Ithaca, New York, United States of America; 2 Department of Genome Sciences, University of Washington, Seattle, Washington, United States of America; 3 Department of Genetic Medicine, Weill Cornell Medical College, New York, New York, United States of America; UC Berkeley, United States of America

## Abstract

Penalized Multiple Regression (PMR) can be used to discover novel disease associations in GWAS datasets. In practice, proposed PMR methods have not been able to identify well-supported associations in GWAS that are undetectable by standard association tests and thus these methods are not widely applied. Here, we present a combined algorithmic and heuristic framework for PUMA (Penalized Unified Multiple-locus Association) analysis that solves the problems of previously proposed methods including computational speed, poor performance on genome-scale simulated data, and identification of too many associations for real data to be biologically plausible. The framework includes a new minorize-maximization (MM) algorithm for generalized linear models (GLM) combined with heuristic model selection and testing methods for identification of robust associations. The PUMA framework implements the penalized maximum likelihood penalties previously proposed for GWAS analysis (i.e. Lasso, Adaptive Lasso, NEG, MCP), as well as a penalty that has not been previously applied to GWAS (i.e. LOG). Using simulations that closely mirror real GWAS data, we show that our framework has high performance and reliably increases power to detect weak associations, while existing PMR methods can perform worse than single marker testing in overall performance. To demonstrate the empirical value of PUMA, we analyzed GWAS data for type 1 diabetes, Crohns's disease, and rheumatoid arthritis, three autoimmune diseases from the original Wellcome Trust Case Control Consortium. Our analysis replicates known associations for these diseases and we discover novel etiologically relevant susceptibility loci that are invisible to standard single marker tests, including six novel associations implicating genes involved in pancreatic function, insulin pathways and immune-cell function in type 1 diabetes; three novel associations implicating genes in pro- and anti-inflammatory pathways in Crohn's disease; and one novel association implicating a gene involved in apoptosis pathways in rheumatoid arthritis. We provide software for applying our PUMA analysis framework.

## Author Summary

Genome-wide association studies (GWAS) have identified hundreds of regions of the human genome that are associated with susceptibility to common diseases. Yet many lines of evidence indicate that many susceptibility loci, which cannot be detected by standard statistical methods, remain to be discovered. We have developed PUMA, a framework for applying a family of penalized regression methods that simultaneously consider multiple susceptibility loci in the same statistical model. We demonstrate through simulations that our framework has increased power to detect weak associations compared to both standard GWAS analysis methods and previous applications of penalized methods. We applied PUMA to identify novel susceptibility loci for type 1 diabetes, Crohn's disease and rheumatoid arthritis, where the novel disease loci we identified have been previously associated with similar diseases or are known to function in relevant biological pathways.

## Introduction

Genome-wide association studies (GWAS) have identified many susceptibility loci underlying the molecular etiology of complex diseases [1]. These studies have been responsible for the discovery of many individual genes that contribute to disease risk [2]–[10], for discoveries on the front line of personalized medicine [11], [12], and for discovering novel pathways important for the progression of complex heritable diseases [13]. The expense of each GWAS that is capable of finding well-supported disease loci is considerable and, as a consequence, each robust and interpretable association discovered in a GWAS is valuable, not only from the point of view of scientific discovery but also in terms of return on investment [14], [15]. A clear picture that has an important bearing on the investment-discovery tradeoff in GWAS experiments is that the associations identified to date generally explain only a small to moderate fraction of total heritability [16], [17]. Recent analyses have suggested that a considerable amount of this ‘missing’ heritability can be accounted for by rare variants or variants with weak effects [18]–[20]. This suggests that there is an opportunity to identify more risk loci through studies that require even greater investment, by including larger sample sizes and/or by incorporating higher genetic marker coverage of the genome by using next-generation sequencing (NGS). The novel associations discovered by large consortia GWAS studies support this supposition [7]–[10]. Another complementary strategy that leverages both the current and future investment in GWAS experiments is the application of new statistical analyses that can reliably identify weaker associations [21]–[25]. Although there has been an explosion of methods in this area [26], [27], few have produced robustly supported associations that are not detectable by single marker tests of association [1], [26]–[29].

Here, we report a general framework for applying a family of GWAS analysis methods that is extremely promising for detection of weak associations yet has not been widely applied to learn novel biology from GWAS datasets: penalized multiple regression (PMR) methods. PMR methods work by simultaneously incorporating tens to hundreds of thousands of genetic markers in a single statistical model where a penalty is incorporated to force most marker regression coefficients to be exactly zero, so that only a small fraction are estimated to make a contribution to disease risk [22], [30]–[39]. By jointly analyzing markers, PMR methods are able to consider the correlation of each marker with the phenotype, conditional on all other relevant markers. This can increase the power to detect weak associations compared to single marker methods due to the smaller residual variance and the fact that the conditional correlation of a marker with the phenotype can often be substantially higher than the marginal correlation [40]. The latter effect is a consequence of non-zero correlation structure between associated markers when the underlying genetic architecture is polygenic [41]. These methods therefore model the underlying biology more accurately than single marker tests, by explicitly modeling the polygenic architecture of complex phenotypes to account for the effects of multiple susceptibility loci. They also leverage the same type of statistical model used in single marker testing methods that have demonstrated reliability in the identification of strong associations [1], [28], [29]. Yet, despite theoretical power of PMR methods, the large body of statistical literature exploring their theoretical properties (see reviews [42], [43]), and the recent interest in the methods development community [22], [30]–[39], these methods have not been successful in GWAS analysis. This is due to a combination of limitations: 1) inability to scale for very large GWAS datasets [22], [32], [34], [35], 2) poor performance on simulated data [22], [31], 3) they often find too many ‘hits’ to be biologically plausible for a given GWAS sample size [22], and 4) they do not identify novel, well-supported associations that are not detectable by standard methods [22], [31].

**Figure 1:**
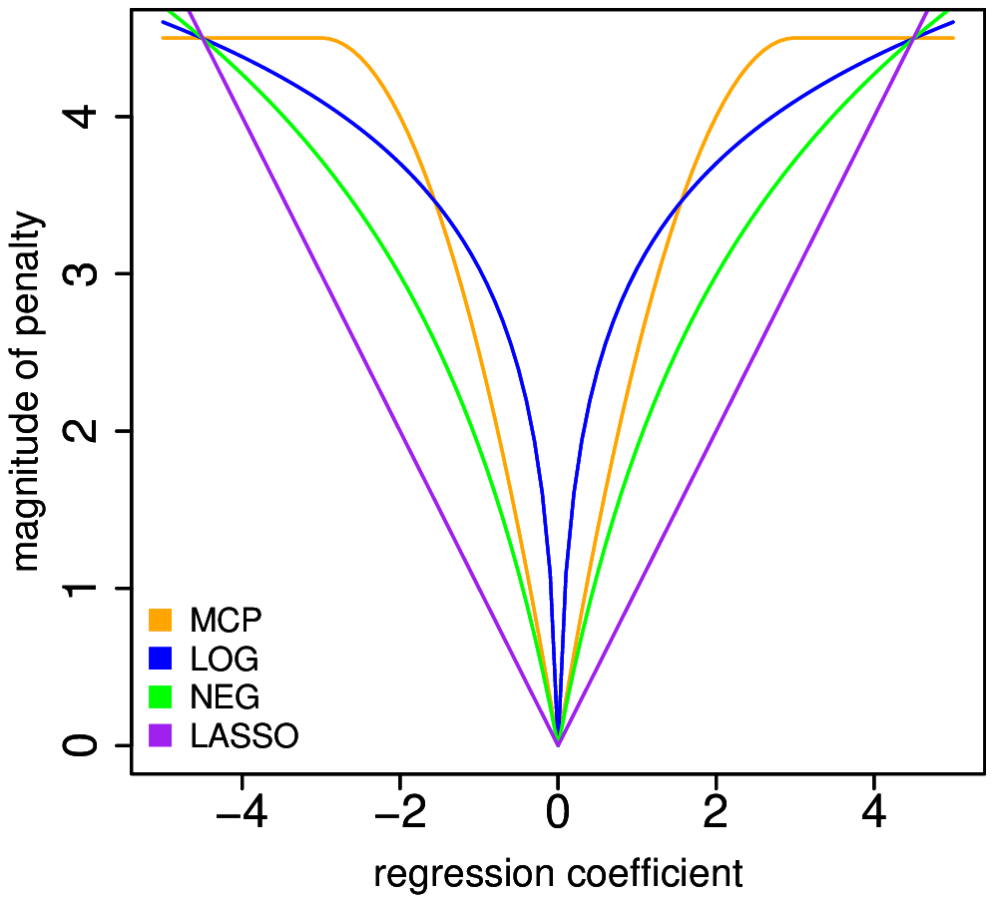
Penalty functions on the magnitude of the regression coefficients implemented in the PUMA framework. A parameter determines the slope near the origin for all penalties, while MCP, LOG and NEG have an additional tuning parameter determining the rate at which the derivative of the penalty tails off to zero.

In order to address these issues, we present a combined algorithmic and heuristic framework for PUMA (Penalized Unified Multiple-locus Association) analysis that optimizes these methods for reliable detection of weak associations when applied to large GWAS datasets. The complete PUMA framework includes an extremely efficient implementation of a new minorize-maximization (MM) algorithm [44] for generalized linear models (GLM) [45], a theoretically motivated data-adaptive heuristic approach to determine penalty strength and for model selection, and *post hoc* methods for assessing the rank of identified associations. Within PUMA, we implement all sparse feature selection, penalized regression approaches proposed for GWAS analysis to date, including four penalties implemented in a maximum likelihood framework (i.e. Lasso, Adaptive Lasso, NEG, MCP), as well as theoretically justified penalties that have not been previously applied to GWAS (i.e. LOG) (Figure 1). We demonstrate the power of our framework for detecting weaker associations that are invisible to individual marker testing through analysis of simulated GWAS data that mirror observations from analyses of real GWAS data. We also demonstrate that our approaches correct issues with all current PMR methods where software is available for GWAS analysis, where we find that all of these currently available PMR GWAS methods can perform worse than single marker testing for our simulation conditions. As an illustration of the value of PUMA for mining existing GWAS data for novel associations, we apply these methods to the original Wellcome Trust Case Control Consortium (WTCCC) [2] GWAS datasets for type 1 diabetes, Crohn's disease and rheumatoid arthritis. Our re-analysis identifies weak associations that implicate additional susceptibility loci for these autoimmune diseases, which did not appear significant by standard single marker tests of association in these datasets, yet were 1) identified in an independent GWAS of the same phenotype that did not include WTCCC data, 2) previously known to play a role in disease etiology, or 3) known to function in a relevant biological pathway. Our results demonstrate that appropriately tuned PMR methods can provide a complementary approach to large meta-analyses [4]–[10] to identify susceptibility loci with weak associations. We also provide a discussion concerning how the framework can be extended to perform penalized analysis of epistasis, to incorporate mixed model analysis, and to address challenges of genome-wide genotypes provided by whole-genome next-generation sequencing.

## Results

### PUMA is a scalable framework for GWAS analysis

The methods implemented in our PUMA framework are orders of magnitude faster than existing software when assigned identical computational tasks and no pre-screening of markers is performed (Table 1). This substantial boost in computational speed allows PUMA to perform a dense two-dimensional search of tuning parameter values for non-convex penalties (i.e. MCP, NEG, LOG) and examine upwards of 1 million total modes of the likelihood surface for simulated case/control dataset of 5,000 individuals and 650K genetic markers in less than 24 hours on a 6 core Intel® Xeon® W3690 @ 3.47 GHz with 12 Gb memory when a pre-screening p-value cutoff of 0.01 from single marker analysis is applied (Table 2). This is a huge improvement compared to existing software for non-convex PMR methods [22], [32] which only examine a single mode.

**Table 1:** Run times for PUMA and other available software for identical analyses.

	Sample size		
Method	1000	2000	5000
Lasso	2 m 11 s	5 m 55 s	14 m 45 s
NEG	1.2 s	2.2 s	9.8 s
MCP	4.7 s	8.2 s	29.2 s
Mendel [31] (Lasso)	9 m 50 s	NA	NA
HyperLasso [22] (NEG)	52 m 24 s	4 h 16 m	20 h 3 m
grpreg [32] (MCP)	3 h 52 m	NA	NA

For a typical simulated data set with 650 K markers, no pre-screening of markers and sample sizes, 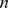, of 1000, 2000 and 5000, we report run times for available software and PUMA performing the same analyses. For Lasso, we had Mendel and PUMA perform a search of tuning parameter space in order to return 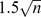 markers with nonzero coefficients. For NEG, we set HyperLasso to its default tuning parameter values and ran PUMA with the same values. For MCP, we set grpreg and PUMA to perform a search of tuning parameter space in order to return 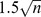 markers with nonzero coefficients, where 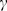 was set to 30 as per Ayers and Cordell [32]. Analysis was performed on an 8 core Intel® Xeon® E5520 @ 2.27 GHz with 32 Gb memory. NA indicates the program crashed due to insufficient memory; we note that this is due to technical limitations of Mendel and R, in which grpreg runs.

**Table 2:** Run times for PUMA methods and other available software.

Method	Run time	# of models	# of unique models
Lasso	33 s	156	59
Adaptive Lasso	5 s	21	13
LOG	6 hrs	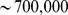	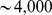
NEG	5 hrs	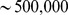	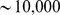
1D-MCP	21 min	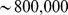	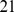
2D-MCP	14 hrs	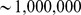	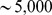
Mendel [31]	66 s	1	1
HyperLasso [22]	1 hr	1	1
perm-MCP [32]	1 hr	1	1

For a typical simulated data set with 5000 individuals, 650 K markers and a pre-screening p-value threshold of 0.01, we report the run times, and the number of total and unique models examined by our methods (top) and available methods using standard/default settings (bottom). We list the number of models assessed during a single run of a method where a model is defined by the set of markers with distinct nonzero coefficients and the number of unique models counts the number of sets of distinct markers, where we note that the metrics reported can vary substantially between datasets. Lasso and Adaptive Lasso are convex and have a single tuning parameter, so relatively few models are examined during the search. For convex penalties, each distinct tuning parameter value produces a model, although another tuning parameter value can cause the coefficients to change but still produce the same set of markers with nonzero coefficients. Thus the number of models examined is larger than the number of unique models. MCP, LOG and NEG penalties are non-convex and have two tuning parameters and were applied with 100 marker reorderings, so they produce orders of magnitude more total and unique models. We note that 1D-MCP is faster than 2D-MCP as the former fixes the value of one tuning parameter. We note that HyperLasso [22] can be extremely computationally expensive for large datasets, so that the time we report is based on analysis of the pre-screened dataset where pre-screening step must be implemented separately. Ayers and Cordell [32] do not provide software but proposes an approach using the *grpreg* package in R.

### Theoretical and empirical justification for pre-screening markers

While pre-screening markers based on a p-value cutoff may initially seem to detract from the purpose of a multiple-locus analysis, it is supported by statistical theory, is necessary for large scale analysis and has almost no impact on the set of markers identified as associated. In a seminal paper, Fan and Lv [46] demonstrate that pre-screening by ranking the marginal correlation of each variable with the response will retain the relevant variable asymptotically with probability tending to 1. Fan and Song [43] extend this result to generalized linear models. Moreover, Tibshirani, et al. [47] and El Ghaoui, et al. [48] establish exact pre-screening methods for linear and logistic Lasso models where relevant variables are guaranteed to be retained for finite sample sizes and demonstrate that the number of variables can be reduced by up to 3 orders of magnitude. Intuitively, both the asymptotic [43], [46] and exact pre-screening methods [47], [48] rely on the fact that a variable is unlikely to have a very small marginal correlation with the response but a large and very significant conditional correlation for a particular sample size when the relevant variables explain only a small fraction of the variation in the response. Moreover, pre-screening is often computationally necessary because storing 650 K markers for 5000 samples requires 26 Gb of memory. Finally, we note that pre-screening is used by previous applications of PMR methods to GWAS data [22], [31] in order to handle genome-scale data.

We use a pre-screening p-value cutoff based on single marker analysis, because 1) it retains all relevant variables asymptotically [43], [46], 2) it approximates the exact methods proposed for Lasso [47], [48], which cannot be easily adapted to other penalties, 3) it reduces memory requirements so that very large datasets can be analyzed on a high-end desktop computer, 4) it substantially reduces the computational burden, 5) by using a p-value it is naturally calibrated to the sample size and the fraction of variation in the response being explained, and 6) it has very little empirical effect on the results.

We demonstrate this final and most important point in two complementary simulation studies. First we consider a simple two-step forward regression method, which is known to approximate penalized multiple regression [49], [50] and, under a range of biologically motivated simulation conditions, demonstrate that variables that do not cross an initial p-value threshold have a very low probability of being significant in the second step (Figure S1). Second we demonstrate that the pre-screening has no noticeable effect on the performance of Lasso and MCP methods but substantially reduces the computational time (Figure S2).

### Simulated data assessment of the PUMA framework

We analyzed 960 simulated GWAS datasets to assess the performance of our PUMA framework compared to other published methods for PMR GWAS analysis. We note that these simulations, while far more extensive than other published works on PMR GWAS analysis [22], [30]–[38] are not meant to be exhaustive or to capture all the possible complexities in a GWAS but rather to: 1) serve as a baseline for comparing GWAS analysis methods and 2) provide an estimate of the expected performance for these methods when applied to GWAS data under relatively ideal experimental conditions. Our goal therefore was not to attempt to model a broad spectrum of possible GWAS data complexities (e.g. stratified experimental sampling schemes, known or cryptic population structure effects on phenotype, relatedness among individuals, measured or latent covariates, etc.) but rather to simulate data that captured the most basic components of a GWAS experiment (see Methods for details). In simulated data a causal variant is defined as a variant whose coefficient value is nonzero, so that number of minor alleles at this marker contributes to the phenotype. In order to mimic the fact that true causal variants are not available from array-based genotyping, the simulated causal variants were removed from the dataset so that they are not considered by the tests of association. Therefore, just like in all array-based genotyping datasets, our simulations identify associations based on markers in linkage-disequilibrium with the (omitted) causal variant.

### Assessment of available software for PMR GWAS

We assessed the performance of PMR methods for which there is available software. We compared the performance of the Lasso penalty from Wu, et al. [31], the NEG penalty as implemented in the HyperLasso program [22], and a permutation-based approach to selecting tuning parameter values for the MCP penalty [32], [51] that we term perm-MCP. We note that we only considered PMR approaches that are designed to handle the specific challenges of GWAS data and that also perform feature selection, such that we do not consider ridge, elastic net, or group-penalties since they set many correlated markers to have nonzero coefficients and thus complicate the generation of interpretable p-values [30], [52]. We also did not consider Markov Chain Monte Carlo (MCMC) approaches [34], [35] since they could not efficiently scale to genome-wide data while exploring a range of tuning parameter values. We ran the HyperLasso program [22] with standard settings (see Text S1). We applied the method of Wu, et al. [31], setting the number of selected markers to the true number of causal markers in each simulation since Wu, et al. [31] do not specify a criterion for selecting the model size. As a benchmark, we also ran a single marker analysis implemented by applying a logistic regression model to each marker individually. We used a pre-screening p-value cutoff of 0.01 from single marker analysis for the PMR methods to make them computationally tractable.

**Figure 2:**
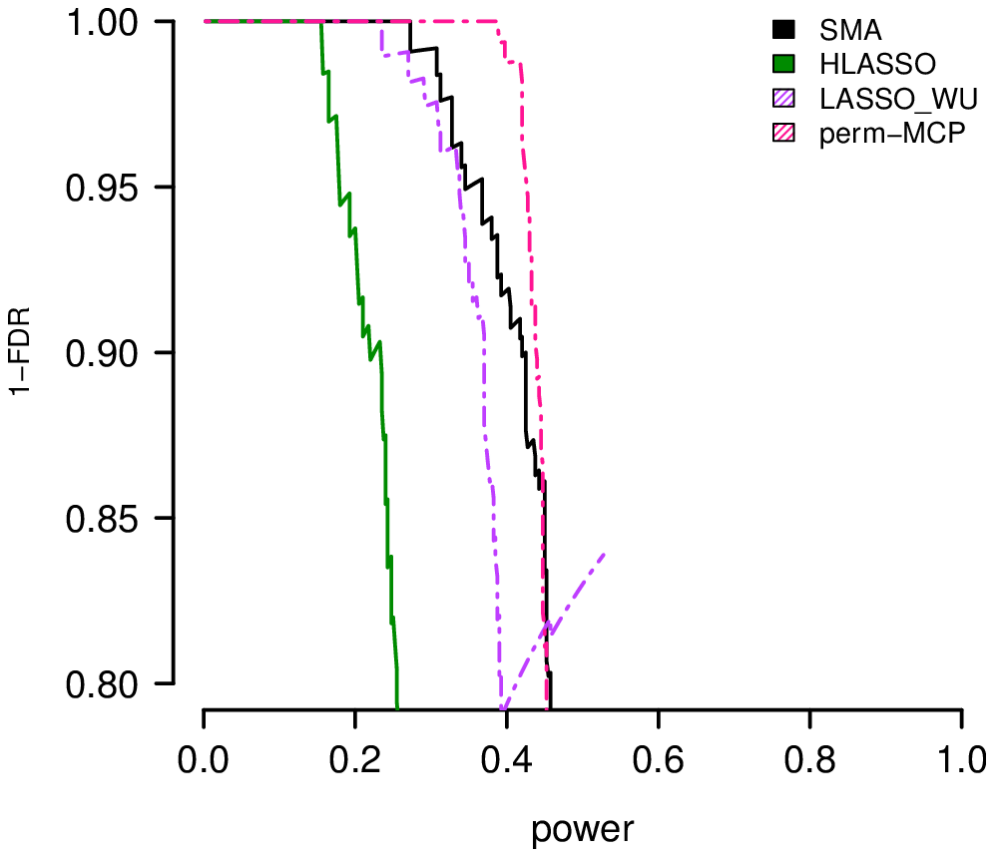
Simulation results for existing methods. Shown here are representative examples of simulation results for available software including the HyperLasso program [22] (HyperLasso), Lasso using the method of Wu, et al. [31] (LASSO_WU) and perm-MCP [32]. Power is compared to a standard single marker analysis (SMA). Results are shown for 20 replicate datasets from simulations with 5000 individuals, 20 causal markers affecting disease risk and a heritability of 50%. Note that perm-MCP selected very few markers per simulation so the false discovery rate did not exceed 10%.

Simulations indicate that HyperLasso [22] and the Lasso of Wu, et al. [31] are generally less powerful than a standard single marker test (Figure 2, S3, S4, S5, S6, S7, S8, S9, S10). While Lasso is sometimes comparable or slightly more powerful than a single marker test for low FDR, the performance of the method benefits from the fact that the number of selected markers is set using information not available in real data. Setting the marker number to 10 (the default in the implementation of Wu, et al. [31]) or another arbitrary value results in poor performance and is not competitive with a single marker test (results not shown). The performance of HyperLasso is especially poor as is it suffers from the fact that the choice of tuning parameters has a huge effect on performance, but the method does not implement a search over tuning parameter values. Moreover, HyperLasso does not include a way to evaluate the significance of a selected marker, so we used their default approach of using coefficient values from selected markers to assess performance. Alternatively, perm-MCP was the most powerful in our simulations.

We note that for perm-MCP, by setting the expected false positive rate (eFPR) and using permutations to obtain the value of the tuning parameter based on this rate, perm-MCP generates a single model with relatively few nonzero coefficients while explicitly addressing the multiple testing problem. Yet in practice this result indicates that perm-MCP may assign p-values to only a handful of markers so that the method may not identify any novel associations for a particular dataset. Since the number of nonzero coefficients is directly related to the specified eFPR and the pre-screening cutoff, we examined multiple eFPR values (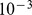, 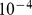, 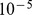, 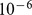, 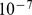) and cutoff values (0.1, 0.01, 0.001), and selected the values the yielded the highest power (eFPR = 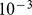, cutoff = 0.001) to present in Figure 2, where other cutoff combinations produce poorer performance (see Figure S11 for a representative plot showing the results for all cutoffs). We note that the eFPR value is based on the number of markers that pass the pre-screening cutoff, not the total number of markers. Therefore the performance of perm-MCP is sensitive to the eFPR and cutoff values, yet there is no clear method to optimally specify this value *a priori*. Furthermore, determining the appropriate cutoff for a desired eFPR for correlated high-dimensional data is the subject of current research [53], and its application to permutation methods for selecting a tuning parameter remains an open question. We also note that the performance achieved with PUMA methods does not require the optimal determination of eFPR and pre-screening cutoffs.

In addition, we note that while Ayers and Cordell [32] have previously shown that penalized regression methods can perform well on simulated data, the datasets we address here are orders of magnitude larger. Ayers and Cordell [32] conducted two simulation studies, one with 4000 markers and the other with no more than 228. By considering such a small set of markers, which is not the product of a pre-screening step, they were able to use standard R packages and apply a permutation method to select tuning parameters on the full dataset. Moreover, the multiple testing problem is less severe in their analysis. For the HyperLasso program, Ayers and Cordell [32] selected the tuning parameter as described by Hoggart, et al. [22]. However, using these settings for the genome-scale datasets examined here caused the HyperLasso program to crash (Text S1) and so we use the default program settings. We note that the program worked as expected for smaller datasets. It is unclear whether this problem is an issue with the underlying algorithm or the specifics of the implementation. Thus the difference between the performance of methods in Ayers and Cordell [32] and the current study is the scale of the data, the large multiple-testing burden for genome-scale data and the necessity of a pre-screening step for genome-scale data.

PUMA's statistical power is due to its data-adaptive properties. PUMA 1) performs a two dimensional search of the tuning parameter space 2) selects the number of nonzero coefficients based on both the fit to the data and the sample size, and 3) uses a heuristic methods to assess the significance of correlated markers. Conversely, perm-MCP fixes one of the tuning parameters, does not incorporate the sample size, and does not address the issues of testing the significance of correlated markers. Moreover, perm-MCP relies on setting the eFPR despite problem of determining an appropriate value *a priori* for high dimensional data.

### The potential of the PMR GWAS framework as implemented in PUMA

**Figure 3:**
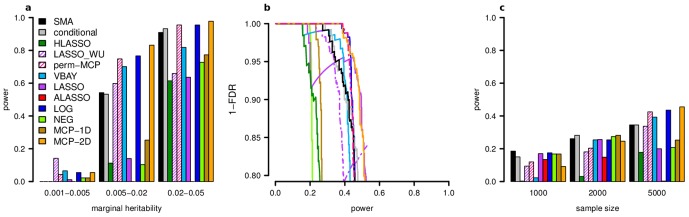
PUMA methods outperform other tests of association. Shown here are representative examples of simulation results for single marker analysis (SMA), 2-step conditional regression, a permutation based tuning of MCP (perm-MCP), our approximate Bayesian method (VBAY), and our PUMA methods (Lasso, Adaptive Lasso, LOG, NEG, 1D-MCP, 2D-MCP). Results are shown for 20 replicate datasets from simulations with 5000 individuals, 20 causal markers affecting disease risk and a heritability of 50%. **a**) The power of each method to recover true associations at a fixed FDR of 5% shown as a function of the marginal heritability of each causal marker. **b**) Precision-Recall curve for the same simulations as in (a). Note that perm-MCP selected very few markers per simulation so the FDR did not exceed 10%. **c**) Power to recover true associations at an FDR of 5% for a range of sample sizes.

For the 960 simulated GWAS datasets we analyzed, almost all PMR GWAS approaches implemented in PUMA except NEG and adaptive Lasso outperformed single marker analysis under simulation conditions with sufficient sample size (Figure 3, Figures S3, S4, S5, S6, S7, S8, S9, S10). Quite critically, the performance is far greater even when using a conservative control of FDR that is commonly employed in GWAS studies. Moreover, the improvement of the PMR methods in PUMA is most noticeable for causal variants with intermediate marginal heritability. Overall, these simulations demonstrate that the advantage of PMR methods over a single marker test increases with sample size, but decreases with the number of susceptibility loci (Figure S3, S4).

While the penalized methods implemented in our PUMA framework consistently had higher power than single marker analysis as a function of FDR under most simulation conditions, none of the penalties consistently stood out as the most powerful. However, our PUMA framework, which includes a fast novel algorithm for penalized maximum likelihood estimation in generalized linear models, data-adaptive tuning of tuning parameters, heuristics for model selection and novel method of assigning p-values (see Methods) increased the power of PMR methods compared to current approaches using the same penalties [22], [31]. We note that our implementation of the NEG penalty showed a substantial increase in power over the HyperLasso program [22] and indicates that our search over tuning parameter values and heuristic approach for model selection was successful. Moreover, our search of one or both tuning parameter values for MCP (termed 1D-MCP and 2D-MCP, respectively) showed that our approach to applying MCP (i.e. 2D-MCP) can be more powerful than that of Ayers and Cordell [32]. The fact that our implementation of Lasso had higher power than the version of Wu, et al. [31] confirms the usefulness of our data-adaptive approach for selecting penalty strength and our novel method for assigning p-values. We also note that for comparison we applied a conditional regression test and our previously published algorithm VBAY, a variational Bayes approach for fitting a mixture prior penalty [33]. We found that perm-MCP and VBAY had similar performance to our PMR methods and while the conditional test of association was sometimes more powerful than single marker analyses it was generally not as powerful as the PUMA PMR methods.

### Summary of Wellcome Trust Case Control Consortium (WTCCC) re-analysis

In our re-analysis of type 1 diabetes, Crohn's disease and rheumatoid arthritis datasets, we applied a single-marker analysis and all PMR analysis approaches (Lasso, Adaptive Lasso, NEG, LOG, 1D-MCP, 2D-MCP, perm-MCP) using all the recommended components of our framework. We included sex and the first two principal components as unpenalized covariates, applied a pre-screening cutoff of 0.01 on the p-values from the single marker test, and ran 100 reorderings for the non-convex penalties. Quantile-Quantile (QQ) plots of p-values from a standard single marker analysis indicate that the effects of any remaining population structure is minimal. Moreover, including the subset of significantly associated markers identified by the PMR methods as covariates in a single marker analysis of remaining markers does not yield an inflation of the QQ plots and thus indicates that the PMR methods are not overfitting the data (Figure S12). We also note that due to the complex LD around the MHC on chromosome 6, while we included this region in our analysis, we omit this region from any *post hoc* analysis and discussion.

**Figure 4:**
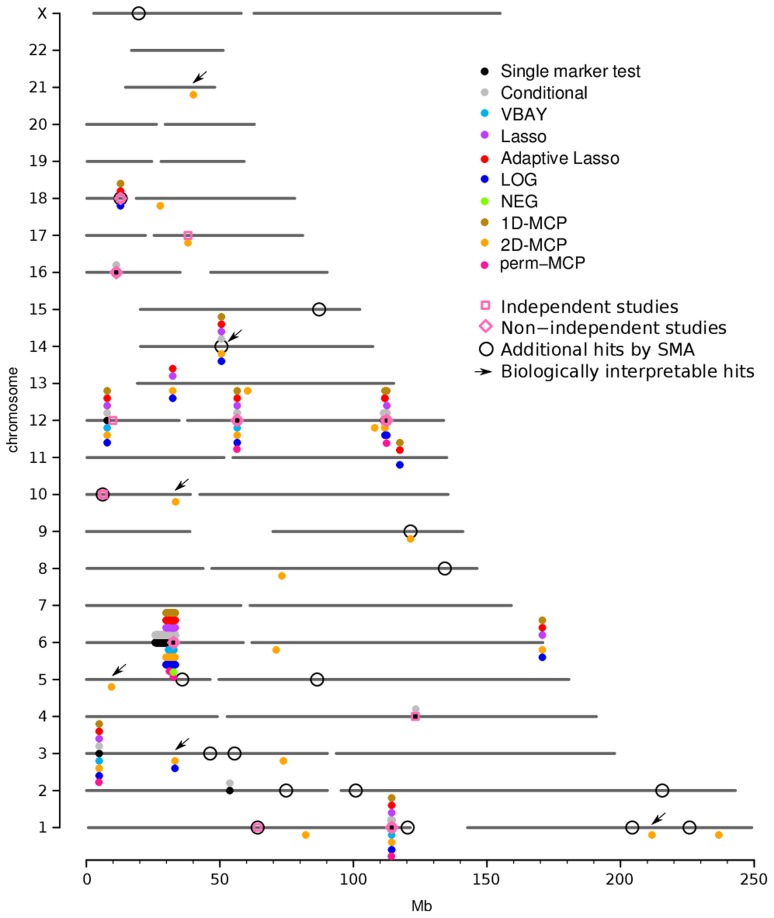
PUMA identifies associations for Wellcome Trust Case Control Consortium (WTCCC) data that are novel and that overlap hits from previous GWAS. Genome-wide plot of associations identified by analyzing the WTCCC data for type 1 diabetes using PUMA and single marker tests. Replications from independent (not including WTCCC data) and non-independent (including WTCCC data) GWAS of the same disease are indicated with pink boxes and diamonds, respectively. For comparison, markers identified using a single marker association analysis are presented in black circles, where we note that these same hits are all identified by PUMA methods. Also for comparison, we relaxed the Bonferroni threshold for single marker analysis (open circles) until the same number of associations as found by PUMA methods are reported, where we note that many of these additional hits tend not to overlap PUMA hits or previous GWAS hits. Arrows indicate novel associations that are biologically interpretable (see Table 6).

Our single-marker re-analysis of type 1 diabetes, Crohn's disease and rheumatoid arthritis datasets reproduced the same associations as reported in the original analysis (Figure S13). Our PMR methods recapitulated almost all of the associations identified by single marker analysis, although there were differences among the methods. The PUMA Lasso and Adaptive Lasso identified almost no additional associations compared to single marker tests, and while LOG, NEG and 1D-MCP identified more, almost all of the associations found by these five methods (Lasso, Adaptive Lasso, LOG, NEG, 1D-MCP) were identified by 2D-MCP (Figure 4, S14). We note that perm-MCP identified very few associations (12 overall, across the three diseases), all but one of which was identified by a single marker test, and all were identified by 2D-MCP. We therefore discuss the associations found by 2D-MCP, where we consider three categories of interest (Table 3): those concordant with single marker tests, those that recapitulate associations identified in external GWAS studies but not by single marker analysis of the WTCCC, and novel associations, of which many were deemed to be biologically interpretable in terms of the current knowledge of disease etiology. In the absence of functional validation, the presence of a feasible biological interpretation lends more credibility to these novel findings.

**Table 3:** Number of associations identified in the analysis of Wellcome Trust Case Control Consortium (WTCCC) data by disease and category.

	CD	RA	T1D
Concordant with SMA	8	1	4
Replications not significant by SMA			
Independent datasets	0	0	1
Non-independent datasets	5	0	1
Etiologically relevant associations	3	1	6
Other novel associations	12	11	11

Number of associations identified for Crohn's disease, rheumatoid arthritis and type 1 diabetes divided into 5 categories for the union of all associations identified by PUMA methods.

**Figure 5:**
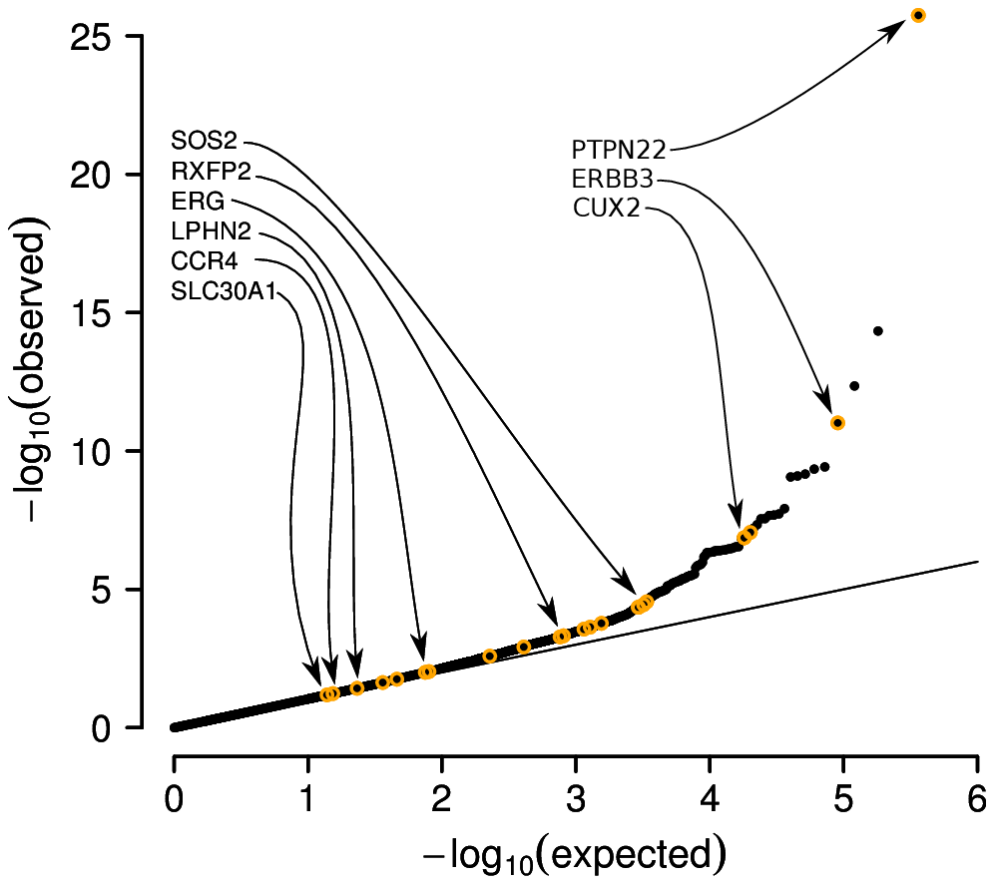
Etiologically relevant and replicated genes identified by 2D-MCP have non-significant p-values by standard single marker analysis. Quantile-quantile (QQ) plot shows results from a single marker analysis of type 1 diabetes from the WTCCC with a subset of hits identified by 2D-MCP highlighted. P-values from the single marker test are shown in black, while each orange circle indicates a region identified as significant by 2D-MCP and its location on the plot is determined by the most significant single marker analysis p-value within 0.1 cM of the significant 2D-MCP hit. Biologically relevant genes identified by 2D-MCP are shown with arrows indicating the most significant association in the region by single marker analysis. Genes shown on the left are only detectable with 2D-MCP, while genes on right are identified by both 2D-MCP and single marker analysis. P-values from the MHC region on chromosome 6 are omitted.

A critical point to note about the performance of our PUMA framework for PMR analysis of GWAS data is that these methods not only result in the correct identification of more loci than a single marker testing analysis (when controlling the false discovery rate at the same level), but also lead to re-orderings of the rank of markers that are considered the most significant when compared to a single marker analysis (Figure 5). As a consequence, we are able to identify etiologically relevant and replicated disease loci that are too weak to be detected by single marker analysis, yet show strong signals of association by PMR analysis. This means that our PMR GWAS analysis is not simply taking advantage of the lower residual variance to improve performance, but is also taking advantage of the fact that conditional correlation of a relevant marker with the phenotype is often more significant than the marginal correlation. When the coefficients for multiple markers, each tagging different susceptibility loci throughout the genome, have nonzero values in the PMR framework, their association with the phenotype becomes more significant. Our framework can therefore identify disease susceptibility loci in a GWAS with weak associations with phenotype, when they are invisible to a single marker testing approach (i.e. they have p-values in a single marker test that would never be considered significant).

### Associations identified by PUMA are concordant with associations from single marker tests

**Figure 6:**
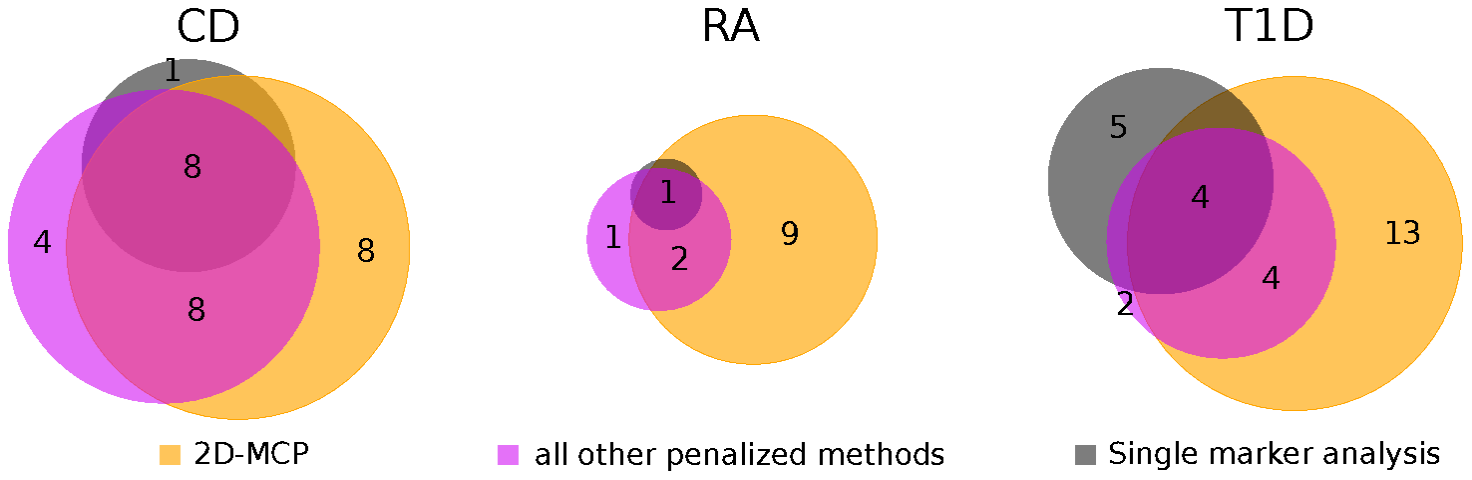
Venn diagrams showing concordance between methods. Venn diagrams show the overall concordance between regions identified by a single marker test, 2D-MCP and the union of Lasso, Adaptive Lasso, NEG, LOG, 1D-MCP and VBAY for Crohn's disease (CD), rheumatoid arthritis (RA) and type 1 diabetes (T1D) for the WTCCC analysis. Areas are approximately proportional to the counts shown and empty regions correspond to a count of zero.

The associations identified by PUMA generally recapitulate associations identified by single marker analysis, and the PUMA hits have perfect concordance for strong associations. Overall 2D-MCP recapitulates the largest number of associations, while the union of the other PMR methods (considered here for illustrative purposes due to the high degree of concordance with each other, and the fact that 2D-MCP identifies almost all of the associations they find) had a lower degree of concordance with the single marker analysis (Figure 4, 6, S14, Table S1). Of the 6 associations identified by a single marker analysis that were missed by our methods, 5 were from type 1 diabetes and 1 was from Crohn's disease (Table S2). One of these associations was borderline significant by 2D-MCP with a p-value of 1.42×10^−7^.

### PUMA methods replicate associations identified by external studies

**Figure 7:**
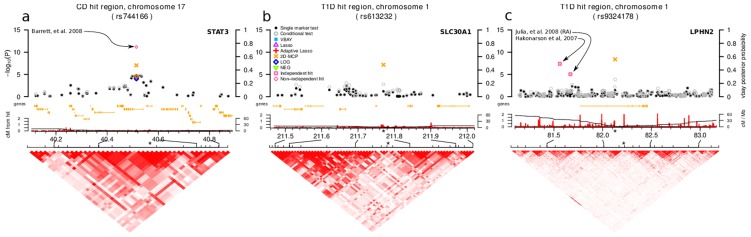
Local manhattan plots illustrating individual examples of associations identified by PUMA analysis of the Wellcome Trust Case Control Consortium (WTCCC) data. The top panel shows 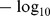 p-values (left axis, all methods except VBAY) and posterior probabilities for VBAY (right axis) for markers in the local genomic region, gene models are shown below in orange with the names of the associated gene indicated, the middle panel shows recombination rates and genetic distance from where the associated marker is indicated with an asterisk and the bottom panel shows a linkage disequilibrium plot among markers in the region using D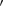. **a**) A region identified only by 2D-MCP replicates an association from a non-independent studies (which included WTCCC data) of Crohn's disease, **b**) a novel association identified for type 1 diabetes only by a PUMA method (2D-MCP) that implicates the etiologically relevant SLC30A1 gene, and **c**) an association identified only by a PUMA method (2D-MCP) for type 1 diabetes that implicated the LPHN2, a gene previously identified but not replicated as a risk locus for type 1 diabetes. Although the associations from the independent studies do not tag the same linkage disequilibrium block as the association identified by 2D-MCP, all three likely affect LPHN2 as they are located either in or directly upstream of this gene and next closest gene is 1.8 Mb (1.7 cM) away.

We compared associations identified by our PUMA methods that were not detected by single marker tests in the WTCCC dataset to markers identified by independent studies in the HuGE database of published GWAS [53] in order to find associations identified in both our analysis and an independent study that did not include WTCCC data. Such replications are considered the gold standard for validating a putative association [54]. In the ideal case the same marker would show an association in both the WTCCC dataset and those summarized in the HuGE database. However, given 1) the lack of overlap of marker-sets between genotyping platforms, 2) that the HuGE database reports only the most significant marker in an associated LD block, and 3) that PMR methods tend to select only a single marker within a LD block, we considered a marker to recapitulate a known association if the two are within 0.1 cM [6]. A representative example from Crohn's disease is shown in Figure 7 where only 2D-MCP is able to identify STAT3 as a susceptibility locus in the WTCCC dataset (Figure 7a). While this association has also been replicated in non-independent datasets [6], which included WTCCC data, the role of STAT3 in Crohn's and other autoimmune disease is well established [55], [56].

While all PUMA methods and a single marker test are able to replicate associations from independent studies, LOG, NEG and 1D-MCP, stood out in terms of identifying associations replicated by non-independent studies, but not detected in the WTCCC dataset by a single marker analysis. These counts reflect the results when the number of markers considered as ‘hits’ was set to be equal across methods so that they reflect the ordering of markers by PMR methods rather than the number of associations. When comparing the total number of significant hits from each method to associations identified in either independent studies or non-independent external studies that incorporated WTCCC data, 2D-MCP is the only PMR method to identify as many total replicated associations as a single marker test (Tables 4, S3, S4, Figures S15, S16, S17, S18, S19). However, 2D-MCP is able to replicate known associations that cannot be replicated by a standard single marker test in this dataset, thus demonstrating that PMR methods can extract biologically relevant information that is overlooked by standard analyses (Table S5). These results demonstrate that PMR methods overall are able to identify replicated associations in this dataset that are invisible to a standard single marker test. Moreover, our methods provide an opportunity to replicate previously unreplicated associations by re-analyzing existing GWAS datasets.

**Table 4:** Number of GWAS associations replicated by each method.

Independent studies									
	Methods								
disease	SMA	VBAY	SMA	Lasso	Adaptive Lasso	LOG	NEG	1D-MCP	2D-MCP
CD	6	4 (0)	6	5 (0)	4 (0)	5 (0)	5 (0)	5 (0)	5 (0)
RA	1	1 (0)	1	1 (0)	1 (0)	1 (0)	1 (0)	1 (0)	1 (0)
T1D	4	2 (0)	4	2 (0)	3 (1)	3 (1)	0 (0)	3 (1)	2 (0)
Total	11	7 (0)	11	8 (0)	8 (1)	9 (1)	6 (0)	9 (1)	8 (0)

Non-independent studies									
	Methods								
disease	SMA	VBAY	SMA	Lasso	Adaptive Lasso	LOG	NEG	1D-MCP	2D-MCP
CD	3	4 (1)	3	2 (0)	2 (0)	2 (1)	5 (3)	3 (2)	6 (3)
RA	0	0 (0)	0	0 (0)	0 (0)	0 (0)	0 (0)	0 (0)	0 (0)
T1D	2	0 (0)	2	0 (0)	1 (0)	1 (0)	0 (0)	1 (0)	2 (1)
Total	5	4 (1)	5	2 (0)	3 (0)	3 (1)	5 (3)	4 (2)	8 (4)
Grand total	16	11 (1)	16	10 (0)	11 (1)	12 (2)	11 (3)	13 (2)	16 (4)

Number of associations identified in re-analysis of WTCCC datasets that replicate associations from either independent or non-independent GWAS. A study is considered to be independent if it does not incorporate data from the WTCCC. An associated marker is considered to replicate a known susceptibility locus if it is within 0.1 cM a marker [6] reported as an association for the same phenotype in the HuGE database. Numbers in parentheses indicate the number of hits that are distinct from those found by the single marker test.

### PUMA methods identify novel associations

Re-analysis of type 1 diabetes, Crohn's disease and rheumatoid arthritis datasets from the original WTCCC [2] with our PUMA methods revealed novel associations that have not been identified in previous GWAS of these diseases (Table 5, Figures S14, S20, S21, S22). These methods, most notably 2D-MCP, identify novel associations in or near genes which have been previously associated with etiologically related diseases or which are known to function in biologically relevant pathways based on public databases and disease literature (Tables 5,6). In addition, PUMA also identified associations without a clear biological link to the disease phenotype (Tables S6, S7, S8).

**Table 5:** Novel etiologically relevant susceptibility loci identified in Wellcome Trust Case Control Consortium (WTCCC) by PUMA methods.

				Method										
disease	SNP	chromosome	position	Single marker analysis	Conditional test	VBAY	Lasso	Adaptive Lasso	LOG	NEG	1D-MCP	2D-MCP	perm-MCP	relevant genes
CD	rs903228	2p16.2	53,692,048	1.71×10^−05^	1.71×10^−05^	0.976	1.79×10^−06^	1.45×10^−06^	4.99×10^−07^	-	7.38×10^−06^	8.01×10^−09^	2.58×10^−06^	ASB3
CD	rs11627513	14q32.2	97,539,170	2.27×10^−05^	1.25×10^−05^	0.983	9.09×10^−06^	1.07×10^−05^	5.61×10^−06^	3.02×10^−05^	2.47×10^−06^	1.06×10^−09^	-	VRK1
CD	rs7497036	15q24.1	74,873,678	1.56×10^−04^	4.3×10^−05^	0.62	1.19×10^−06^	1.39×10^−06^	1.94×10^−06^	6.38×10^−07^	1.73×10^−06^	6.82×10^−07^	-	CYP11A1, SEMA7A
RA	rs12027041	1p36.32	3,591,447	7.55×10^−06^	7.55×10^−06^	0.0383	1.03×10^−04^	2.83×10^−04^	4.04×10^−05^	-	7.4×10^−05^	7.92×10^−08^	-	TP73
T1D	rs613232	1q32.3	211,769,892	6.51×10^−02^	1.71×10^−03^	-	-	-	-	-	-	6.96×10^−08^	-	SLC30A1
T1D	rs4074415	3p22.3	33,161,744	7.68×10^−02^	2.17×10^−03^	-	-	-	1.68×10^−08^	-	-	4.45×10^−08^	-	CCR4
T1D	rs415024	5p15.31	9,392,357	1.69×10^−04^	1.03×10^−04^	0.0364	2.37×10^−05^	1.76×10^−05^	2.1×10^−05^	-	5.83×10^−06^	2.59×10^−09^	-	SEMA5A
T1D	rs9576911	13q13.1	32,329,117	1.4×10^−03^	1.27×10^−06^	0.103	9.75×10^−08^	3.22×10^−08^	6.85×10^−07^	-	4.57×10^−06^	9.45×10^−08^	-	RXFP2
T1D	rs7157296	14q21.3	50,566,881	3.59×10^−05^	1.88×10^−07^	0.906	3.99×10^−07^	6.79×10^−07^	1.67×10^−07^	-	7.72×10^−07^	3.88×10^−10^	-	SOS2
T1D	rs2836631	21q22.2	40,065,905	9.38×10^−03^	4.78×10^−04^	-	-	-	4.9×10^−05^	-	-	1.08×10^−08^	-	ERG

Genes were deemed to be etiologically relevant if they have been previously associated with etiologically related diseases or are known to function in biologically relevant pathways based on public databases and disease literature. The significance of markers with regression coefficient of exactly zero by a penalized maximum likelihood method could not be assessed and are indicated with a dash.

**Table 6:** Novel susceptibility loci identified by PUMA methods and their biological link to the disease.

disease	gene	description
CD	ASB3	functions in ubiquitination and degradation of TNF-R2, which mediates TNF- 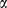 pro-inflammatory response [103], [104]
CD	VRK1	phosphorylates c-Jun and p53, which both function in inflammation [105]
CD	CYP11A1	cytochrome P450 enzyme that synthesizes anti-inflammatory corticosterone in the intestine, and the enzyme is underexpressed in inflamed colon biopsies of patients with Crohn's disease [106], [107]
CD	SEMA7A	immune semaphorin whose expression on activated T-cells induces macrophage production of pro-inflammatory cytokines [108]
RA	TP73	p53-like transcription factors that functions in apoptosis, a process implicated in the etiology of rheumatoid arthritis [109], [110]
T1D	SLC30A1	zinc transporter related to SLC30A8, which has been implicated in type 2 diabetes, and zinc transport plays a role in insulin secretion by pancreatic 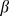 -cells [57], [58]
T1D	CCR4	chemokine receptor and CCR4-bearing T-cells function in the autoimmune inflammation of the pancreas in mice [111]. A nearby marker shows a strong association with celiac disease [112]
T1D	SEMA5A	member of the semaphorin protein family whose members play a role in cell-cell interactions in immune processes, but the function of this gene is not well characterized [108]
T1D	RXFP2	receptor for relaxin, a member of the insulin protein family [113]
T1D	SOS2	Ras-guanine nucleotide exchange factor which is upstream of a number of relevant signalling pathways [114]
T1D	ERG	ETS-family transcription factor that functions in pancreatic development [115]

Genes were deemed to be etiologically relevant if they have been previously associated with etiologically related diseases or are known to function in biologically relevant pathways based on public databases and disease literature.

PUMA methods identified novel susceptibility loci for type 1 diabetes involved in pancreatic function, insulin pathways and immune cell function and for Crohn's disease that are involved in pro- and anti-inflammatory pathways (Table 6). 2D-MCP identified a gene functioning in apoptosis as a susceptibility locus for rheumatoid arthritis (Table 6). These genes are known to function in relevant pathways or have been previously implicated in the etiology of the disease but have not been found by previous GWAS of each disease. A representative example is shown in Figure 7b where only 2D-MCP identifies an association that implicates SLC30A1. This gene is a zinc transporter related to SLC30A8, which has been implicated in type 2 diabetes, and zinc transport plays a role in insulin secretion by pancreatic 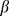-cells [57], [58].

## Discussion

Each GWAS discovery that has a well supported association produces valuable information for understanding the etiology of the disease phenotype and such discoveries are regularly used as the foundation for studies that use the locus as a starting point [59], [60]. Given that GWAS involving a thousand to several thousands of individuals seldom return more than a few to a dozen well-supported associations (depending on the disease) the monetary, time, and resource investment in these studies often translates to a considerable expenditure per discovery. This is true even when considering additional discoveries that may occur as individual GWAS are combined together into large meta-analysis studies [4]–[10]. We have demonstrated that our PUMA framework has the potential to produce added investment return for GWAS studies by discovering additional well-supported disease loci associations that are invisible to the standard single marker analysis methods responsible for almost all reported GWAS [1], [53]. For example, our re-analysis of type 1 diabetes, Crohn's disease and rheumatoid arthritis from the original Wellcome Trust Case Control Consortium (WTCCC) [2] demonstrates that PUMA methods can identify associations that are not detectable by single marker analysis approaches but which replicate associations known from independent studies, which did not include WTCCC data, as well as novel loci with strong links to known disease etiology. These included 10 novel associations identifying genes that are linked to primary pathways of these autoimmune diseases, specifically 6 genes involved in pancreatic function, insulin pathways and immune-cell function in type 1 diabetes; 4 genes (in 3 association regions) functioning in pro- and anti-inflammatory pathways in Crohn's disease; and 1 gene involved in apoptosis pathways in rheumatoid arthritis. Applying our PUMA framework therefore has the potential to add a significant number of discoveries for a given GWAS.

A critical property of our PUMA framework is it does not return the same ordering of significant markers produced by a standard single marker analysis. By simultaneously accounting for the associations of multiple loci and better reflecting the underlying polygenic architecture of complex phenotypes, PUMA can find strong statistical support for associations deemed non-significant by a single marker analysis and places these among the top list of associations. A prime example is marker rs613232 which had a p-value of 6.51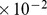 by a single marker analysis in the type 1 diabetes dataset so it would not be considered for a follow-up study. However, by taking into account the polygenic architecture of the trait, 2D-MCP assigned it a p-value of 6.96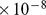 (Figure 7b, Table 5). This marker tags the zinc transporter SLC30A1 and zinc transport has an established role in type 1 diabetes, yet this gene was only identified as a susceptibility locus by 2D-MCP. This example illustrates the power of PUMA methods to reorder the p-values of markers so that a marker that is not in the top 20,000 by a single marker test can be in the top 30 by 2D-MCP. Another example is that of LPHN2, a gene identified by an independent GWAS of type 1 diabetes, yet the association was not replicated in an independent dataset in the same study [61] or, to our knowledge, any subsequent study. In our re-analysis, 2D-MCP identified a strong signal in a nearby marker and assigned it a p-value of 3.99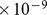, while the p-value by a single marker test was 3.78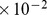 (Figure 7c, Table 5). The very weak single marker p-value found in this dataset makes the previous inability to replicate this association unsurprising. The gene encodes the G-coupled protein receptor latrophilin 2 and has a weak association with rheumatoid arthritis [62] but its relation to disease etiology is unclear. These examples illustrate that our PUMA framework returns additional and complimentary information to the results of a single marker analysis of a GWAS. In general, it seems clear that applying a spectrum of appropriate GWAS analysis methods to the same data is likely to maximize discovery.

The PUMA framework and software that we present here is immediately applicable to a large number of existing GWAS and we are currently exploring extensions of the framework to address additional challenges in GWAS experimental designs and GWAS analysis. For example, GWAS discoveries are regularly being produced by consortia that combine several independently executed GWAS experiments. Such combined data introduce a number of complexities including complex batch effects, population structure, relatedness, and latent environmental variables. While meta-analysis techniques for combining p-values across studies are a good approach to normalizing for many of these issues [4]–[10], a PMR analysis directly on the genotypes can include correction for study heterogeneity, population structure and cryptic relatedness using a linear mixed model [63]–[65], and we are currently working on such extensions. Given that the increase in performance for our PMR methods compared to single marker analysis increases with increasing sample sizes, solving these problems has great potential to detect additional weak associations. There is also going to be a near-term shift towards GWAS that add millions of additional genetic markers genotyped by next-generation sequencing, which can add increased density of markers and different allele types. Our approach can already handle these large number of markers directly to take advantage of the better tagging, and in some cases genotyping, of causal disease polymorphisms. The trend of increased sample sizes and genome marker coverage in GWAS also opens the opportunity to identify genetic interactions that are currently difficult to detect, including epistasis and gene
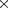
environment interactions, which could be identified by incorporating group penalty approaches [30], [66], [67] within our framework. Overall, our framework represents a platform for integrating richer statistical models and techniques for addressing the future needs of GWAS.

## Methods

### The PUMA framework

Our framework is a combination of algorithms and heuristic approaches designed for robust and efficient analysis of GWAS datasets when the desired output is a ranked list of genetic markers that individually tag disease loci. The value of the framework is that tag genetic markers, which are too weak to be reliably identified by a single marker analysis, can be identified while preserving a conservative FDR genome-wide. To solve issues that have limited the value of existing PMR GWAS software for this purpose, we designed our framework to have the following properties: 1) the versatility to handle a diversity of penalties for simultaneous analysis of thousands to millions of genetic markers while incorporating unpenalized covariates, 2) the efficiency to analyze up to millions of markers after pre-screening on a standard desktop, 3) the sensitivity to tune the strength of penalties and to perform model selection when the fraction of variation accounted for by disease loci identifiable with tag markers is small (as is typical for GWAS), and 4) the capability to return a ranked list of p-values where each of the top markers identifies an independent disease association. We outline the components of our framework responsible for each of these properties in the next four sections, followed by a description of our software PUMA that implements our recommended practices and options for implementation. We note that in its entirety, this framework is a new GWAS analysis approach that incorporates novel components including (but not limited to): application of penalties not previously applied to GWAS, a new MM algorithm for GLMs, heuristics for penalty strength and model selection, and *post hoc* model fitting approaches for ranking associated markers.

### Objective functions and penalties

Our framework makes use of a generalized linear modeling (GLM) framework to construct the likelihood objective function. We can therefore model phenotypes measured on a large diversity of scales by implementing an appropriate link function [45], although here, we limit our implementations to an identity and logistic link function to model continuous phenotypes with normal error and case-control phenotypes, respectively. We also note that incorporating unpenalized covariates is straightforward, where these are modeled with regression coefficients with no penalty. While our current implementation is restricted to penalties that select a small number of well supported markers (i.e. feature selection penalties [51], [68]–[72]), the framework is versatile enough to implement a wide diversity of penalties approaches when making use of the algorithm described in the next section.

For marker selection, we use the penalized maximum likelihood estimate (pMLE) of the regression coefficients:
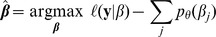
where 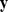 is the vector of disease phenotype values, and 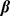 is the vector of regression coefficients 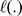 is the log-likelihood of a linear or logistic regression and 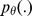 is the penalty function on the magnitude of 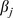 indexed by a vector of tuning parameters, 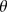. Since we are interested in identifying a small set of variables associated with the phenotype, the penalty function must have the sparsity property whereby most of the regression coefficients are set to exactly zero. Multiple penalties satisfy this condition while balancing computational tractability with desirable theoretical properties. We implement the penalties that have been applied for PMR GWAS analysis (i.e. Lasso, Adaptive Lasso, NEG, MCP) as well as a penalty that has not be previously applied to GWAS (i.e. LOG). We describe the properties of each penalty in the following paragraphs and the functional form of each penalty is given in Text S2.

The Lasso penalty [68] (previously implemented for PMR GWAS in the software Mendel [31]) is a linear function of the magnitude of the regression coefficients and is the most widely used since it has a single tuning parameter. Moreover, the penalty is convex so that it yields a unique pMLE. Yet it is widely known to select too many variables with non-zero coefficients in high dimensional datasets [72] and does not satisfy the “oracle property” whereby parameter estimates are asymptotically equivalent to unpenalized estimates when the identity of the non-zero coefficients are known in advance [72].

The Adaptive Lasso penalty [69], (previously implemented for PMR GWAS by Yang, et al. [37]) unlike the Lasso, satisfies the oracle property. This two-step Lasso regression procedure is also convex (yields a unique pMLE) although it requires an initial estimate of the regression coefficients, which are then used to weight the strength of a Lasso penalty in the second step. There is no criterion for determining optimal weights, so in practice the Lasso penalty for each coefficient is weighted by the square root of the initial coefficient estimate.

The NEG penalty [71] (previously implemented for PMR GWAS in the software HyperLasso [22]) has two tuning parameters and is non-convex such that it produces a multimodal likelihood surface where pMLE's are not unique. The penalty satisfies the oracle property, since the derivative of the penalties approach zero in the limit [72], although it has other less desirable properties since its derivative approaches zero much more slowly than the other penalties and its very complex functional form makes it numerically unstable for large coefficient values. In our framework, we re-implement NEG using a faster algorithm than Hoggart, et al. [22] and includes a two dimensional search of the tuning parameter space, while Hoggart, et al. [22] use asymptotic theory to set the tuning parameters.

The MCP penalty [51] (previously implemented in the R package *grpreg* [73]) has two tuning parameters. Like NEG, this penalty is non-convex and satisfies the oracle property. However, the derivative of MCP reaches zero for finite coefficient values so that it avoids over penalizing large coefficient values. Moreover, MCP is designed to reduce the multimodality of the objective function [51]. The tuning parameters determine the slope of the penalty near the origin (i.e. 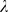) and coefficient value at which the derivative of the penalty is set to zero (i.e. 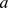 or 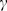, depending on notation). When applying this method to GWAS data, Ayers and Cordell [32] fixed the value of 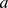 at 30, and identify the value of 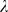 using a permutation approach. We term this approach perm-MCP. In addition, we consider a one dimensional search over the value of 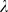 and a two dimensional search over both parameters, termed 1D-MCP and 2D-MCP, respectively. The latter has the most potential since it explores the value of the tuning parameter, 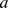, that determines the coefficient value at which the derivative of the penalty is set to zero, and learns the value of the parameter based on the data.

We also implement the LOG penalty and apply it to GWAS for the first time. The LOG penalty [70] has two tuning parameters and is non-convex, such that it produces a multimodal likelihood surface where pMLE's are not unique, but is satisfies the oracle property, since the derivatives of the penalties approach zero in the limit [72]. This penalty is also designed to identify fewer non-zero regression coefficients.

### Minorize-Maximization (MM) algorithm and scalable implementation

Our framework implements a highly efficient algorithm and optimized coding practices to allow fast simultaneous analysis of genetic markers in the range of hundreds of thousands to millions. We implement a new minorize-maximization (MM) algorithm for finding pMLE by using a coordinate-wise ascent approach with an upper bound on the second derivative of the likelihood function [44]. By using bounded univariate updates, the algorithm is extremely fast and is guaranteed to converge to a mode of the likelihood surface. While Newton-Raphson algorithms must evaluate the log-likelihood after each update to check if it has decreased [74], the use of an MM algorithm for logistic regression guarantees a monotonic increase and eliminates the expensive function evaluation. Derivations are given in Text S2. In addition to the algorithm, we also implement a number of optimized coding practices to accelerates these PMR methods. These include data structures to minimize access time to each marker, use of optimized linear algebra libraries and searching multiple modes of the likelihood surface for non-convex penalties in parallel.

### Adaptive tuning of penalties and model selection

Critical to the performance of our framework is preserving a conservative control of the FDR for identified markers. To accomplish this, we employ a strategy that allows our PMR methods to automatically adapt, not only to the sample size of the dataset, but also to the number and magnitude of the non-zero regression coefficients for relevant markers associated with the phenotype. Our approach includes an adaptive tuning of penalty strength in combination with model selection and assessment of model fit.

Statistical theory considering linear regression has shown that for a sample size of 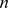, the number of variables detectable as having nonzero coefficients is on the order of 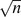 [75]. This is consistent with other theoretical work [76], [77] and satisfies our intuition that the number of detectable associations is directly related to the sample size of the dataset. For adaptive tuning of the Lasso and Adaptive Lasso, where the likelihood is convex and there is a single tuning parameter, the search is simple and we start with a severe penalty, which is gradually decreased to select one additional non-zero coefficient at a time, until 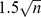 genetic markers are selected. For the non-convex penalties [22], [32], [70], a grid search over a two-dimensional space of tuning parameters is used starting from equally spaced Lasso models (a special case of all non-convex penalties) where the non-convexity of the penalty is gradually increased until 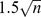 markers are selected. This approach for searching the space has been shown to avoid some suboptimal modes of the likelihood surface [70]. We note that we have previously published the approximate Bayesian methods, VBAY and VBAYNET, which incorporate a probabilistic bound, where we applied the same 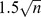 bound [33], [78]. In order to mitigate the problem of suboptimal modes at least to some degree, we explore multiple modes of the likelihood surface for non-convex penalties by permuting the order in which the regression coefficients are updated. For both the simulations and WTCCC analyses of this study, we found 100 reorderings was sufficient to obtain robust results.

Once sets of markers with nonzero coefficients are identified for each value of the tuning parameters for a given penalty, the optimal set is determined. We assessed the overall appropriateness of the fit of a selected model based on a QQ plot by fitting an unpenalized model with selected markers, and calculating p-values for each marker in the dataset by regressing it against the residuals from the first step (Figure S12) [79].

A slight inflation of 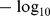 p-values at the tail of the QQ plot indicates that the PMR method has not over fit the data. We tried many model selection strategies to determine which consistently produced optimal residual-QQ plots, including Bayesian information criterion [80], cross-validation [40], asymptotic justifications [22] and permutation-based approaches [32]. We eventually selected AIC [81] with an additional restriction as our default because it tended to produce the best overall performance for simulated GWAS data, when assessed by both QQ plot and when applying a strong control of the FDR. While AIC [81] and BIC [80] have been studied extensively in the context of sparse model selection theory, these criteria do not incorporate an upper bound on the number of variables it is feasible to select for a given sample size. Therefore, while good performance may be guaranteed for an infinite sample size, these criteria can select too many variables than is plausible for a finite sample size [82] such that our upper bound on the number of markers selected 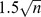 seems reasonable, where we compare all models satisfying this bound using AIC. We also note that while AIC is not asymptotically model selection consistent [83], our use of AIC should not suffer from the inclusion of irrelevant genetic markers since we apply it in the context of models constrained to have fewer than 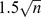 genetic markers and we apply a significance test (described in the next section) before considering a marker to have an association.

### *Post hoc* assessment of p-value ranks

The most valuable final output of a PMR GWAS analysis is a ranked list of markers in decreasing order of highest confidence. Standard methods for producing such ranked lists in a PMR framework assess significance by conducting variable selection on a subset of the data and assessing significance on another subset [84], or subsetting the data many times and identifying variables selected in many of the subsets [85]. Such methods can be very computationally demanding for large GWAS datasets and have been shown to underperform a standard single marker test of association [86]. Moreover, these methods do not address the challenging problem of assessing the significance of a marker in the presence of correlated markers within the same linkage disequilibrium (LD) block. While PMR methods tend to select a single non-zero regression coefficient for an associated LD block, cases often arise where multiple markers in a LD block have non-zero coefficients. In such cases, the correlation between the markers in the block dramatically increases the sample variance of the coefficients so that the markers are not assigned significant p-values even if each would be significant if the other were dropped from the model.

We deal with the problem of producing an informative ranked list of selected markers in our framework by initially fitting an unpenalized regression model with all markers selected by a given PMR and including relevant covariates, and calculating p-values for each marker using a standard likelihood ratio test by comparing the full model to null models where each marker is omitted in turn. The correlation between all pairs of selected markers is then evaluated, the pair of markers with the largest correlation is identified, the marker with the smallest absolute regression coefficient of the two is dropped, and p-values are then recalculated for the remaining markers. This process is repeated until no pairwise correlation between remaining markers exceeds 0.1 and each marker is finally assigned the smallest p-value produced for it during this processes. This heuristic procedure means that the values cannot be interpreted strictly as asymptotic p-values [31], but they can be considered as scores indicative of the significance of the association that ranks the values in terms of confidence while ensuring at least one marker in the LD block is assigned a p-value rank that can appropriately reflect a true association.

### PUMA software

PUMA implements both linear and logistic models for PMR methods, as well as single marker analysis, a conditional regression analysis, and the variational Bayes multiple regression method VBAY [33]. The software reads genotype files in TPED format and phenotypes in TFAM as used by Plink [87]. For all multiple marker methods, we employ a heuristic to remove markers with low marginal correlations with the phenotype as they are extremely unlikely to be selected to have nonzero regression coefficients [31], [46]. This is approach is not novel [31], [46], but accelerates computation and allows flexibility when analyzing extremely large GWAS datasets. The set of markers identified at each mode of the likelihood surface is stored and is saved in a text file readable by R. The software is available at http://mezeylab.cb.bscb.cornell.edu/Software.aspx.

For the single marker analysis, p-values are calculated using a standard F-test or likelihood ratio test [88] for linear and logistic models, respectively. The conditional regression performs the standard single marker test of association and includes the significant markers as covariates in a second set of single marker tests. The minimum of the two p-values from the first and second stage analysis is then reported for each marker. In this analysis we used a first stage p-value cutoff of 1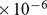 and selected the single most strongly associated marker within 100 Kb to include as covariates in the second stage. We also re-implemented the approximate Bayesian method VBAY, previously developed by our group [33]. While Bayesian regularized regression methods using a number of prior distributions as been applied to association mapping using Markov chain Monte Carlo (MCMC) methods, these cannot simultaneously analyze more than a few hundred to a few thousand genetic markers at a time [34], [35]. VBAY [33] uses a variational Bayes approximation to the posterior surface [89] and applies a hierarchical mixture prior on the regression coefficients so that the large majority of coefficients have a high posterior probability of being exactly zero (see Logsdon et al. [33] for a more detailed discussion of this method). Within PUMA, we re-implemented VBAY and added the capability to analyze case-control phenotypes, where we increased the power to detect weak associations for case-control phenotypes by approximating a logistic regression by modeling the error distribution with a Student t-distribution with 7.3 degrees of freedom. This parameterization has the smallest squared error loss of any t-distribution with respect to the logistic error function [90], [91]. Moreover, we address the multimodality of the posterior surface by exploring many posterior modes and applying Bayesian model averaging [92] in order to weight the contribution of each mode to the posterior probability of association for each marker. The VBAY algorithm was run with 1000 restarts to explore the non-convex posterior surface. Due to its Bayesian formulation, VBAY reports the posterior probability, between 0 and 1, that each marker is associated with the phenotype.

### PUMA software recommended usage

The default settings of PUMA are our recommended settings for a GWAS analysis, which are the same procedures we followed for our analysis of the WTCCC data here:

1. Marker imputation: Beagle [93] was used to impute missing genotypes, but other methods can be used. Alternatively, PUMA fills in missing data with the mean of each marker.
2. Inclusion of fixed covariates: Identify relevant covariates and principal components and perform single marker analysis so that the corresponding QQ plot and 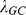 [94] are acceptable. Results from the single marker analysis must be acceptable before applying PMR methods.
3. Marker filtering: We applied a pre-screening filter based on p-values from single marker analysis using a cutoff of 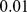.
4. Number of restarts: The penalized likelihood for the 1D-MCP, 2D-MCP, NEG and LOG penalties is non-convex so 100 reorderings were used to explore the multimodal surface for each setting of the tuning parameters.
5. Performance assessment: We recommend assessing the fit of a PMR model by including the selected markers as covariates in a subsequent single marker analysis. Too much inflation or deflation of the p-values indicates that the PMR method may be overfitting the data.
6. Threshold determination: We have demonstrated that the reported p-value score statistics are valuable at prioritizing the top hits as well as novel weak associations, so assessing the list in rank order is the suggested strategy for minimizing false positives. For example, in our current analyses we examined at most the top 30 hits for each method combined across the three diseases, which limited our focus to markers with a p-value score of 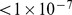 for 2D-MCP, 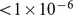 for all other PMR methods, and for comparison, a posterior probability of 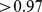 for VBAY.

### GWAS simulation study

Our approach was to simulate different sized GWAS experiments where we used the real genetic markers for unrelated European individuals from the Multi-Ethnic Study of Atherosclerosis (MESA) [95] genotyped on the Affymetrix 6 platform. Larger sample sizes were generated by drawing haplotypes from existing individuals in order to avoid the confounding effect of population structure [96]. For each simulated GWAS dataset, we considered different sample sizes (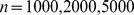) with equal numbers of case and control phenotypes simulated under an additive threshold model with a disease prevalence of 50%, using the GCTA program [97], and that different numbers of susceptibility loci (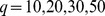) contributed to phenotype heritability, where the total contribution of these loci to heritability was varied (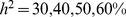). Coefficients were drawn from a 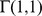, independent of allele frequency, so that most effect-sizes were very small as determined by the marginal heritability calculated by GCTA [97]. We considered 20 replicates per simulation condition to give 960 simulated GWAS datasets. Causal variants affecting the phenotype were selected uniformly from the set of genetic markers with minor allele frequency (MAF) 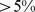. We followed typical array-based GWAS by omitting the causal variants from the analysis so a susceptibility locus must be identified by markers in linkage disequilibrium with the causal variant.

Following the performance evaluation of previous studies [22], [32], a marker was considered a true positive hit if it had 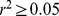 with a causal marker, otherwise it was considered a false positive hit. Since a causal variant may be tagged by multiple true positive markers, the true positive count is defined as the total number of causal variants tagged when all true positive hits are considered together. Alternatively, since false positive hits will often fall in clusters in the same linkage disequilibrium block, we assign each to a 100 kb cluster centered at the most significant hit in that cluster. The false positive count is then defined as the number of such false positive clusters. We note this is a strategy for assessing the performance properties that will be of greatest interest to GWAS practitioners since it focuses on correct identification of tag markers that are in high linkage disequilibrium (LD) and in close physical proximity to the location of the true causal alleles, while considering a strict control of the FDR.

### Analysis of WTCCC data

To run the data analysis we used the same quality control filters as in the Wellcome Trust Case Control Consortium, first by excluding 809 individuals because of poor sample quality, non-Caucasian ancestry, or a high degree of relatedness [2]. An additional individual was removed for being an outlier by principal components analysis [96]. Marker locations and genetic map are based on reference assembly GRCh37/hg19 and dbSNP v131. Next, the same study-wide missing data rate and deviation from Hardy-Weinberg equilibrium cutoffs were used for each set of cases as in the original study [2], with an additional filter to only include markers in the analysis with a minor allele frequency greater than 0.05 in each combined case-control population, leaving approximately 360,000 markers for each combined case-control data set. We used the CHIAMO calling scores to set data to missing, where any call with a score of less than 0.90 was set to missing [2]. To impute this sporadic missing data we used Beagle [93], with the default settings and allocating a maximum of 3000 MB of memory, where the sporadic missing data for each cohort was imputed separately. The same set of controls (1958 Birth Cohort (58C) and UK Blood Service sample (NBS)) were used for each set of cases as in the original study [2]. Finally, the PMR and single marker analyses included sex as a covariate along with the first two principal components of the genotype matrix.

In order to explore the biological function, relevant pathways and possible disease implications of each gene near a significantly associated marker, we mined public databases including GenBank [98], Pfam [99], KEGG [100], OMIM [101], GeneCards [102] as well as the HuGE database [53] of known GWAS hits and known gene-phenotype links. We also conducted an extensive literature search with each gene name and relevant phenotypes.

## Supporting Information

Figure S1Assessing p-value cutoff in two-step forward regression. Plots show 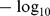 p-values from a single marker analysis (x-axis) compared to the change in 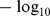 p-values from a conditional regression analysis where markers passing the Bonferroni cutoff are included as covariates (y-axis). Markers passing the Bonferroni cutoff in the first step (red points) are necessary omitted from being tested in the second step, and are considered to have no change in p-value. Markers with a large enough increase in 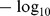 p-value in the second step to cross the second Bonferroni cutoff (blue dashed line) are indicated by green points. The p-value cutoff of 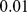 (i.e. a 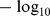 p-value of 2) is indicated by the grey dashed line. Results are shown for 10 replicate simulations each of (a) 1000, (b) 2000, and (c) 5000 samples with 500 K markers, heritability of 30, 40, 50 or 60% and 30, 40, 50, 70 or 100 simulated markers with true nonzero coefficients. This corresponds to 200 simulations and 1 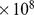 p-values for each sample size. The results indicate that in a forward regression, which approximates penalized multiple regression [49], [50], markers with small 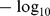 p-values in the first step have a very low probability of being significant in the second step. Therefore, using a p-value cutoff of 0.01 from a marginal regression retains almost all relevant variables under biologically motivated simulation conditions.(PDF)

Figure S2 Effect of pre-screening on performance. (a) 100 replicate simulations with 500 K markers, 50 causal markers, heritability of 50% and 1000 or 2000 individuals using Lasso and MCP methods show that using a pre-screening p-value cutoff of 1, 0.10 and 0.01 has no noticeable effect of performance of PUMA. Note that the performance was so similar for all cutoffs that the curves are overlapping. (b) Running times for simulations in (a) show that pre-screening substantually reduces computational time. We note that simulations with 5000 individuals were not possible due to the very high memory requirements of running PUMA without prescreening.
(PDF)

Figure S3 Power vs sample size. Simulation results showing power for our PMR methods, current PMR methods, an approximate Bayesian method, single marker analysis and conditional regression methods at an FDR of 5% as a function of sample size as in Figure 3c in the main text. Results are shown for a range of total heritabilities and number of susceptibility loci.
(PDF)

Figure S4 Power vs number of causal markers. Simulation results showing power for our PMR methods, current PMR methods, an approximate Bayesian method, single marker analysis and conditional regression methods at an FDR of 5% as a function of the number of susceptibility loci. Results are shown for a range of sample sizes and total heritabilities.
(PDF)

Figure S5 Power vs marginal heritability for 1000 samples. Simulation results showing power a sample size of 1000 for our PMR methods, current PMR methods, an approximate Bayesian method, single marker analysis and conditional regression methods at an FDR of 5% as a function of the marginal heritability of each causal marker as in Figure 3a in the main text. Results are shown for a range of total heritabilities and number of causal markers.
(PDF)

Figure S6 Power vs marginal heritability for 2000 samples. Simulation results showing power a sample size of 2000 for our PMR methods, current PMR methods, an approximate Bayesian method, single marker analysis and conditional regression methods at an FDR of 5% as a function of the marginal heritability of each causal marker as in Figure 3c in the main text. Results are shown for a range of total heritabilities and number of causal markers.
(PDF)

Figure S7 Power vs marginal heritability for 5000 samples. Simulation results showing power a sample size of 5000 for our PMR methods, current PMR methods, an approximate Bayesian method, single marker analysis and conditional regression methods at an FDR of 5% as a function of the marginal heritability of each causal marker as in Figure 3c in the main text. Results are shown for a range of total heritabilities and number of causal markers.
(PDF)

Figure S8 Precision-Recall curves for 1000 samples. Simulation results showing precision-recall curves for a sample size of 1000. Results are shown for a range of total heritabilities and number of causal markers. Solid colors for pML methods indicate results using our method for assessing significance in the presence of correlated markers, while dashes indicate the significance method of Wu, et al. [31] and perm-MCP [32]. This figures is analogous to Figure 3b in the main text.
(PDF)

Figure S9 Precision-Recall curves for 2000 samples. Simulation results showing precision-recall curves for a sample size of 2000. Results are shown for a range of total heritabilities and number of causal markers. Solid colors for pML methods indicate results using our method for assessing significance in the presence of correlated markers, while dashes indicate the significance method of Wu, et al. [31] and perm-MCP [32]. This figures is analogous to Figure 3b in the main text.
(PDF)

Figure S10 Precision-Recall curves for 5000 samples. Simulation results showing precision-recall curves for a sample size of 5000. Results are shown for a range of total heritabilities and number of causal markers. Solid colors for pML methods indicate results using our method for assessing significance in the presence of correlated markers, while dashes indicate the significance method of Wu, et al. [31] and perm-MCP [32]. This figures is analogous to Figure 3b in the main text.
(PDF)

Figure S11Precision-Recall curves for perm-MCP for multiple values of eFPR and pre-screening p-value cutoff. Simulations for 5000 samples, 20 causal markers and heritability of 50% using eFPR values (1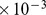, 1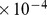, 1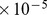, 1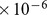, 1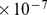) and pre-screening cutoff values (0.1, 0.01, 0.001) indicated in the legend. Results from single marker analysis and MCP-2D are shown for comparison.(PDF)

Figure S12 Quantile-Quantile plots for each disease and method. Plots are shown for **a**) Crohn's disease, **b**) Rheumatoid arthritis and **c**) Type 1 diabetes. Results from a standard single marker analysis of each disease are shown in grey and are the same in all plots for a given disease. Results from including the subset of significantly associated markers identified by each pML method as covariates in a single marker analysis of remaining markers is shown in black, where the relevant method is indicated above each plot. Results from replacing the p-values from this latter analysis with p-values from the PMR method for the relevant markers with nonzero coefficients are shown in color. The genomic inflation values for are shown in the upper left of each plot. Note that the NEG method failed for the type 1 diabetes dataset, so no plot is shown.
(PDF)

Figure S13Manhattan plots of single marker analysis for three disease datasets. Manhattan plots showing results of single marker analysis for **a**) Crohn's disease, **b**) Rheumatoid arthritis, and **c**) Type 1 diabetes datasets from our re-analysis. Shown are 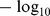 p-values where large values are truncated at 20. Markers with 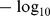 p-values 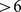 are colored green.(PDF)

Figure S14 Genome-wide plots of hits found by each method. Genome-wide plot of associations identified by analyzing the WTCCC data for **a**) Crohn's disease, **b**) rheumatoid arthritis and **c**) type 1 diabetes using PMR methods, conditional regression, and single marker analysis. External associations from independent datasets (which do not include WTCCC data) and non-independent datasets (which include WTCCC data) of the same disease are indicated with pink boxes and diamonds, respectively. Markers that are considered associations only when the p-value threshold for the single marker analysis is relaxed to match the same number of associations (with hits in the MHC region excluded) as the union of all PMR methods are indicated with black circles. Arrows indicate novel associations that are biologically interpretable.
(PDF)

Figure S15 Local manhattan plots of hits replicated from an independent study of Crohn's disease.
(PDF)

Figure S16 Local manhattan plots of hits replicated from an independent study of rheumatoid arthritis.
(PDF)

Figure S17 Local manhattan plots of hits replicated from an independent study of type 1 diabetes.
(PDF)

Figure S18 Local manhattan plots hits replicated from a non-independent study of Crohn's disease.
(PDF)

Figure S19 Local manhattan plots of hits replicated from a non-independent study of type 1 diabetes.
(PDF)

Figure S20 Local manhattan plots of biologically relevant hits for Crohn's disease.
(PDF)

Figure S21 Local manhattan plots of biologically relevant hits for rheumatoid arthritis.
(PDF)

Figure S22 Local manhattan plots of biologically relevant hits for type 1 diabetes.
(PDF)

Table S1Concordance of PMR hits with single marker analysis. Number of regions identified by a single marker analysis with a p-value
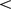
1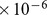 and the number of these regions that are recapitulated by each other method.(PDF)

Table S2Regions identified only by single marker analysis. Regions identified by single marker analysis with a p-value
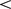
1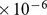. Of the regions missed by PMR methods, only one association passes the Bonferroni cutoff of 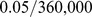 = 1.38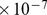, although 5 of these have been replicated: rs6596075 [4], [116], [117], rs17388568 [6], rs10807124 [6], [61], [118], rs4766517 [6], [61], [118], rs12924729 [6], [61], [118], [119].(PDF)

Table S3Associations recapitulated in independent studies. Regions which are significant either by single marker analysis, conditional regression, or a PMR method and which recapitulate a known association to the same disease in an independent study that does not include data from the WTCCC. The table includes all regions with a VBAY posterior probability
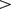
0.97, an MCP p-value
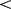
1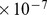, or a p-value for any other method 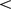
1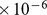.(PDF)

Table S4Associations recapitulated in non-independent studies. Regions which are significant either by single marker analysis, conditional regression, or a PMR method and which recapitulate a known association to the same disease in a non-independent study that includes data from the WTCCC. The table includes all regions with a VBAY posterior probability 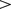
0.97, an MCP p-value
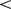
1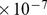, or a p-value for any other method 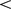
1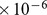.(PDF)

Table S5 Replication counts for equal numbers of hits. For each method, the number of associations in this re-analysis that replicate associations identified in **a**) independent (not including WTCC data) and **b**) non-independent (including WTCC data) datasets, where the number of markers considered as ‘hits’ is set to be equal across methods. For each method the number of hits is set to a given value and the number of replications is reported. Numbers in parentheses indicate the number of hits that are distinct from those found by the single marker analysis.
(PDF)

Table S6 Additional associations for Crohn's disease. Additional associations for Crohn's disease identified by PMR methods but not a single marker analysis.
(PDF)

Table S7 Additional associations for rheumatoid arthritis. Additional associations for rheumatoid arthritis identified by PMR methods but not a single marker analysis.
(PDF)

Table S8 Additional associations for type 1 diabetes. Additional associations for type 1 diabetes identified by PMR methods but not a single marker analysis.
(PDF)

Text S1 Parameters for running HyperLasso.
(PDF)

Text S2 Efficient coordinate-wise gradient descent algorithms for high-dimensional penalized generalized linear models with convex or nonconvex penalties.
(PDF)
