# Supplementary material for: PUMA: A Unified Framework for Penalized Multiple Regression Analysis of GWAS Data
Source: PLoS Comput Biol. 2013 Jun 27;9(6):e1003101. doi: 10.1371/journal.pcbi.1003101 (PMC3694815; doi:10.1371/journal.pcbi.1003101)

Figure S1: Assessing p-value cutoff in two-step forward regression. Plots show $-\log_{10}$ p-values from a single marker analysis (x-axis) compared to the change in $-\log_{10}$ p-values from a conditional regression analysis where markers passing the Bonferroni cutoff are included as covariates (y-axis). Markers passing the Bonferroni cutoff in the first step (red points) are necessary omitted from being tested in the second step, and are considered to have no change in p-value. Markers with a large enough increase in $-\log_{10}$ p-value in the second step to cross the second Bonferroni cutoff (blue dashed line) are indicated by green points. The p-value cutoff of 0.01 (i.e. a $-\log_{10}$ p-value of 2) is indicated by the grey dashed line. Results are shown for 10 replicate simulations each of (a) 1000, (b) 2000, and (c) 5000 samples with 500K markers, heritability of 30, 40, 50 or 60% and 30, 40, 50, 70 or 100 simulated markers with true nonzero coefficients. This corresponds to 200 simulations and 1×10^8 p-values for each sample size. The results indicate that in a forward regression, which approximates penalized multiple regression [5, 11], markers with small $-\log_{10}$ p-values in the first step have a very low probability of being significant in the second step. Therefore, using a p-value cutoff of 0.01 from a marginal regression retains almost all relevant variables under biologically motivated simulation conditions.

(a) 1000 samples

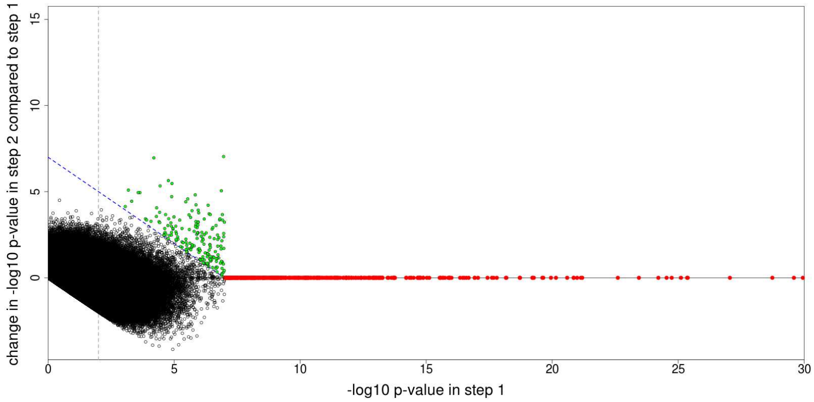

(b) 2000 samples

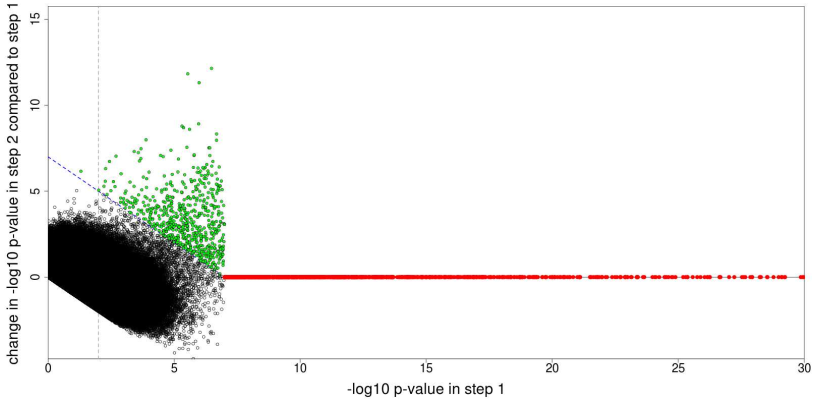

(c) 5000 samples

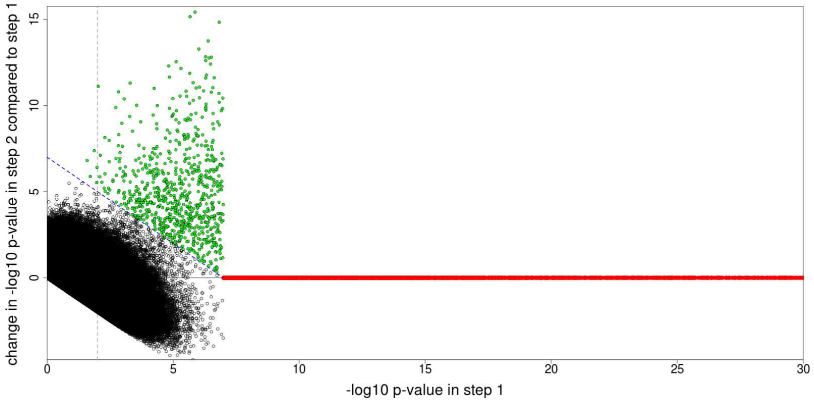

Supplement: Figure S1 — Assessing p-value cutoff in two-step forward regression. Plots show p-values from a single marker analysis (x-axis) compared to the change in p-values from a conditional regression analysis where markers passing the Bonferroni cutoff are included as covariates (y-axis). Markers passing the Bonferroni cutoff in the first step (red points) are necessary omitted from being tested in the second step, and are considered to have no change in p-value. Markers with a large enough increase in p-value in the second step to cross the second Bonferroni cutoff (blue dashed line) are indicated by green points. The p-value cutoff of (i.e. a p-value of 2) is indicated by the grey dashed line. Results are shown for 10 replicate simulations each of (a) 1000, (b) 2000, and (c) 5000 samples with 500 K markers, heritability of 30, 40, 50 or 60% and 30, 40, 50, 70 or 100 simulated markers with true nonzero coefficients. This corresponds to 200 simulations and 1 p-values for each sample size. The results indicate that in a forward regression, which approximates penalized multiple regression [49], [50], markers with small p-values in the first step have a very low probability of being significant in the second step. Therefore, using a p-value cutoff of 0.01 from a marginal regression retains almost all relevant variables under biologically motivated simulation conditions. (PDF) [file pcbi.1003101.s001.pdf]
