# Supplementary material for: PUMA: A Unified Framework for Penalized Multiple Regression Analysis of GWAS Data
Source: PLoS Comput Biol. 2013 Jun 27;9(6):e1003101. doi: 10.1371/journal.pcbi.1003101 (PMC3694815; doi:10.1371/journal.pcbi.1003101)

Figure S2: **Effect of pre-screening on performance.** (a) 100 replicate simulations with 500K markers, 50 causal markers, heritability of 50% and 1000 or 2000 individuals using Lasso and MCP methods show that using a pre-screening p-value cutoff of 1, 0.10 and 0.01 has no noticeable effect of performance of PUMA. Note that the performance was so similar for all cutoffs that the curves are overlapping. (b) Running times for simulations in (a) show that pre-screening substantually reduces computational time. We note that simulations with 5000 individuals were not possible due to the very high memory requirements of running PUMA without prescreening.

(a) Precision-Recall curves

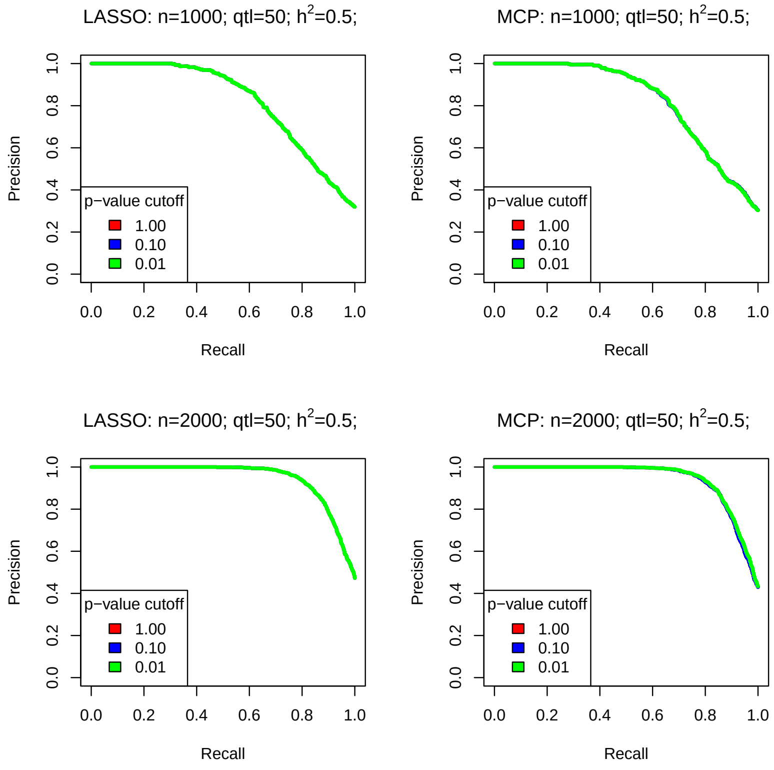

(b) Run times for each prescreening cutoff

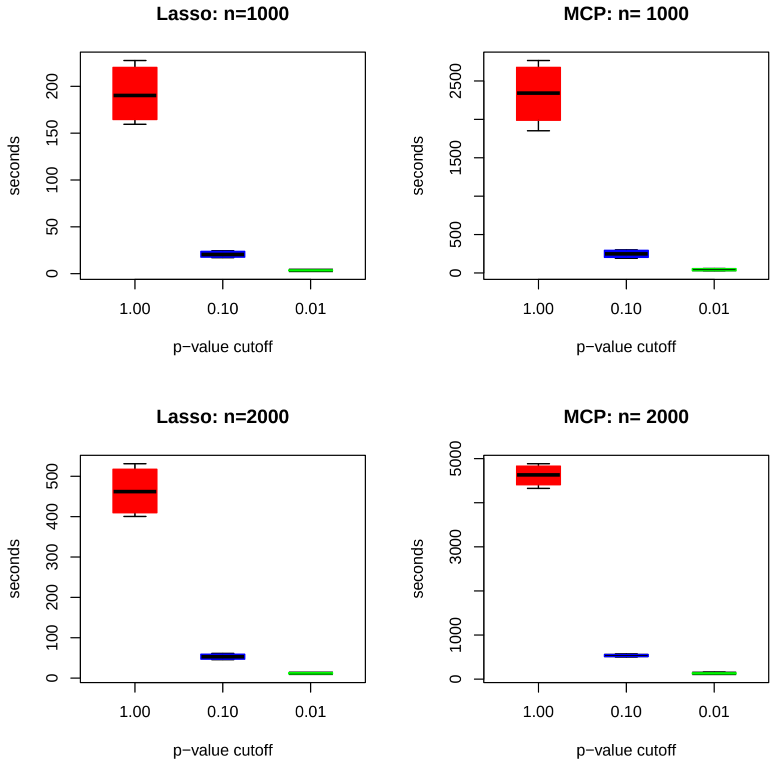

Supplement: Figure S2 — Effect of pre-screening on performance. (a) 100 replicate simulations with 500 K markers, 50 causal markers, heritability of 50% and 1000 or 2000 individuals using Lasso and MCP methods show that using a pre-screening p-value cutoff of 1, 0.10 and 0.01 has no noticeable effect of performance of PUMA. Note that the performance was so similar for all cutoffs that the curves are overlapping. (b) Running times for simulations in (a) show that pre-screening substantually reduces computational time. We note that simulations with 5000 individuals were not possible due to the very high memory requirements of running PUMA without prescreening. (PDF) [file pcbi.1003101.s002.pdf]
