# Supplementary material for: PUMA: A Unified Framework for Penalized Multiple Regression Analysis of GWAS Data
Source: PLoS Comput Biol. 2013 Jun 27;9(6):e1003101. doi: 10.1371/journal.pcbi.1003101 (PMC3694815; doi:10.1371/journal.pcbi.1003101)

**Figure S4: Power vs number of causal markers.** Simulation results showing power for our PMR methods, current PMR methods, an approximate Bayesian method, single marker analysis and conditional regression methods at an FDR of 5% as a function of the number of susceptibility loci. Results are shown for a range of sample sizes and total heritabilities.

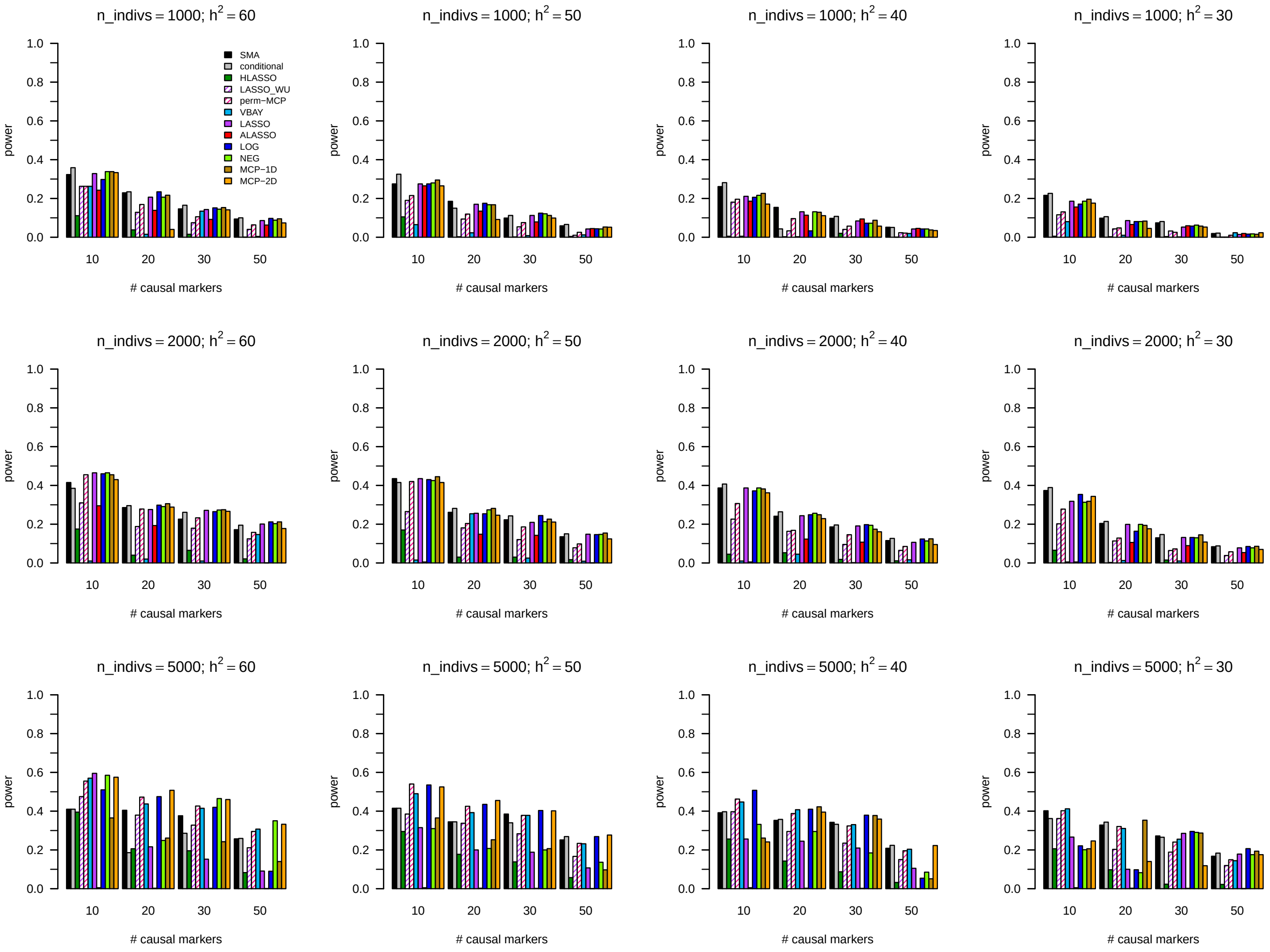

Supplement: Figure S4 — Power vs number of causal markers. Simulation results showing power for our PMR methods, current PMR methods, an approximate Bayesian method, single marker analysis and conditional regression methods at an FDR of 5% as a function of the number of susceptibility loci. Results are shown for a range of sample sizes and total heritabilities. (PDF) [file pcbi.1003101.s004.pdf]
