# Supplementary material for: PUMA: A Unified Framework for Penalized Multiple Regression Analysis of GWAS Data
Source: PLoS Comput Biol. 2013 Jun 27;9(6):e1003101. doi: 10.1371/journal.pcbi.1003101 (PMC3694815; doi:10.1371/journal.pcbi.1003101)

Figure S6: Power vs marginal heritability for 2000 samples. Simulation results showing power a sample size of 2000 for our PMR methods, current PMR methods, an approximate Bayesian method, single marker analysis and conditional regression methods at an FDR of 5% as a function of the marginal heritability of each causal marker as in Figure 3c in the main text. Results are shown for a range of total heritabilities and number of causal markers..

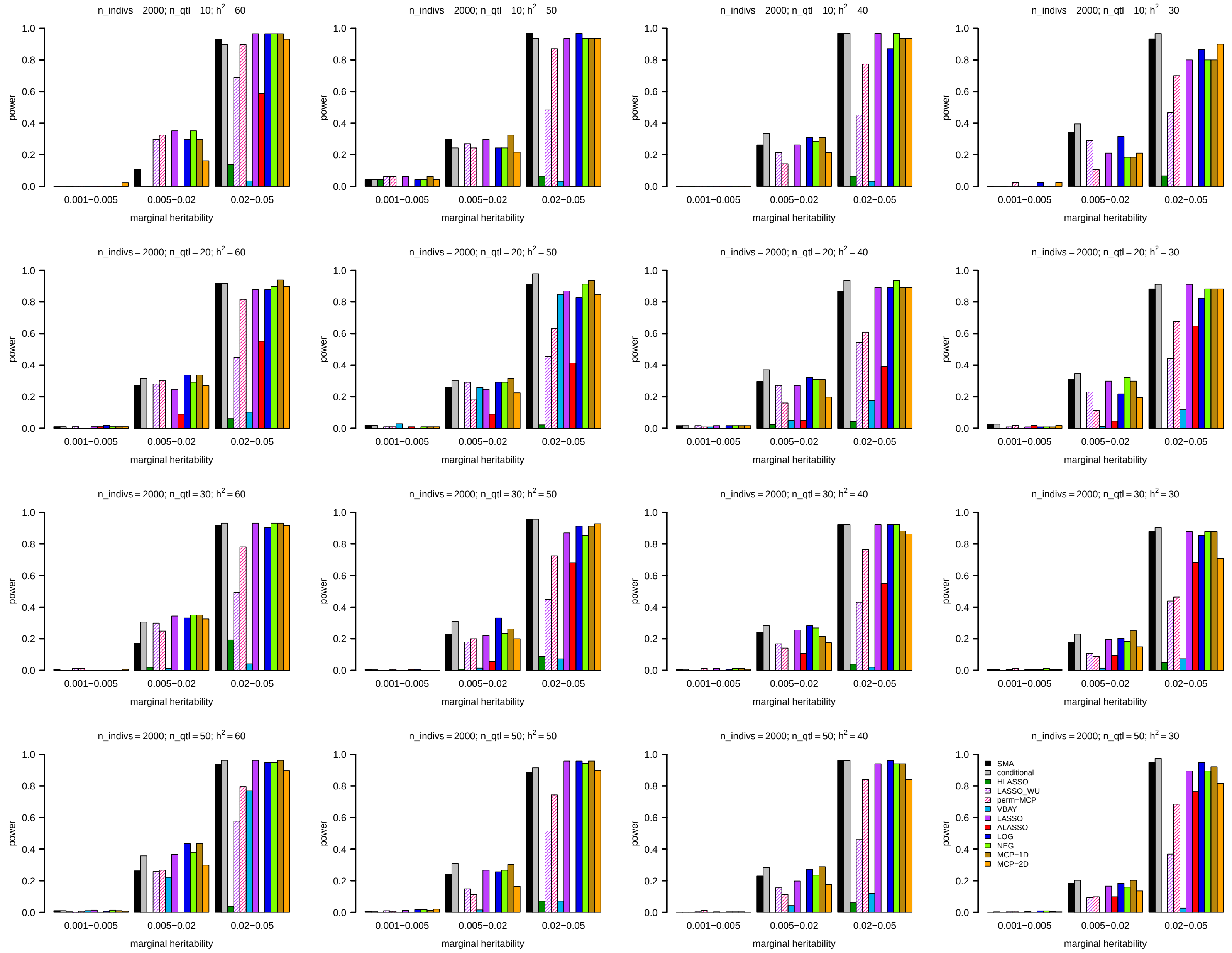

Supplement: Figure S6 — Power vs marginal heritability for 2000 samples. Simulation results showing power a sample size of 2000 for our PMR methods, current PMR methods, an approximate Bayesian method, single marker analysis and conditional regression methods at an FDR of 5% as a function of the marginal heritability of each causal marker as in Figure 3c in the main text. Results are shown for a range of total heritabilities and number of causal markers. (PDF) [file pcbi.1003101.s006.pdf]
