# Supplementary material for: PUMA: A Unified Framework for Penalized Multiple Regression Analysis of GWAS Data
Source: PLoS Comput Biol. 2013 Jun 27;9(6):e1003101. doi: 10.1371/journal.pcbi.1003101 (PMC3694815; doi:10.1371/journal.pcbi.1003101)

Figure S8: **Precision-Recall curves for 1000 samples.** Simulation results showing precision-recall curves for a sample size of 1000. Results are shown for a range of total heritabilities and number of causal markers. Solid colors for pML methods indicate results using our method for assessing significance in the presence of correlated markers, while dashes indicate the significance method of Wu et al. [2009] and perm-MCP [Ayers and Cordell, 2010]. This figures is analogous to Figure 3b in the main text.

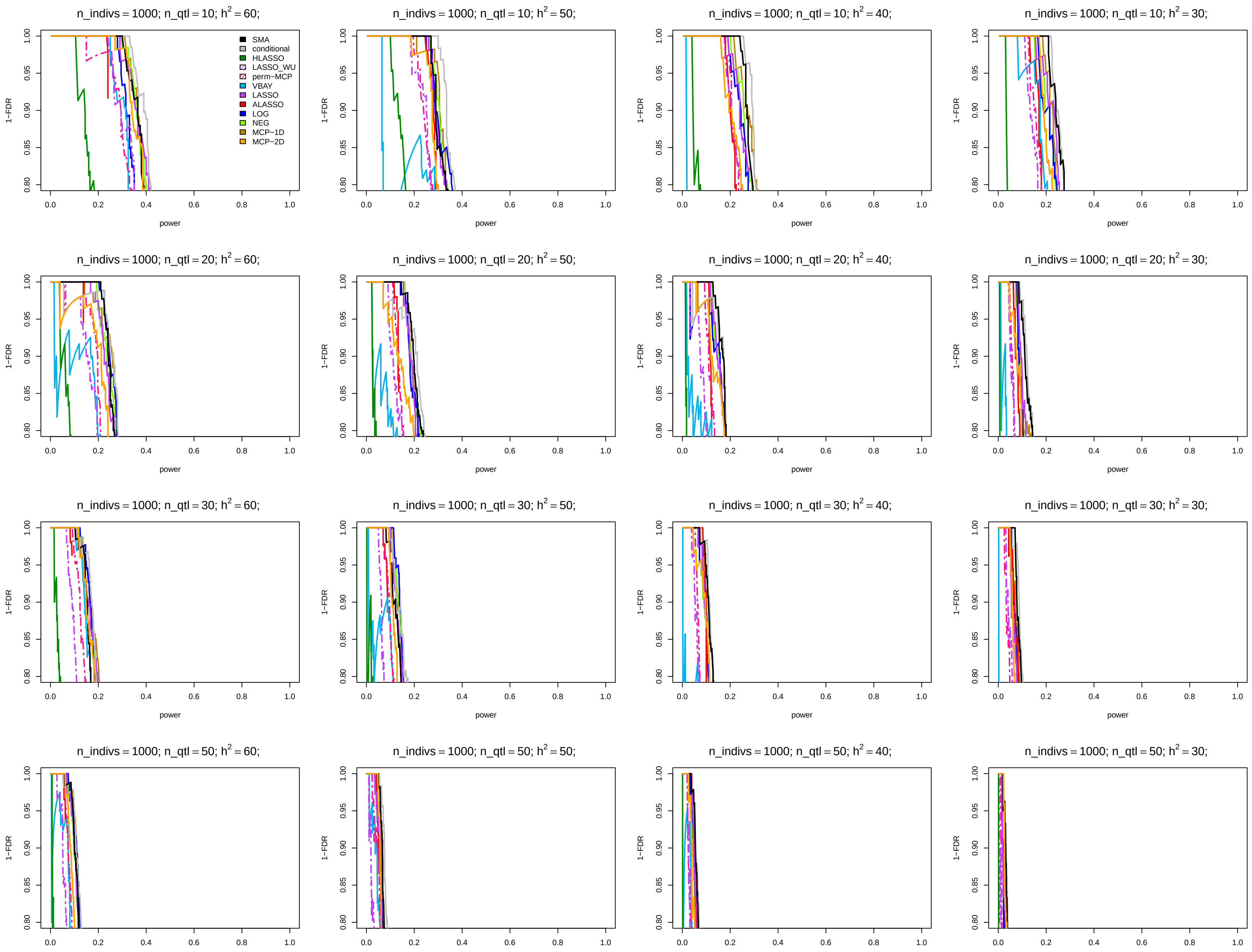

Supplement: Figure S8 — Precision-Recall curves for 1000 samples. Simulation results showing precision-recall curves for a sample size of 1000. Results are shown for a range of total heritabilities and number of causal markers. Solid colors for pML methods indicate results using our method for assessing significance in the presence of correlated markers, while dashes indicate the significance method of Wu, et al. [31] and perm-MCP [32]. This figures is analogous to Figure 3b in the main text. (PDF) [file pcbi.1003101.s008.pdf]
