# Supplementary material for: PUMA: A Unified Framework for Penalized Multiple Regression Analysis of GWAS Data
Source: PLoS Comput Biol. 2013 Jun 27;9(6):e1003101. doi: 10.1371/journal.pcbi.1003101 (PMC3694815; doi:10.1371/journal.pcbi.1003101)

Figure S11: Precision-Recall curves for perm-MCP for multiple values of eFPR and pre-screening p-value cutoff. Simulations for 5000 samples, 20 causal markers and heritability of 50% using eFPR values (1×10^{-3},1×10^{-4},1×10^{-5},1×10^{-6},1×10^{-7}) and pre-screening cutoff values (0.1, 0.01, 0.001) indicated in the legend. Results from single marker analysis and MCP-2D are shown for comparison.

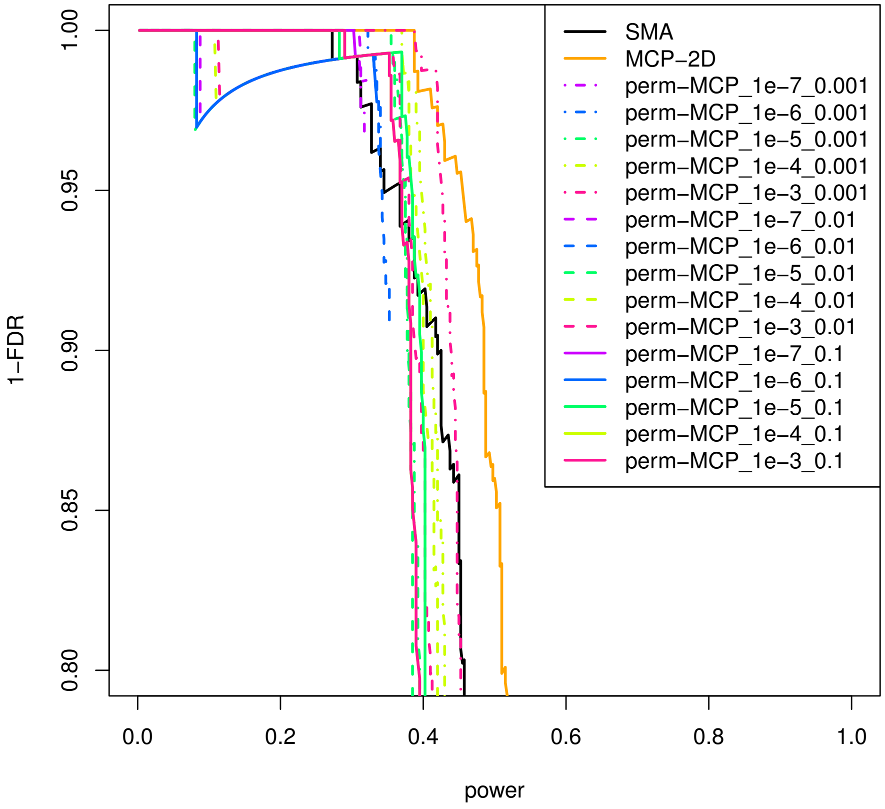

Supplement: Figure S11 — Precision-Recall curves for perm-MCP for multiple values of eFPR and pre-screening p-value cutoff. Simulations for 5000 samples, 20 causal markers and heritability of 50% using eFPR values (1, 1, 1, 1, 1) and pre-screening cutoff values (0.1, 0.01, 0.001) indicated in the legend. Results from single marker analysis and MCP-2D are shown for comparison. (PDF) [file pcbi.1003101.s011.pdf]
