# Supplementary material for: PUMA: A Unified Framework for Penalized Multiple Regression Analysis of GWAS Data
Source: PLoS Comput Biol. 2013 Jun 27;9(6):e1003101. doi: 10.1371/journal.pcbi.1003101 (PMC3694815; doi:10.1371/journal.pcbi.1003101)

Figure S12: Quantile-Quantile plots for each disease and method. Plots are shown for **a)** Crohn's disease, **b)** Rheumatoid arthritis and **c)** Type 1 diabetes. Results from a standard single marker analysis of each disease are shown in grey and are the same in all plots for a given disease. Results from including the subset of significantly associated markers identified by each pML method as covariates in a single marker analysis of remaining markers is shown in black, where the relevant method is indicated above each plot. Results from replacing the p-values from this latter analysis with p-values from the PMR method for the relevant markers with nonzero coefficients are shown in color. The genomic inflation values for are shown in the upper left of each plot. Note that the NEG method failed for the type 1 diabetes dataset, so no plot is shown.

(a) Crohn's disease

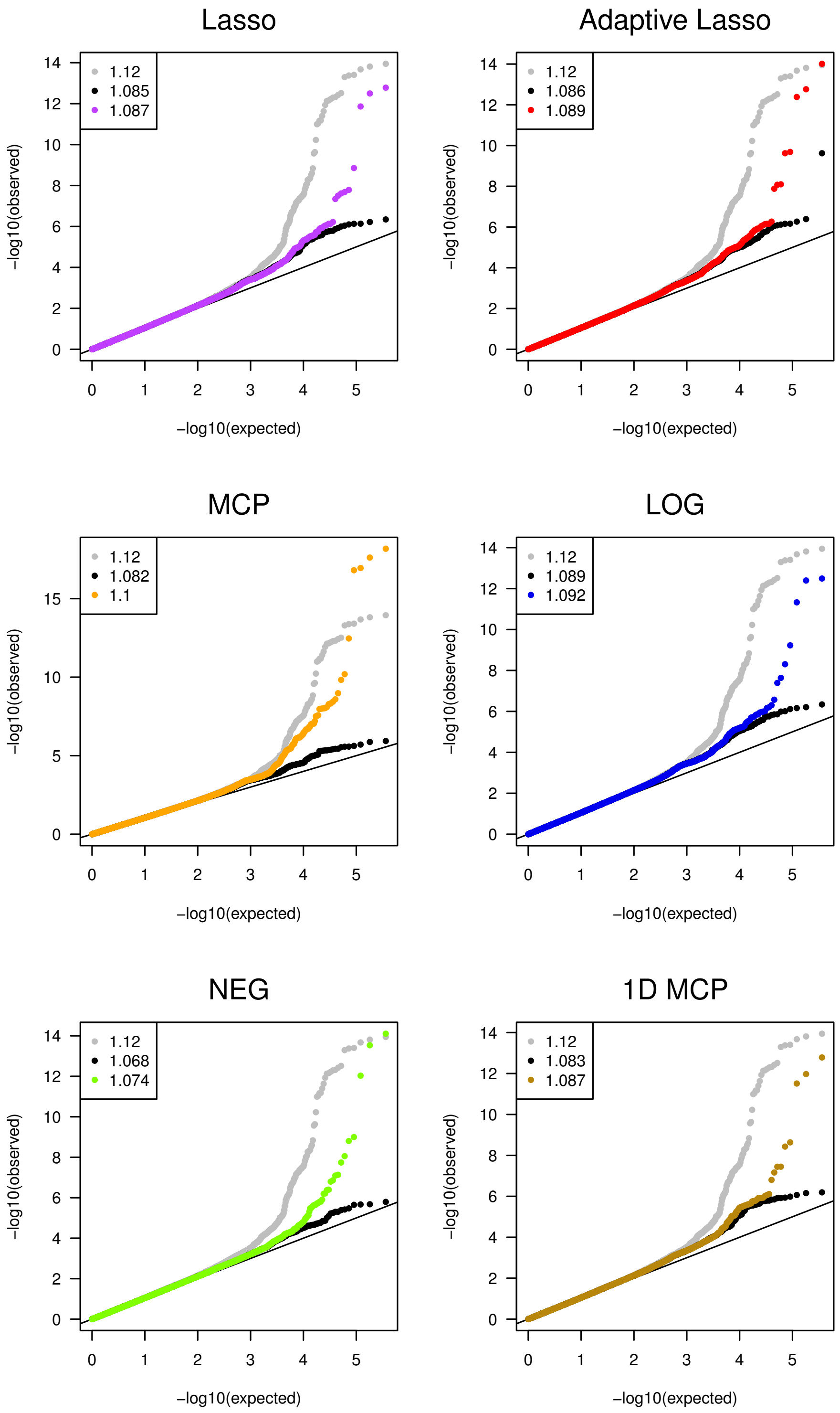

(b) Rheumatoid arthritis

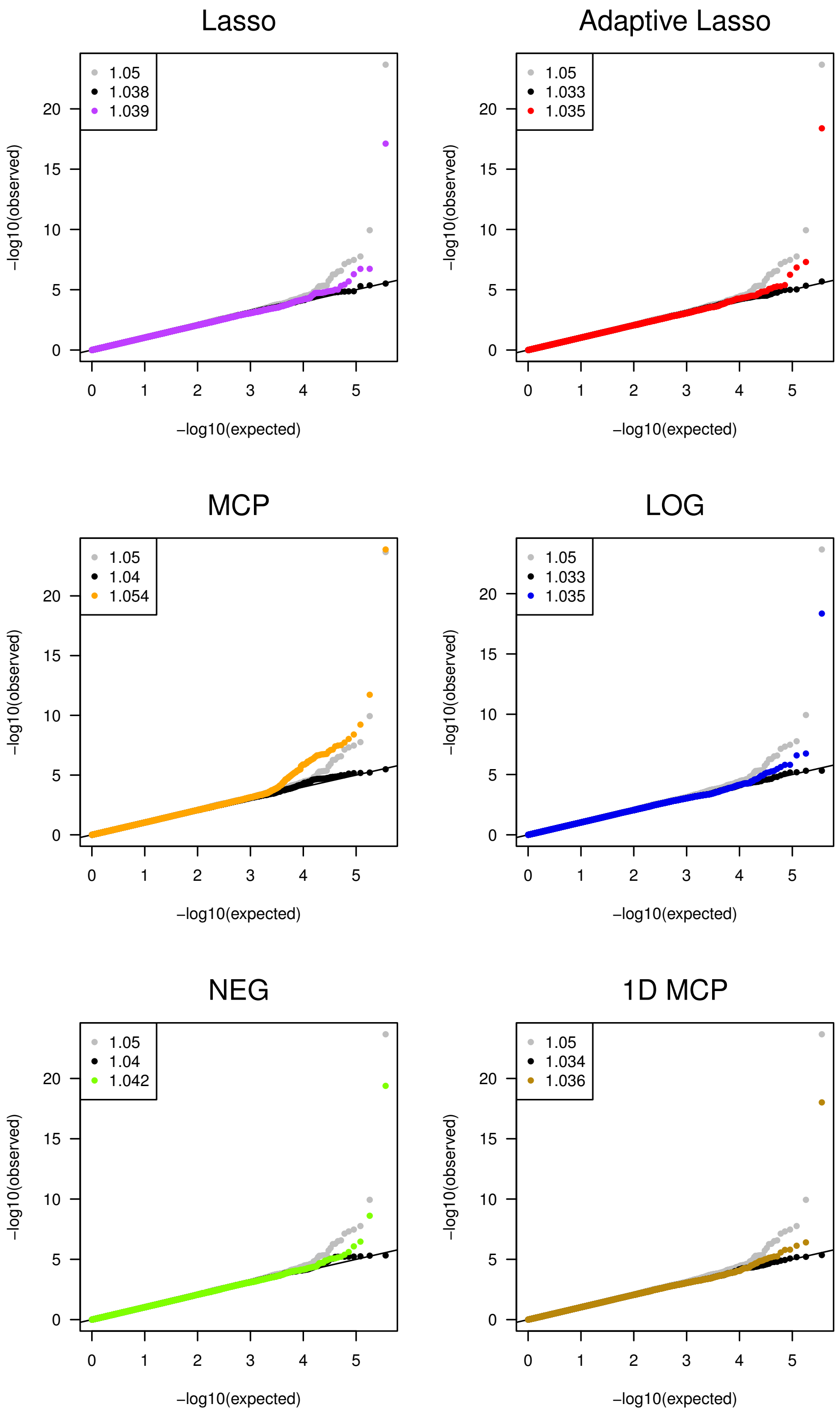

(c) Type 1 diabetes

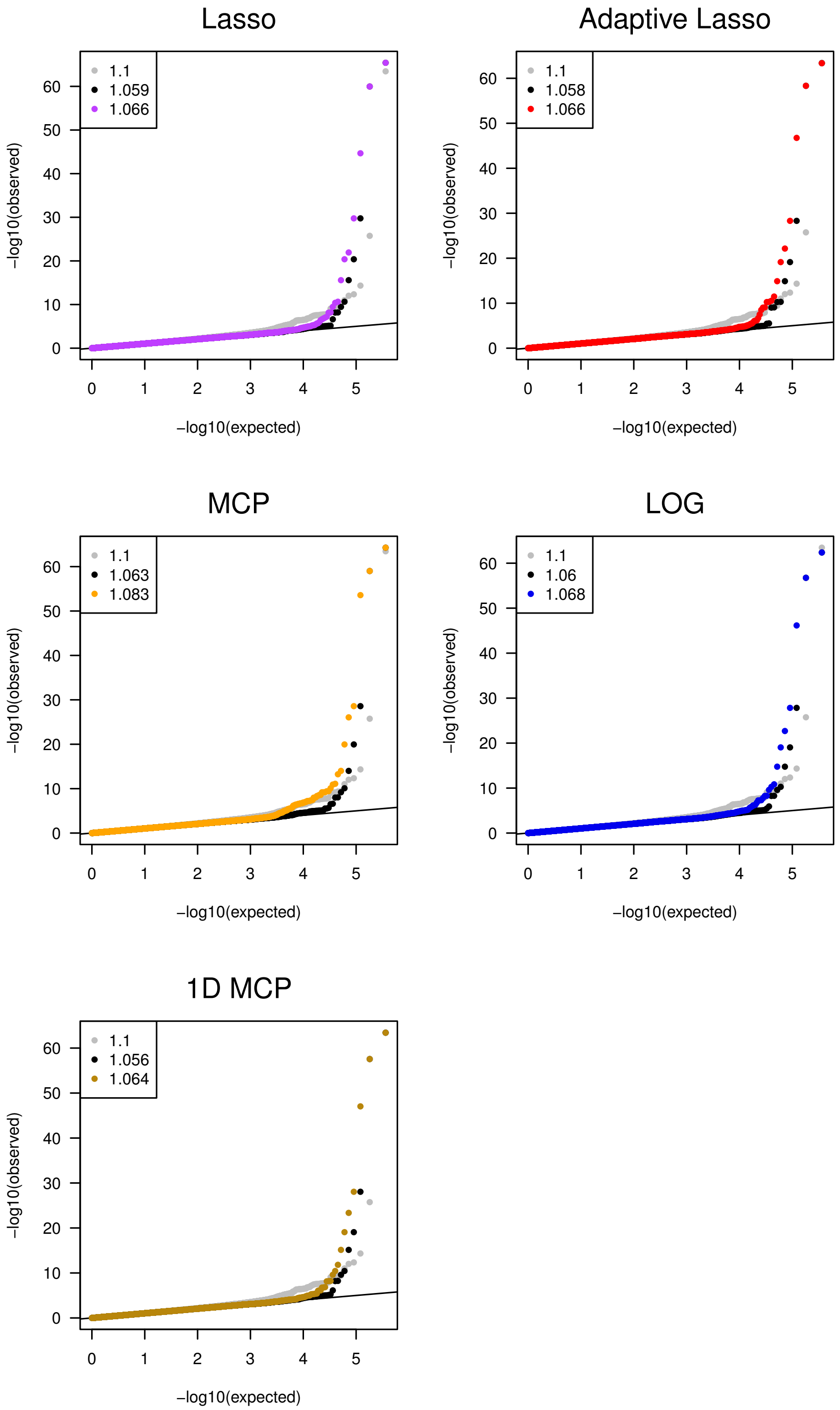

Supplement: Figure S12 — Quantile-Quantile plots for each disease and method. Plots are shown for a) Crohn's disease, b) Rheumatoid arthritis and c) Type 1 diabetes. Results from a standard single marker analysis of each disease are shown in grey and are the same in all plots for a given disease. Results from including the subset of significantly associated markers identified by each pML method as covariates in a single marker analysis of remaining markers is shown in black, where the relevant method is indicated above each plot. Results from replacing the p-values from this latter analysis with p-values from the PMR method for the relevant markers with nonzero coefficients are shown in color. The genomic inflation values for are shown in the upper left of each plot. Note that the NEG method failed for the type 1 diabetes dataset, so no plot is shown. (PDF) [file pcbi.1003101.s012.pdf]
