# Supplementary material for: PUMA: A Unified Framework for Penalized Multiple Regression Analysis of GWAS Data
Source: PLoS Comput Biol. 2013 Jun 27;9(6):e1003101. doi: 10.1371/journal.pcbi.1003101 (PMC3694815; doi:10.1371/journal.pcbi.1003101)

Figure S13: Manhattan plots showing results of single marker analysis for **a**) Crohn's disease, **b**) Rheumatoid arthritis, and **c**) Type 1 diabetes datasets from our re-analysis. Shown are $-\log_{10}$ p-values where large values are truncated at 20. Markers with $-\log_{10}$ p-values > 6 are colored green.

(a) Crohn's disease

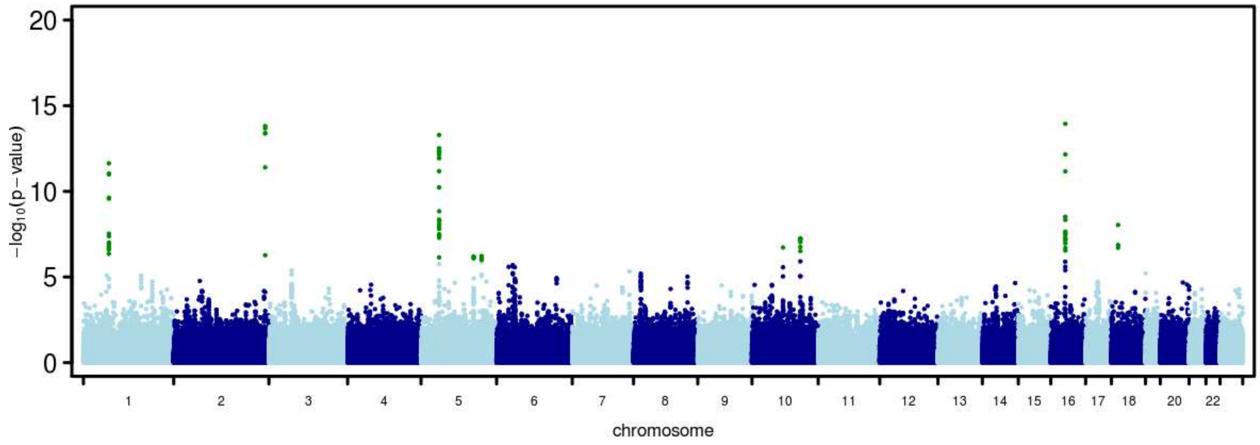

(b) Rheumatoid arthritis

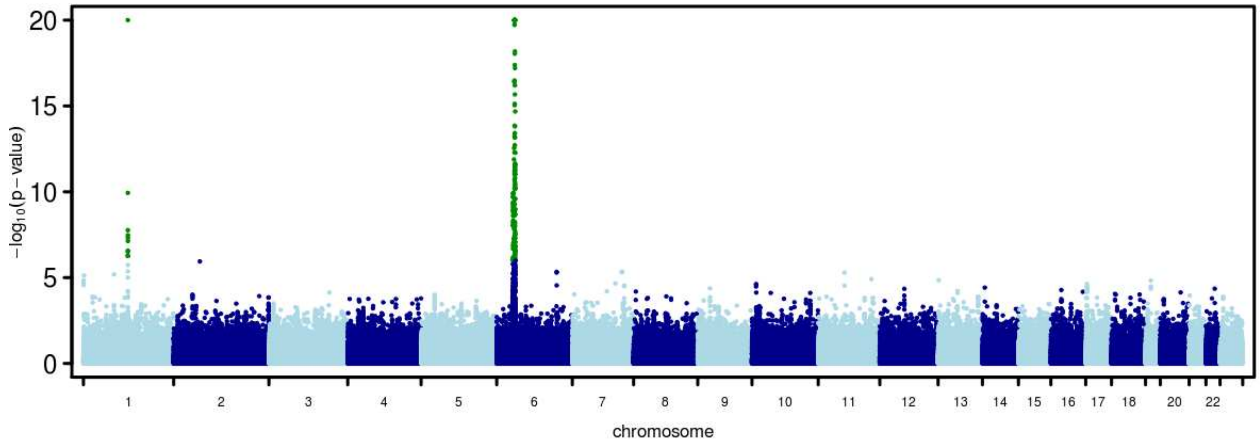

(c) Type 1 diabetes

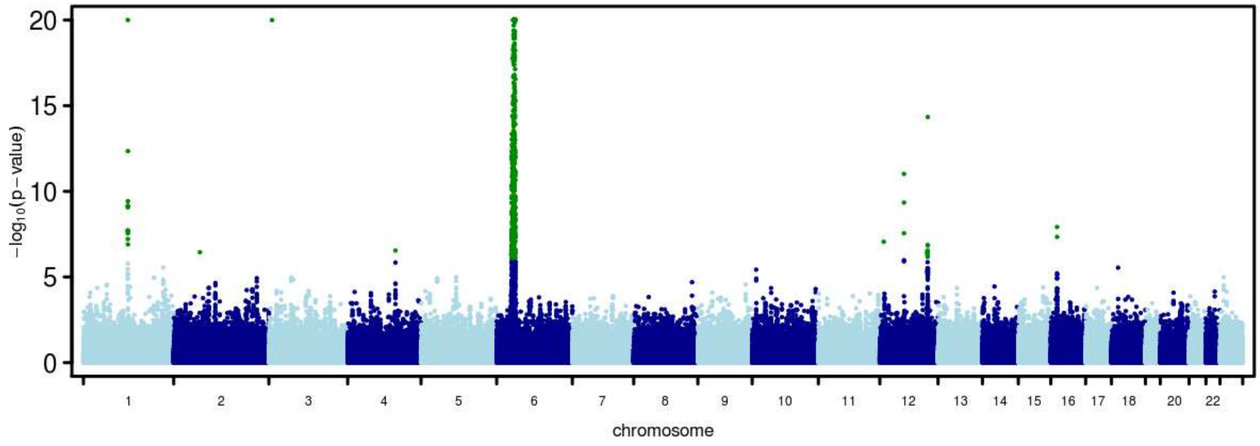

Supplement: Figure S13 — Manhattan plots of single marker analysis for three disease datasets. Manhattan plots showing results of single marker analysis for a) Crohn's disease, b) Rheumatoid arthritis, and c) Type 1 diabetes datasets from our re-analysis. Shown are p-values where large values are truncated at 20. Markers with p-values are colored green. (PDF) [file pcbi.1003101.s013.pdf]
