# Supplementary material for: PUMA: A Unified Framework for Penalized Multiple Regression Analysis of GWAS Data
Source: PLoS Comput Biol. 2013 Jun 27;9(6):e1003101. doi: 10.1371/journal.pcbi.1003101 (PMC3694815; doi:10.1371/journal.pcbi.1003101)
Supplement: Figure S14 — Genome-wide plots of hits found by each method. Genome-wide plot of associations identified by analyzing the WTCCC data for a) Crohn's disease, b) rheumatoid arthritis and c) type 1 diabetes using PMR methods, conditional regression, and single marker analysis. External associations from independent datasets (which do not include WTCCC data) and non-independent datasets (which include WTCCC data) of the same disease are indicated with pink boxes and diamonds, respectively. Markers that are considered associations only when the p-value threshold for the single marker analysis is relaxed to match the same number of associations (with hits in the MHC region excluded) as the union of all PMR methods are indicated with black circles. Arrows indicate novel associations that are biologically interpretable. (PDF) [file pcbi.1003101.s014.pdf]

**Figure S14:** Genome-wide plot of associations identified by analyzing the WTCCC data for **a)** Crohn's disease, **b)** rheumatoid arthritis and **c)** type 1 diabetes using PMR methods, conditional regression, and single marker analysis. External associations from independent datasets (which do not include WTCCC data) and non-independent datasets (which include WTCCC data) of the same disease are indicated with pink boxes and diamonds, respectively. Markers that are considered associations only when the p-value threshold for the single marker analysis is relaxed to match the same number of associations (with hits in the MHC region excluded) as the union of all PMR methods are indicated with black circles. Arrows indicate novel associations that are biologically interpretable.

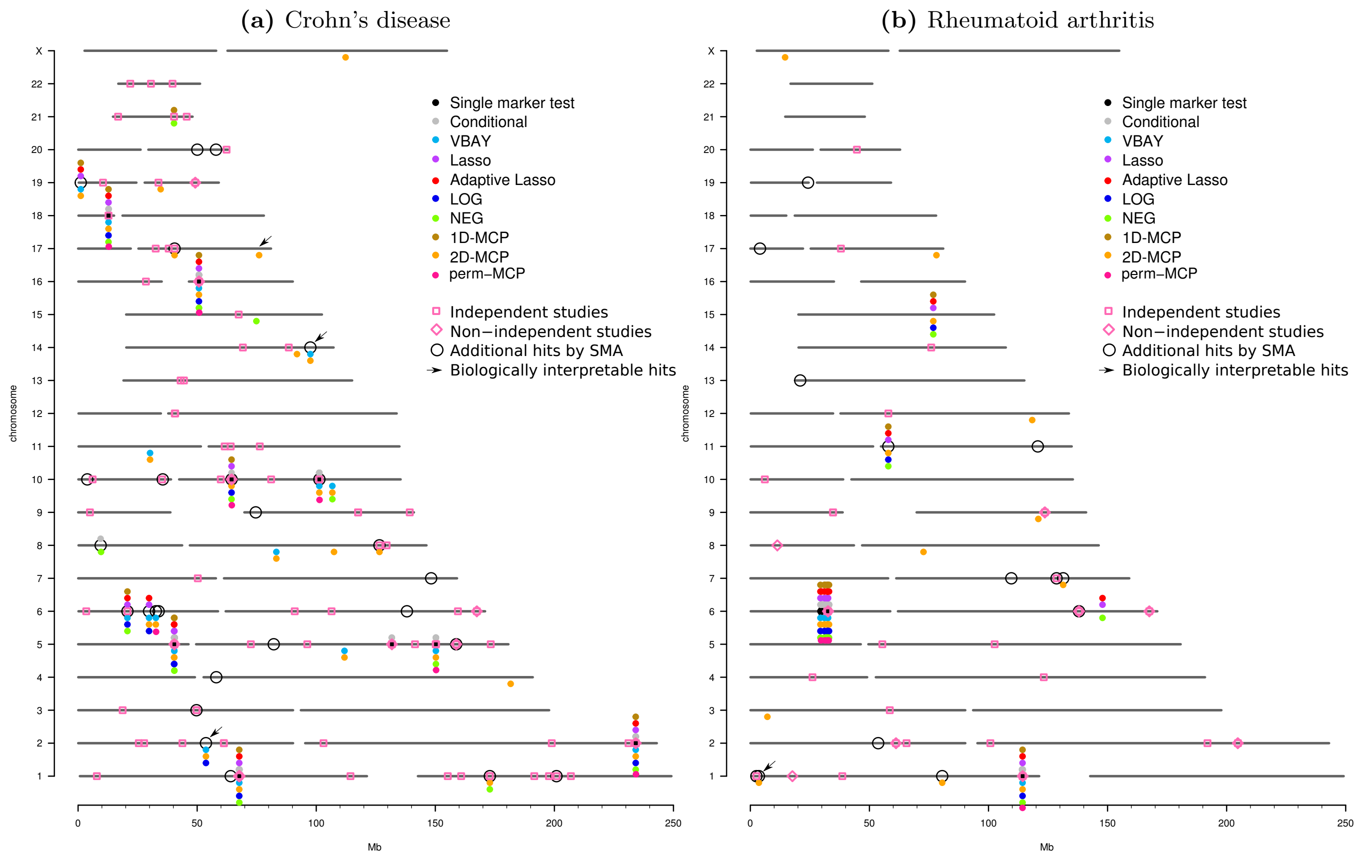

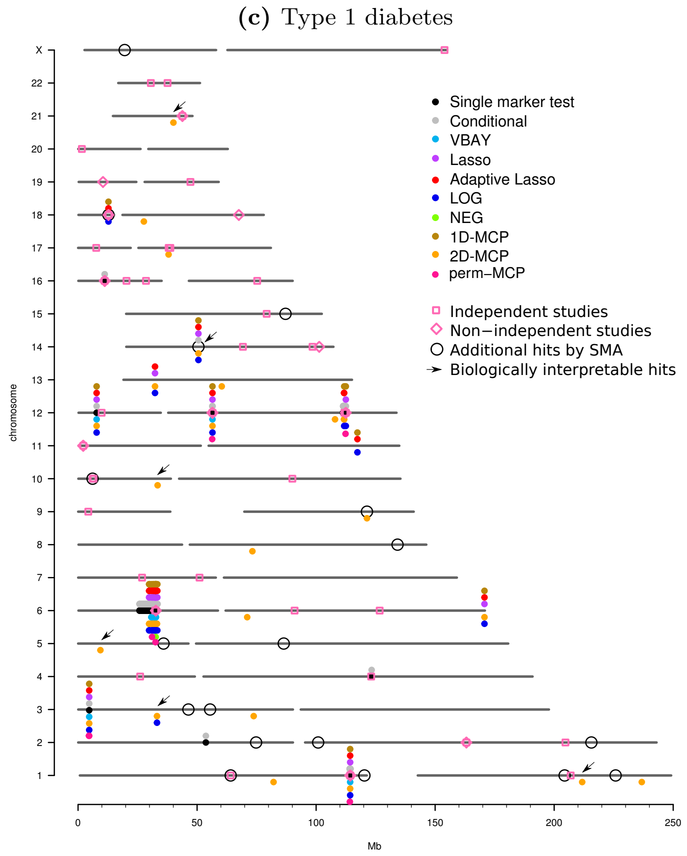
(c) Type 1 diabetes
Single marker test
Conditional
VBAY
Lasso
Adaptive Lasso
LOG
NEG
1D-MCP
2D-MCP
perm−MCP
Independent studies
Non−independent studies
Additional hits by SMA
Biologically interpretable hits
chromosome
X
22
21
20
19
18
17
16
15
14
13
12
11
10
9
8
7
6
5
4
3
2
1
0
50
100
150
200
250
Mb
