# Supplementary material for: PUMA: A Unified Framework for Penalized Multiple Regression Analysis of GWAS Data
Source: PLoS Comput Biol. 2013 Jun 27;9(6):e1003101. doi: 10.1371/journal.pcbi.1003101 (PMC3694815; doi:10.1371/journal.pcbi.1003101)

Figure S15: Local manhattan plots of hits replicated from an independent study of Crohn's disease

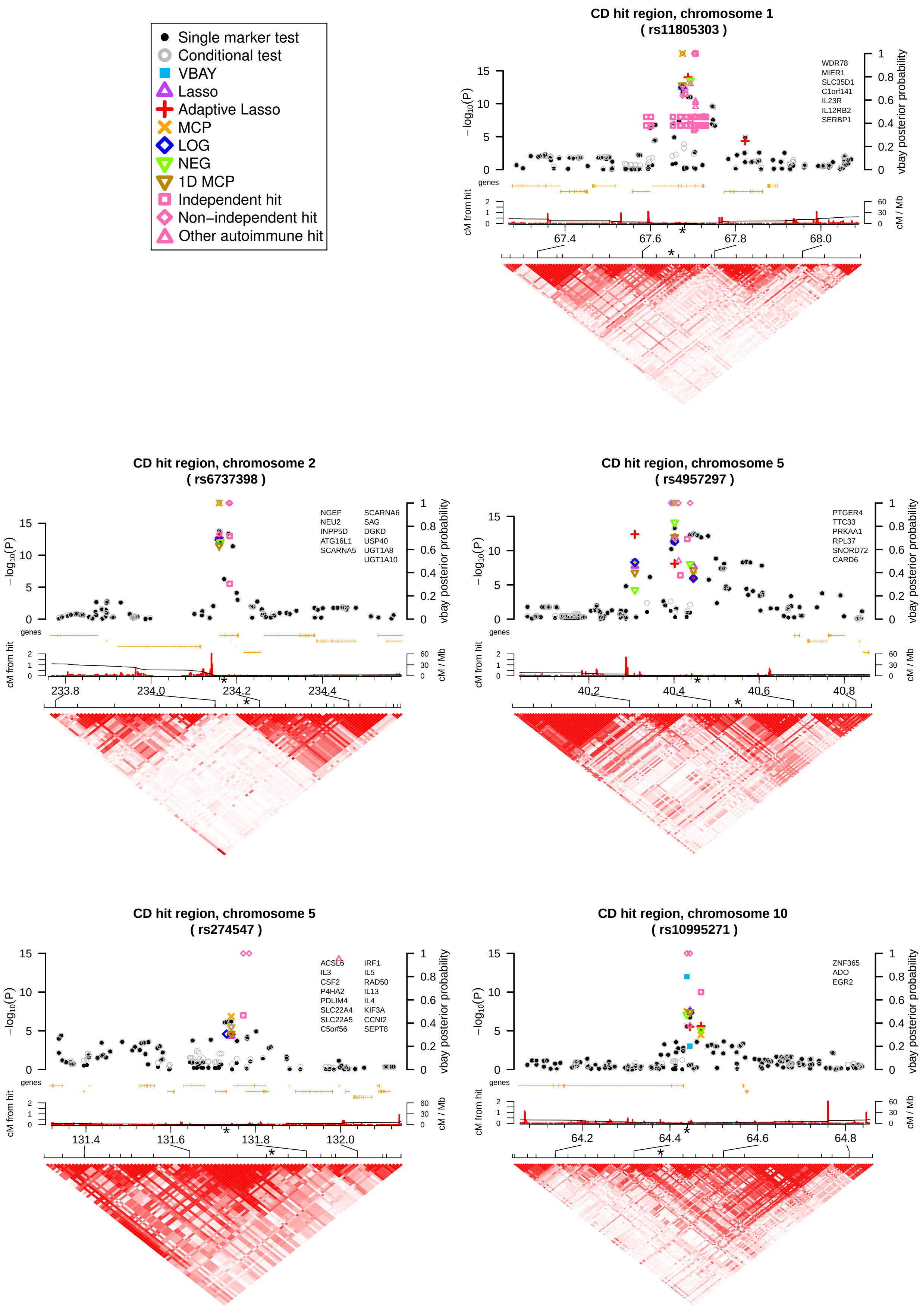

CD hit region, chromosome 16
(rs3135499)

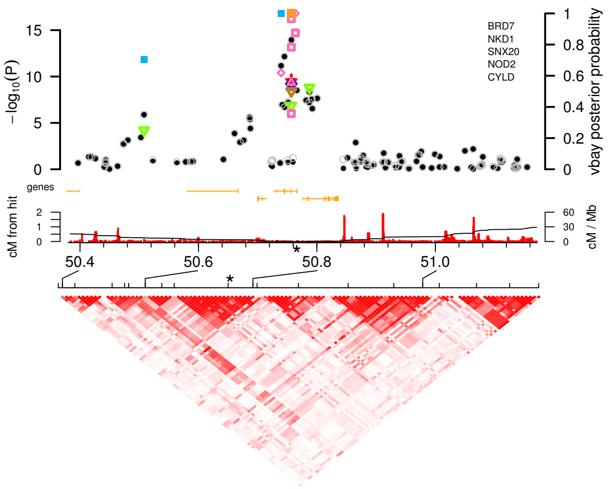

Supplement: Figure S15 — Local manhattan plots of hits replicated from an independent study of Crohn's disease. (PDF) [file pcbi.1003101.s015.pdf]
