# Supplementary figures and images for: PUMA: A Unified Framework for Penalized Multiple Regression Analysis of GWAS Data
Source: PLoS Comput Biol. 2013 Jun 27;9(6):e1003101. doi: 10.1371/journal.pcbi.1003101 (PMC3694815; doi:10.1371/journal.pcbi.1003101)

**Figure S17:** Local manhattan plots of hits replicated from an independent study of type 1 diabetes

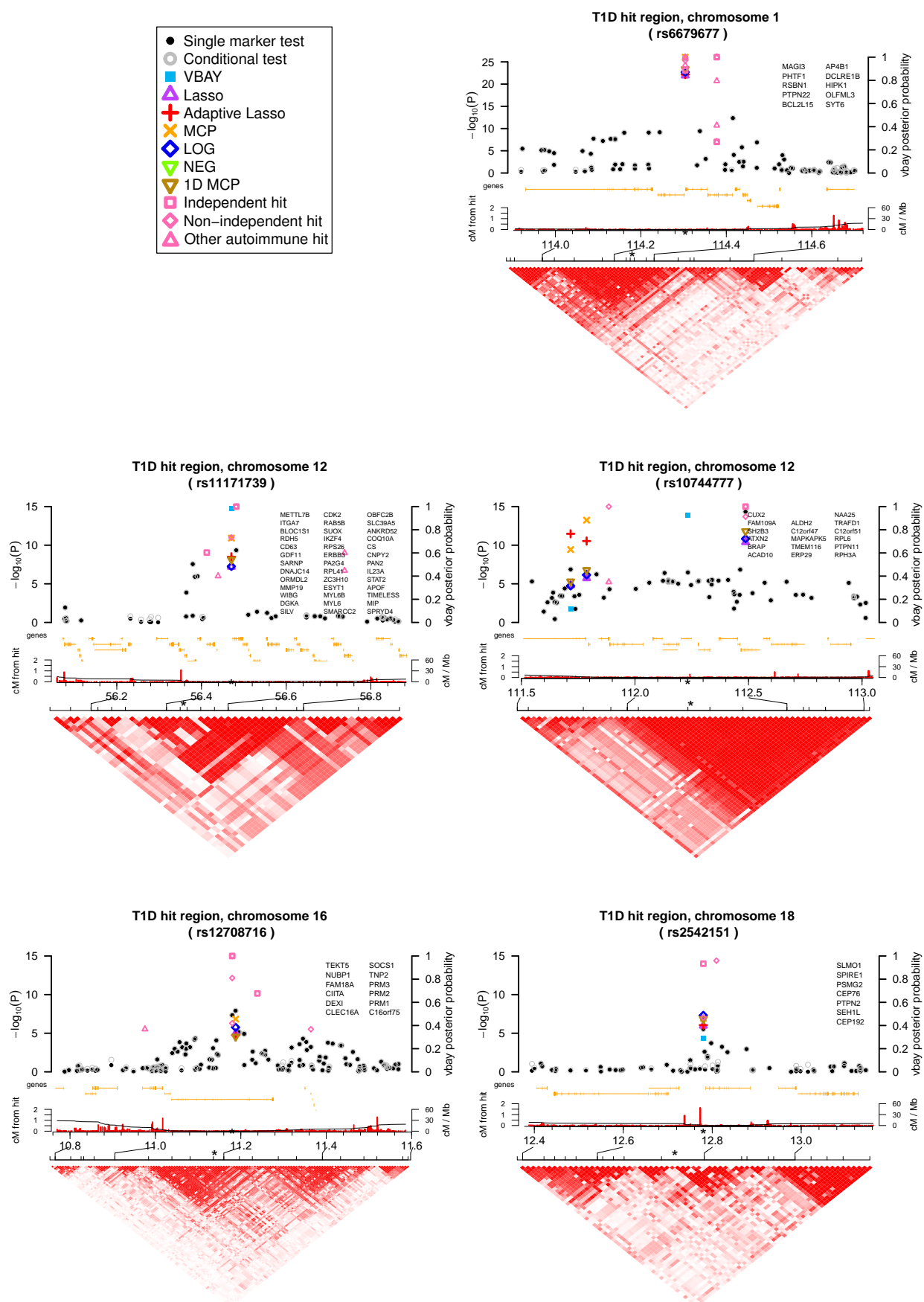

Supplement: Figure S17 — Local manhattan plots of hits replicated from an independent study of type 1 diabetes. (PDF) [file pcbi.1003101.s017.pdf]

**Figure S20:** Local manhattan plots of biologically relevant hits for Crohn's disease

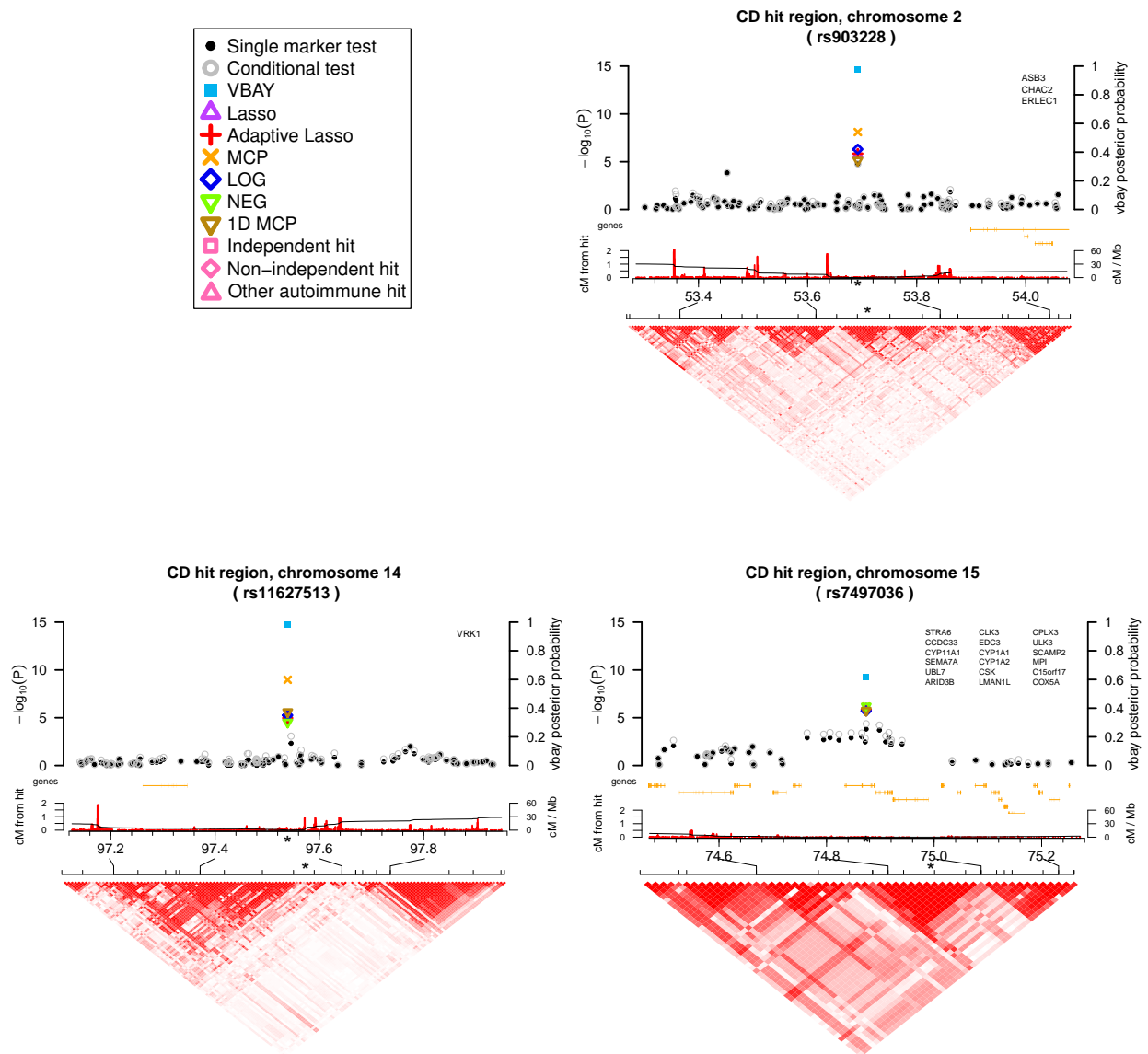

Supplement: Figure S20 — Local manhattan plots of biologically relevant hits for Crohn's disease. (PDF) [file pcbi.1003101.s020.pdf]

**Figure S21:** Local manhattan plots of biologically relevant hits for rheumatoid arthritis

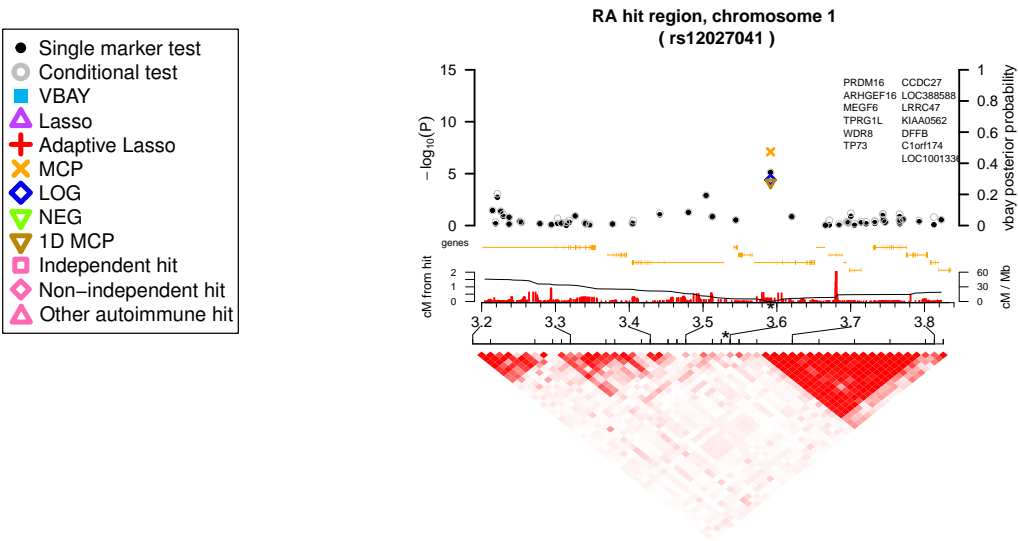

Supplement: Figure S21 — Local manhattan plots of biologically relevant hits for rheumatoid arthritis. (PDF) [file pcbi.1003101.s021.pdf]
