# Supplementary material for: PUMA: A Unified Framework for Penalized Multiple Regression Analysis of GWAS Data
Source: PLoS Comput Biol. 2013 Jun 27;9(6):e1003101. doi: 10.1371/journal.pcbi.1003101 (PMC3694815; doi:10.1371/journal.pcbi.1003101)

Figure S18: Local manhattan plots hits replicated from a non-independent study of Crohn's disease

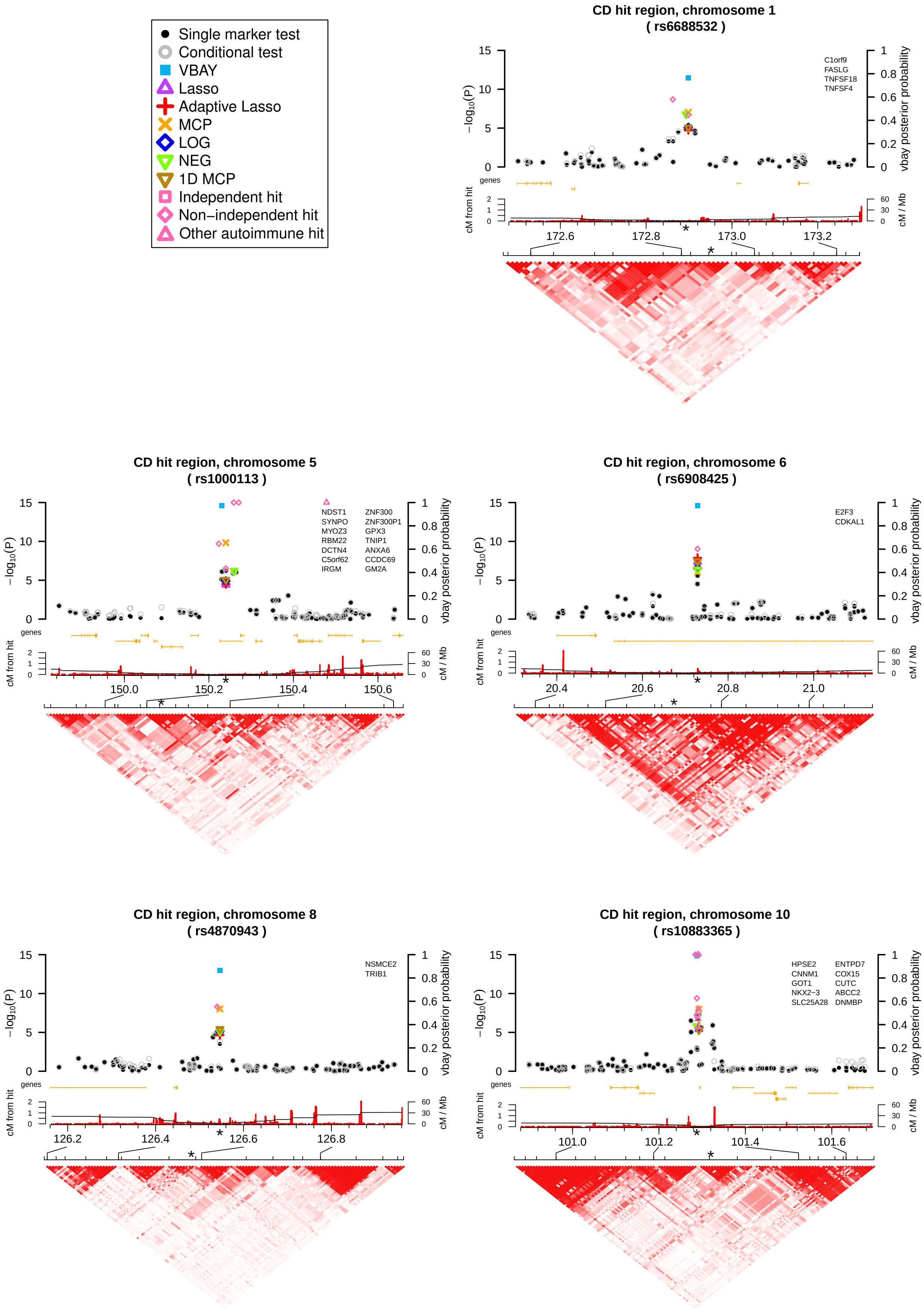

CD hit region, chromosome 17
(rs744166)

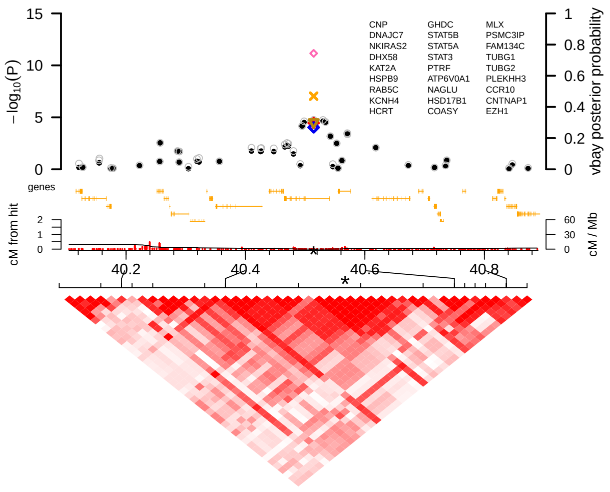

CD hit region, chromosome 18
(rs16939895)

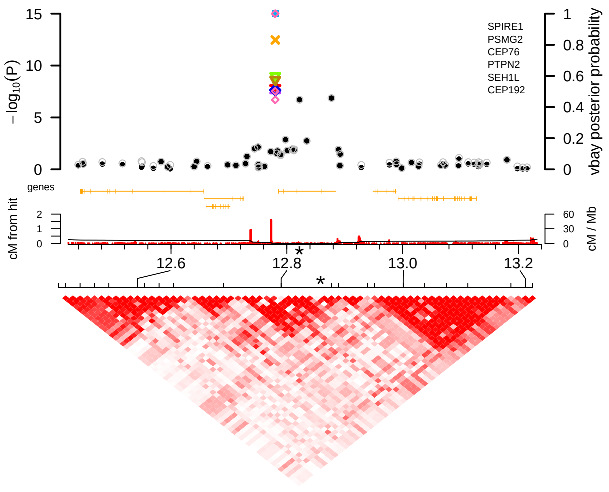

CD hit region, chromosome 21
(rs2836754)

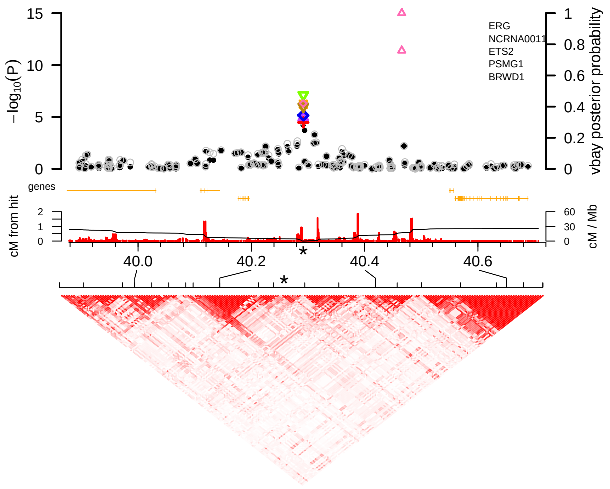

Supplement: Figure S18 — Local manhattan plots hits replicated from a non-independent study of Crohn's disease. (PDF) [file pcbi.1003101.s018.pdf]
