# Supplementary material for: PUMA: A Unified Framework for Penalized Multiple Regression Analysis of GWAS Data
Source: PLoS Comput Biol. 2013 Jun 27;9(6):e1003101. doi: 10.1371/journal.pcbi.1003101 (PMC3694815; doi:10.1371/journal.pcbi.1003101)

Figure S19: Local manhattan plots of hits replicated from a non-independent study of type 1 diabetes

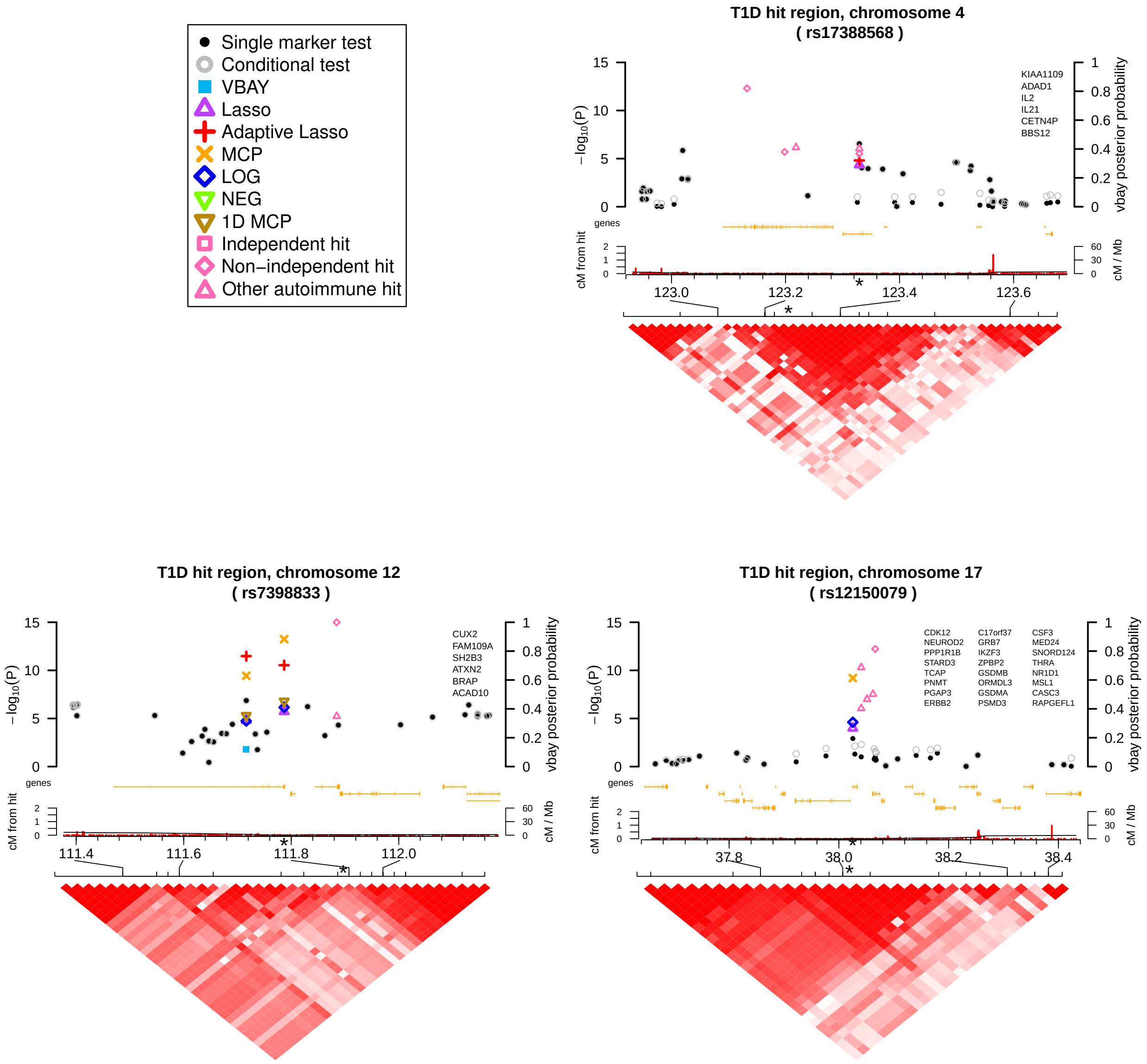

Supplement: Figure S19 — Local manhattan plots of hits replicated from a non-independent study of type 1 diabetes. (PDF) [file pcbi.1003101.s019.pdf]
