# Supplementary material for: PUMA: A Unified Framework for Penalized Multiple Regression Analysis of GWAS Data
Source: PLoS Comput Biol. 2013 Jun 27;9(6):e1003101. doi: 10.1371/journal.pcbi.1003101 (PMC3694815; doi:10.1371/journal.pcbi.1003101)
Supplement: Figure S22 — Local manhattan plots of biologically relevant hits for type 1 diabetes. (PDF) [file pcbi.1003101.s022.pdf]

Figure S22: Local manhattan plots of biologically relevant hits for type 1 diabetes

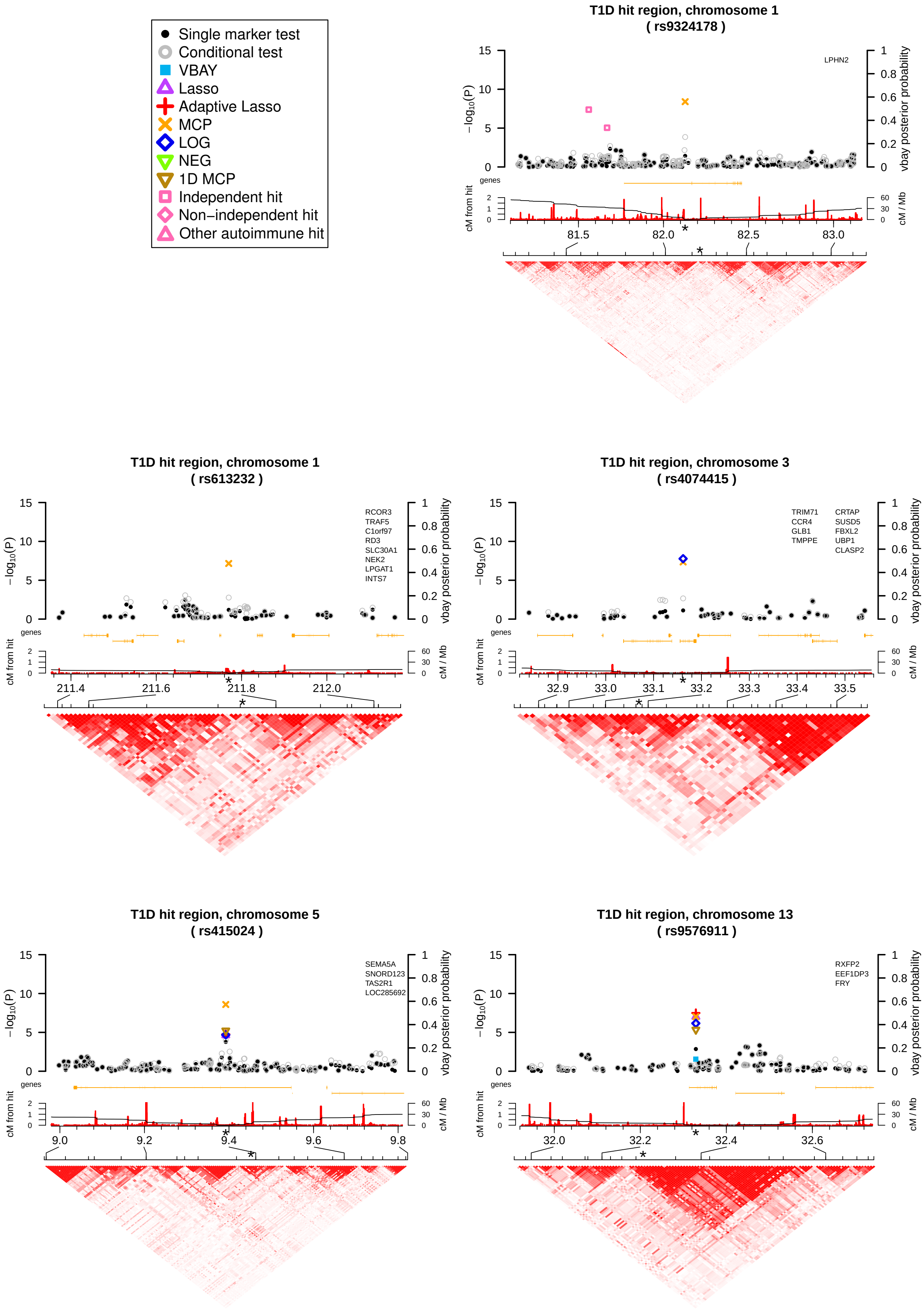

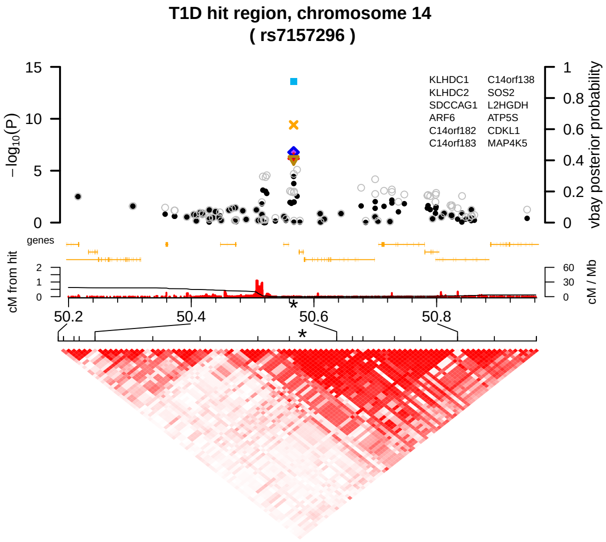
T1D hit region, chromosome 14
(rs7157296)
−log10(P)
vbay posterior probability
KLHDC1
KLHDC2
SDCCAG1
ARF6
C14orf182
C14orf183
C14orf138
SOS2
L2HGDH
ATP5S
CDKL1
MAP4K5
genes
cM from hit
cM / Mb
50.2
50.4
50.6
50.8

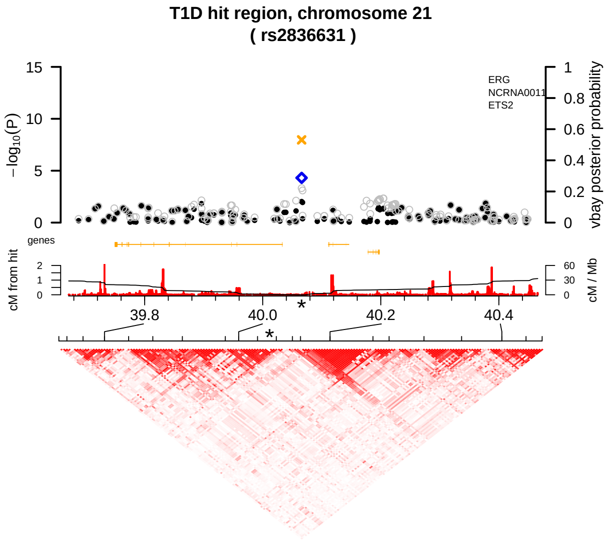
T1D hit region, chromosome 21
(rs2836631)
−log10(P)
vbay posterior probability
ERG
NCRNA0011
ETS2
genes
cM from hit
cM / Mb
39.8
40.0
40.2
40.4
