# Supplementary material for: PUMA: A Unified Framework for Penalized Multiple Regression Analysis of GWAS Data
Source: PLoS Comput Biol. 2013 Jun 27;9(6):e1003101. doi: 10.1371/journal.pcbi.1003101 (PMC3694815; doi:10.1371/journal.pcbi.1003101)
Supplement: Table S1 — Concordance of PMR hits with single marker analysis. Number of regions identified by a single marker analysis with a p-value1 and the number of these regions that are recapitulated by each other method. (PDF) [file pcbi.1003101.s023.pdf]

Table S1: Number of regions identified by a single marker analysis with a p-value $< 1 \times 10^{-6}$ and the number of these regions that are recapitulated by each other method.

	Method									
disease	SMA	Conditional	VBAY	Lasso	Adaptive Lasso	2D-MCP	LOG	NEG	1D-MCP	perm-MCP
CD	9	9	7	6	5	8	6	7	6	6
RA	1	1	1	1	1	1	1	1	1	1
T1D	9	9	3	4	4	4	4	0	4	4
Total	19	19	11	11	10	13	11	8	11	11
