# Supplementary material for: PUMA: A Unified Framework for Penalized Multiple Regression Analysis of GWAS Data
Source: PLoS Comput Biol. 2013 Jun 27;9(6):e1003101. doi: 10.1371/journal.pcbi.1003101 (PMC3694815; doi:10.1371/journal.pcbi.1003101)
Supplement: Table S2 — Regions identified only by single marker analysis. Regions identified by single marker analysis with a p-value1. Of the regions missed by PMR methods, only one association passes the Bonferroni cutoff of = 1.38, although 5 of these have been replicated: rs6596075 [4], [116], [117], rs17388568 [6], rs10807124 [6], [61], [118], rs4766517 [6], [61], [118], rs12924729 [6], [61], [118], [119]. (PDF) [file pcbi.1003101.s024.pdf]

Table S2: Regions identified by single marker analysis with a p-value $< 1 \times 10^{-6}$. Of the regions missed by PMR methods, only one association passes the Bonferroni cutoff of $0.05/360,000 = 1.38 \times 10^{-7}$, although 5 of these have been replicated: rs6596075 [Barrett et al., 2008, Franke et al., 2010, McGovern et al., 2010], rs17388568 [Barrett et al., 2009], rs10807124 [Barrett et al., 2009, Cooper et al., 2008, Hakonarson et al., 2007], rs4766517 [Barrett et al., 2009, Cooper et al., 2008, Hakonarson et al., 2007], rs12924729 [Barrett et al., 2009, Cooper et al., 2008, Hakonarson et al., 2007, Todd et al., 2007].

				Method									
disease	SNP	chromosome	position	SMA	Conditional	VBAY	Lasso	Adaptive Lasso	2D-MCP	LOG	NEG	1D-MCP	perm-MCP
CD	rs11805303	1p31.3	67,675,515	2.34×10^{-12}	2.34×10^{-12}	1	3.18×10^{-13}	9.77×10^{-15}	2.43×10^{-18}	3.23×10^{-13}	2.92×10^{-14}	1.64×10^{-13}	-
CD	rs10210302	2q37.1	234,158,838	1.54×10^{-14}	1.54×10^{-14}	1	1.66×10^{-13}	1.73×10^{-13}	6.69×10^{-19}	4.04×10^{-13}	9.32×10^{-13}	3.09×10^{-12}	1.19×10^{-13}
CD	rs17234657	5p13.1	40,401,508	5.09×10^{-14}	5.09×10^{-14}	1	1.38×10^{-12}	4.15×10^{-13}	1.13×10^{-17}	4.71×10^{-12}	7.84×10^{-15}	1.07×10^{-12}	6.10×10^{-06}
CD	rs6596075	5q31.1	131,742,227	6.37×10^{-07}	6.37×10^{-07}	0.064	4.4×10^{-05}	2.95×10^{-05}	1.42×10^{-07}	2.58×10^{-05}	3.29×10^{-06}	2.3×10^{-05}	-
CD	rs1000113	5q33.1	150,240,075	5.99×10^{-07}	5.99×10^{-07}	0.973	3.69×10^{-05}	3.11×10^{-05}	1.49×10^{-10}	1.21×10^{-05}	6.14×10^{-07}	9.02×10^{-06}	5.04×10^{-07}
CD	rs10761659	10q21.2	64,445,563	1.9×10^{-07}	1.9×10^{-07}	0.8	3.13×10^{-08}	2.96×10^{-06}	3.11×10^{-08}	4.07×10^{-08}	8.03×10^{-08}	3.58×10^{-08}	7.41×10^{-08}
CD	rs10883371	10q24.2	101,292,454	5.55×10^{-08}	5.55×10^{-08}	0.99	3.78×10^{-06}	1.87×10^{-06}	1.03×10^{-08}	2.85×10^{-06}	1.57×10^{-06}	4.83×10^{-06}	2.84×10^{-07}
CD	rs2076756	16q12.1	50,756,880	1.14×10^{-14}	1.14×10^{-14}	1	1.4×10^{-09}	2.06×10^{-10}	1.56×10^{-17}	5.95×10^{-10}	1.58×10^{-09}	3.72×10^{-09}	8.93×10^{-09}
CD	rs2542151	18p11.21	12,779,946	9.2×10^{-09}	9.2×10^{-09}	1	2.44×10^{-08}	8.41×10^{-09}	3.45×10^{-13}	2.32×10^{-08}	1×10^{-09}	2.32×10^{-09}	7.56×10^{-08}
RA	rs6679677	1p13.2	114,303,807	2.16×10^{-24}	2.16×10^{-24}	1	7.64×10^{-18}	4.1×10^{-19}	1.31×10^{-24}	4.48×10^{-19}	4.11×10^{-20}	9.63×10^{-19}	7.53×10^{-20}
T1D	rs6679677	1p13.2	114,303,807	1.8×10^{-26}	1.8×10^{-26}	1	1.17×10^{-22}	7.17×10^{-23}	8.74×10^{-27}	2.04×10^{-23}	-	4.35×10^{-24}	2.25×10^{-20}
T1D	rs903228	2p16.2	53,692,048	3.59×10^{-07}	3.59×10^{-07}	0.000 398	-	-	-	-	-	-	-
T1D	rs17388568	4q27	123,329,361	2.87×10^{-07}	2.87×10^{-07}	0.002 93	4.33×10^{-05}	1.54×10^{-05}	-	-	-	-	-
T1D	rs10807124	6p21.32	33,404,063	2.07×10^{-09}	2.07×10^{-09}	7.02×10^{-05}	-	-	-	-	-	-	-
T1D	rs2110221	12p13.31	7,794,773	8.75×10^{-08}	8.75×10^{-08}	0.987	2.61×10^{-08}	2.19×10^{-08}	2.44×10^{-09}	4.49×10^{-08}	-	1.12×10^{-08}	-
T1D	rs11171739	12q13.2	56,470,624	9.67×10^{-12}	9.67×10^{-12}	0.983	6.31×10^{-08}	3.18×10^{-09}	1.2×10^{-11}	5.52×10^{-08}	-	7.2×10^{-09}	6.25×10^{-07}
T1D	rs4766517	12q24.11	111,359,711	2.87×10^{-07}	2.87×10^{-07}	0.000 433	-	-	-	-	-	-	-
T1D	rs17696736	12q24.13	112,486,817	4.62×10^{-15}	4.62×10^{-15}	0.923	4.04×10^{-11}	2.89×10^{-11}	5.74×10^{-14}	1.47×10^{-11}	-	1.54×10^{-12}	2.66×10^{-07}
T1D	rs12924729	16p13.13	11,187,782	1.21×10^{-08}	1.21×10^{-08}	0.027	1.05×10^{-05}	1.77×10^{-05}	1.46×10^{-07}	1.72×10^{-06}	-	2.8×10^{-05}	-
