# Supplementary material for: PUMA: A Unified Framework for Penalized Multiple Regression Analysis of GWAS Data
Source: PLoS Comput Biol. 2013 Jun 27;9(6):e1003101. doi: 10.1371/journal.pcbi.1003101 (PMC3694815; doi:10.1371/journal.pcbi.1003101)
Supplement: Table S3 — Associations recapitulated in independent studies. Regions which are significant either by single marker analysis, conditional regression, or a PMR method and which recapitulate a known association to the same disease in an independent study that does not include data from the WTCCC. The table includes all regions with a VBAY posterior probability0.97, an MCP p-value1, or a p-value for any other method 1. (PDF) [file pcbi.1003101.s025.pdf]

Table 3: Regions which are significant either by single marker analysis, conditional regression, or a PMR method and which recapitulate a known association to the same disease in an independent study that does not include data from the WTCCC. The table includes all regions with a VBAY posterior probability > 0.97, an MCP p-value $< 1 \times 10^{-7}$, or a p-value for any other method $< 1 \times 10^{-6}$.

				Method											
disease	SNP	chromosome	position	SMA	Conditional	VBAY	Lasso	Adaptive Lasso	2D-MCP	LOG	NEG	1D-MCP	perm-MCP	genes	references
CD	rs11805303	1p31.3	67,675,515	2.34×10^{-12}	2.34×10^{-12}	1	3.18×10^{-13}	9.77×10^{-15}	2.43×10^{-18}	3.23×10^{-13}	2.92×10^{-14}	1.64×10^{-13}	-	IL23R	[1, 2, 3, 4]
CD	rs6737398	2q37.1	234,170,396	1.54×10^{-14}	1.54×10^{-14}	1	1.66×10^{-13}	1.73×10^{-13}	6.69×10^{-19}	4.04×10^{-13}	9.32×10^{-13}	3.09×10^{-12}	1.19×10^{-13}	ATG16L1	[1, 4]
CD	rs4957297	5p13.1	40,455,073	5.09×10^{-14}	5.09×10^{-14}	1	1.38×10^{-12}	8.02×10^{-09}	1.13×10^{-17}	4.71×10^{-12}	7.84×10^{-15}	1.07×10^{-12}	-	Intergenic, PTGER4	[2, 5]
CD	rs274547	5q31.1	131,731,303	6.37×10^{-07}	6.37×10^{-07}	0.064	4.4×10^{-05}	2.95×10^{-05}	1.42×10^{-07}	2.58×10^{-05}	3.29×10^{-06}	2.3×10^{-05}	-	IBD5	[4]
CD	rs10995271	10q21.2	64,438,485	1.9×10^{-07}	1.9×10^{-07}	0.8	3.13×10^{-08}	2.96×10^{-06}	3.11×10^{-08}	4.07×10^{-08}	8.03×10^{-08}	3.58×10^{-08}	-	Intergenic	[1]
CD	rs3135499	16q12.1	50,766,126	1.14×10^{-14}	1.14×10^{-14}	1	1.4×10^{-09}	2.06×10^{-10}	1.56×10^{-17}	5.95×10^{-10}	1.58×10^{-09}	3.72×10^{-09}	-	NOD2	[1, 2, 5, 3, 4]
RA	rs1230649	1p13.2	114,244,176	2.16×10^{-24}	2.16×10^{-24}	1	7.64×10^{-18}	4.1×10^{-19}	1.31×10^{-24}	4.48×10^{-19}	4.11×10^{-20}	9.63×10^{-19}	-	PTPN22	[6, 7]
T1D	rs6679677	1p13.2	114,303,807	1.8×10^{-26}	1.8×10^{-26}	1	1.17×10^{-22}	7.17×10^{-23}	8.74×10^{-27}	2.04×10^{-23}	-	4.35×10^{-24}	-	PHTF1, PTPN22	[8, 9]
T1D	rs11171739	12q13.2	56,470,624	9.67×10^{-12}	9.67×10^{-12}	0.983	6.31×10^{-08}	3.18×10^{-09}	1.2×10^{-11}	5.52×10^{-08}	-	7.2×10^{-09}	-	ERBB3, RAB5B, SUOX, IKZF4, CDK	[8, 10]
T1D	rs10744777	12q24.12	112,233,017	3.34×10^{-07}	3.34×10^{-07}	0.923	5.28×10^{-01}	-	-	-	-	1.17×10^{-01}	-	C12orf30	[8]
T1D	rs12708716	16p13.13	11,179,872	1.21×10^{-08}	1.21×10^{-08}	0.027	1.05×10^{-05}	1.77×10^{-05}	1.46×10^{-07}	1.72×10^{-06}	-	2.8×10^{-05}	-	CLEC16A	[8, 9]
T1D	rs2542151	18p11.21	12,779,946	2.86×10^{-06}	2.86×10^{-06}	0.288	1.06×10^{-06}	9×10^{-07}	1.15×10^{-07}	4.89×10^{-08}	-	1.43×10^{-07}	-	PTPN2	[8]

References

[1] Rioux JD, Xavier RJ, Taylor KD, Silverberg MS, Goyette P, et al. (2007) Genome-wide association study identifies new susceptibility loci for Crohn disease and implicates autophagy in disease pathogenesis. Nature Genetics 39: 596–604.

[2] Libioulle C, Louis E, Hansoul S, Sandor C, Farnir F, et al. (2007) Novel Crohn disease locus identified by genome-wide association maps to a gene desert on 5p13.1 and modulates expression of PTGER4. PLoS Genetics 3: e58.

[3] Raelson JV, Little RD, Ruether A, Fournier H, Paquin B, et al. (2007) Genome-wide association study for Crohn's disease in the Quebec Founder Population identifies multiple validated disease loci. Proceedings of the National Academy of Sciences of the United States of America 104: 14747–14752.

[4] McGovern DPB, Jones MR, Taylor KD, Marciante K, Yan X, et al. (2010) Fucosyltransferase 2 (FUT2) non-secretor status is associated with Crohn's disease. Human Molecular Genetics 19: 3468–76.

[5] Franke A, Hampe J, Rosenstiel P, Becker C, Wagner F, et al. (2007) Systematic association mapping identifies NELL1 as a novel IBD disease gene. PLoS ONE 2: e691.

[6] Plenge RM, Seielstad M, Padyukov L, Lee AT, Remmers EF, et al. (2007) TRAF1-C5 as a risk locus for rheumatoid arthritis–a genomewide study. The New England Journal of Medicine 357: 1199–209.

[7] Gregersen PK, Amos CI, Lee AT, Lu Y, Remmers EF, et al. (2009) REL, encoding a member of the NF-kappaB family of transcription factors, is a newly defined risk locus for rheumatoid arthritis. Nature Genetics 41: 820–3.

[8] Todd Ja, Walker NM, Cooper JD, Smyth DJ, Downes K, et al. (2007) Robust associations of four new chromosome regions from genome-wide analyses of type 1 diabetes. Nature Genetics 39: 857–64.

[9] Hakonarson H, Grant SFa, Bradfield JP, Marchand L, Kim CE, et al. (2007) A genome-wide association study identifies KIAA0350 as a type 1 diabetes gene. Nature 448: 591–594.

[10] Hakonarson H, Qu Hq, Bradfield JP, Marchand L, Kim CE, et al. (2008) A novel susceptibility locus for type 1 diabetes on Chr12q13 identified by a genome-wide association study. Diabetes 57: 1143–6.
