# Supplementary material for: PUMA: A Unified Framework for Penalized Multiple Regression Analysis of GWAS Data
Source: PLoS Comput Biol. 2013 Jun 27;9(6):e1003101. doi: 10.1371/journal.pcbi.1003101 (PMC3694815; doi:10.1371/journal.pcbi.1003101)
Supplement: Table S4 — Associations recapitulated in non-independent studies. Regions which are significant either by single marker analysis, conditional regression, or a PMR method and which recapitulate a known association to the same disease in a non-independent study that includes data from the WTCCC. The table includes all regions with a VBAY posterior probability 0.97, an MCP p-value1, or a p-value for any other method 1. (PDF) [file pcbi.1003101.s026.pdf]

Table S4: Associations recapitulated in non-independent studies. Regions which are significant either by single marker analysis, conditional regression, or a PMR method and which recapitulate a known association to the same disease in a non-independent study that includes data from the WTCCC. The table includes all regions with a VBAY posterior probability > 0.97, an MCP p-value $< 1 \times 10^{-7}$, or a p-value for any other method $< 1 \times 10^{-6}$.

				Method											
disease	SNP	chromosome	position	SMA	Conditional	VBAY	Lasso	Adaptive Lasso	2D-MCP	LOG	NEG	1D-MCP	perm-MCP	genes	references
CD	rs6688532	1q24.3	172,892,951	8.35×10^{-06}	5.53×10^{-06}	0.764	1.15×10^{-05}	1.74×10^{-05}	9.26×10^{-08}	7.83×10^{-06}	1.61×10^{-07}	8.56×10^{-06}	-	Intergenic	[1, 2]
CD	rs1000113	5q33.1	150,240,075	5.99×10^{-07}	5.99×10^{-07}	0.973	3.69×10^{-05}	3.11×10^{-05}	1.49×10^{-10}	1.21×10^{-05}	6.14×10^{-07}	9.02×10^{-06}	-	IRGM	[1, 3, 2, 4]
CD	rs6908425	6p22.3	20,728,730	2.57×10^{-06}	2.57×10^{-06}	0.973	4.54×10^{-08}	1.32×10^{-08}	9.3×10^{-07}	2.69×10^{-07}	4.18×10^{-07}	3.59×10^{-08}	-	CDKAL1	[2]
CD	rs4870943	8q24.13	126,546,388	2.01×10^{-05}	2.01×10^{-05}	0.864	1.14×10^{-05}	2.24×10^{-05}	9.59×10^{-09}	7.12×10^{-06}	7.73×10^{-06}	3.78×10^{-06}	-	Intergenic	[2]
CD	rs10883365	10q24.2	101,287,763	5.55×10^{-08}	5.55×10^{-08}	0.99	3.78×10^{-06}	1.87×10^{-06}	1.03×10^{-08}	2.85×10^{-06}	1.57×10^{-06}	4.83×10^{-06}	-	NKX2-3	[1, 3, 2, 4]
CD	rs744166	17q21.2	40,514,200	1.9×10^{-05}	1.78×10^{-05}	0.0246	5.53×10^{-05}	3.58×10^{-05}	9.21×10^{-08}	9.31×10^{-05}	1.84×10^{-01}	2.97×10^{-05}	-	STAT3	[2]
CD	rs16939895	18p11.21	12,821,902	9.2×10^{-09}	9.2×10^{-09}	1	2.44×10^{-08}	8.41×10^{-09}	3.45×10^{-13}	2.32×10^{-08}	1×10^{-09}	2.32×10^{-09}	-	PTPN2	[1, 3, 2]
CD	rs2836754	21q22.2	40,291,739	5.22×10^{-05}	2.66×10^{-05}	0.0487	1.19×10^{-05}	3.31×10^{-05}	5.88×10^{-07}	7×10^{-06}	7.34×10^{-08}	9.66×10^{-07}	-	Intergenic	[1]
T1D	rs17388568	4q27	123,329,361	2.87×10^{-07}	2.87×10^{-07}	-	4.33×10^{-05}	1.54×10^{-05}	-	-	-	-	-	IL2	[5]
T1D	rs7398833	12q24.12	111,786,891	1.36×10^{-07}	1.36×10^{-07}	0.119	1.89×10^{-06}	3.34×10^{-12}	5.74×10^{-14}	7.09×10^{-07}	-	1.63×10^{-07}	-	C12orf30, SH2B3	[6, 5]
T1D	rs12150079	17q12	38,025,416	1.21×10^{-03}	2.83×10^{-05}	0.0153	1×10^{-04}	1.8×10^{-04}	6.32×10^{-10}	2.47×10^{-05}	-	1.91×10^{-04}	-	ORMDL3	[5]

References

[1] Parkes M, Barrett JC, Prescott NJ, Tremelling M, Anderson Ca, et al. (2007) Sequence variants in the autophagy gene IRGM and multiple other replicating loci contribute to Crohn's disease susceptibility. Nature Genetics 39: 830–2.

[2] Barrett JC, Hansoul S, Nicolae DL, Cho JH, Duerr RH, et al. (2008) Genome-wide association defines more than 30 distinct susceptibility loci for Crohn's disease. Nature Genetics 40: 955–62.

[3] Wellcome Trust Case Control Consortium (2007) Genome-wide association study of 14,000 cases of seven common diseases and 3,000 shared controls. Nature 447: 661–78.

[4] Franke A, McGovern DPB, Barrett JC, Wang K, Radford-Smith GL, et al. (2010) Genome-wide meta-analysis increases to 71 the number of confirmed Crohn's disease susceptibility loci. Nature Genetics 42: 1118–25.

[5] Barrett JC, Clayton DG, Concannon P, Akolkar B, Cooper JD, et al. (2009) Genome-wide association study and meta-analysis find that over 40 loci affect risk of type 1 diabetes. Nature Genetics 41: 703–707.

[6] Cooper JD, Smyth DJ, Smiles AM, Plagnol V, Walker NM, et al. (2008) Meta-analysis of genome-wide association study data identifies additional type 1 diabetes risk loci. Nature Genetics 40: 1399–1401.
