# Supplementary material for: PUMA: A Unified Framework for Penalized Multiple Regression Analysis of GWAS Data
Source: PLoS Comput Biol. 2013 Jun 27;9(6):e1003101. doi: 10.1371/journal.pcbi.1003101 (PMC3694815; doi:10.1371/journal.pcbi.1003101)
Supplement: Table S5 — Replication counts for equal numbers of hits. For each method, the number of associations in this re-analysis that replicate associations identified in a) independent (not including WTCC data) and b) non-independent (including WTCC data) datasets, where the number of markers considered as ‘hits’ is set to be equal across methods. For each method the number of hits is set to a given value and the number of replications is reported. Numbers in parentheses indicate the number of hits that are distinct from those found by the single marker analysis. (PDF) [file pcbi.1003101.s027.pdf]

Table S5: Replication counts for equal numbers of hits. For each method, the number of associations in this re-analysis that replicate associations identified in **a)** independent (not including WTCC data) and **b)** non-independent (including WTCC data) datasets, where the number of markers considered as 'hits' is set to be equal across methods. For each method the number of hits is set to a given value and the number of replications is reported. Numbers in parentheses indicate the number of hits that are distinct from those found by the single marker analysis.

a) Independent studies

	# considered as hits	Method								
		SMA	Conditional test	VBAY	Lasso	Adaptive Lasso	2D-MCP	LOG	NEG	1D-MCP
CD	5	4	4 (0)	4 (0)	4 (0)	4 (0)	4 (0)	4 (0)	4 (0)	4 (0)
	10	6	6 (0)	4 (0)	5 (0)	4 (0)	4 (0)	5 (0)	5 (0)	5 (0)
	15	6	6 (0)	4 (0)	5 (0)	5 (0)	4 (0)	5 (0)	5 (0)	5 (0)
	25	7	6 (0)	5 (0)	5 (0)	5 (0)	5 (0)	6 (0)	6 (0)	6 (0)
	30	7	6 (0)	5 (0)	5 (0)	6 (0)	6 (0)	6 (0)	6 (0)	6 (0)
RA	5	2	2 (0)	1 (0)	1 (0)	1 (0)	1 (0)	1 (0)	1 (0)	1 (0)
	10	2	2 (0)	1 (0)	1 (0)	1 (0)	1 (0)	1 (0)	1 (0)	1 (0)
	15	2	2 (0)	1 (0)	1 (0)	1 (0)	1 (0)	1 (0)	1 (0)	1 (0)
	25	2	2 (0)	1 (0)	1 (0)	1 (0)	1 (0)	1 (0)	1 (0)	1 (0)
	30	2	2 (0)	1 (0)	1 (0)	1 (0)	1 (0)	1 (0)	1 (0)	1 (0)
T1D	5	3	3 (0)	3 (0)	3 (0)	2 (0)	3 (0)	2 (0)	0	3 (0)
	10	4	4 (0)	4 (1)	4 (1)	3 (1)	3 (0)	4 (1)	0	4 (1)
	15	5	4 (0)	4 (0)	4 (0)	3 (0)	3 (0)	5 (0)	0	4 (0)
	25	5	5 (0)	5 (0)	5 (0)	4 (0)	4 (0)	5 (0)	0	5 (0)
	30	5	5 (0)	5 (0)	5 (0)	4 (0)	5 (0)	5 (0)	0	5 (0)

b) Non-independent studies

	# considered as hits	Method								
		SMA	Conditional test	VBAY	Lasso	Adaptive Lasso	2D-MCP	LOG	NEG	1-MCP
CD	5	5	5 (0)	5 (0)	5 (0)	5 (0)	5 (0)	5 (0)	5 (0)	5 (0)
	10	9	9 (0)	6 (0)	7 (1)	7 (1)	6 (0)	7 (1)	8 (2)	8 (2)
	15	11	11 (0)	8 (0)	8 (0)	8 (0)	7 (1)	8 (0)	11 (2)	11 (4)
	25	15	13 (0)	12 (2)	13 (3)	12 (2)	11 (1)	15 (3)	14 (2)	16 (4)
	30	16	14 (0)	14 (3)	14 (3)	16 (3)	12 (0)	16 (4)	16 (4)	17 (4)
RA	5	3	3 (1)	2 (1)	1 (0)	1 (0)	1 (0)	1 (0)	1 (0)	1 (0)
	10	3	4 (1)	2 (1)	2 (1)	2 (1)	1 (0)	1 (0)	2 (1)	2 (1)
	15	4	4 (1)	2 (1)	2 (0)	3 (1)	1 (0)	3 (1)	2 (0)	3 (1)
	25	6	5 (0)	3 (0)	3 (0)	3 (0)	2 (0)	3 (0)	4 (0)	3 (0)
	30	6	5 (0)	3 (0)	4 (0)	4 (0)	2 (0)	3 (0)	4 (0)	3 (0)
T1D	5	3	3 (0)	3 (0)	3 (0)	2 (0)	3 (0)	2 (0)	0	3 (0)
	10	4	4 (0)	4 (1)	4 (1)	3 (1)	4 (1)	4 (1)	0	4 (1)
	15	6	5 (0)	5 (1)	4 (0)	3 (0)	4 (1)	5 (0)	0	4 (0)
	25	7	7 (0)	7 (1)	6 (0)	7 (2)	5 (1)	6 (1)	0	5 (0)
	30	8	7 (0)	8 (1)	6 (0)	7 (2)	6 (1)	7 (2)	0	6 (1)
