# Supplementary material for: PUMA: A Unified Framework for Penalized Multiple Regression Analysis of GWAS Data
Source: PLoS Comput Biol. 2013 Jun 27;9(6):e1003101. doi: 10.1371/journal.pcbi.1003101 (PMC3694815; doi:10.1371/journal.pcbi.1003101)
Supplement: Table S6 — Additional associations for Crohn's disease. Additional associations for Crohn's disease identified by PMR methods but not a single marker analysis. (PDF) [file pcbi.1003101.s028.pdf]

**Table S6:** Additional associations for Crohn's disease identified by PMR methods but not a single marker analysis

| | | | | Method | | | | | | | | | | |
|---|---|---|---|---|---|---|---|---|---|---|---|---|---|---|
| disease | SNP | chromosome | position | SMA | Conditional | VBAY | Lasso | Adaptive Lasso | 2D-MCP | LOG | NEG | 1D-MCP | perm-MCP | genes |
| CD | rs12649928 | 4q34.3 | 181,646,381 | $2.39 \times 10^{-03}$ | $2.33 \times 10^{-03}$ | 0.034 | - | - | $9.14 \times 10^{-08}$ | - | - | - | - | |
| CD | rs338852 | 5q22.2 | 111,865,496 | $7.83 \times 10^{-04}$ | $8.12 \times 10^{-05}$ | 0.976 | $3.06 \times 10^{-06}$ | $3.24 \times 10^{-06}$ | $2.68 \times 10^{-08}$ | $2.02 \times 10^{-06}$ | - | - | - | EPB41L4A, FLJ11235, APC |
| CD | rs7820917 | 8p23.1 | 9,647,867 | $6.01 \times 10^{-05}$ | $3.84 \times 10^{-06}$ | 0.0176 | - | - | - | - | $4 \times 10^{-07}$ | - | - | TNKS, LOC157627, MSRA |
| CD | rs17663476 | 8q21.13 | 83,251,023 | $4.99 \times 10^{-05}$ | $4.15 \times 10^{-05}$ | 0.99 | $5.19 \times 10^{-06}$ | $3.63 \times 10^{-06}$ | $1 \times 10^{-08}$ | $5.03 \times 10^{-06}$ | - | $1.59 \times 10^{-05}$ | - | |
| CD | rs12548876 | 8q23.1 | 107,477,663 | $1.35 \times 10^{-03}$ | $2.22 \times 10^{-04}$ | 0.0547 | - | - | $2.87 \times 10^{-08}$ | - | - | - | - | OXR1, ABRA |
| CD | rs9783122 | 10q25.1 | 106,766,397 | $3.98 \times 10^{-04}$ | $1.55 \times 10^{-05}$ | 0.973 | $1.14 \times 10^{-05}$ | $4.12 \times 10^{-06}$ | $6.47 \times 10^{-11}$ | $3.53 \times 10^{-06}$ | $1.82 \times 10^{-08}$ | $1.21 \times 10^{-06}$ | - | SORCS3 |
| CD | rs11031006 | 11p14.1 | 30,226,527 | $4.99 \times 10^{-04}$ | $1.77 \times 10^{-05}$ | 0.987 | $1.07 \times 10^{-05}$ | $1.55 \times 10^{-05}$ | $6.32 \times 10^{-08}$ | $7.16 \times 10^{-06}$ | - | $1.32 \times 10^{-05}$ | - | KCNA4, FSHB, C11orf46, MPPED2 |
| CD | rs17224453 | 14q32.12 | 91,928,907 | $2.04 \times 10^{-03}$ | $1.84 \times 10^{-03}$ | 0.0152 | - | - | $1.08 \times 10^{-08}$ | - | - | - | - | C14orf159, GPR68, CCDC88C, SMEK1, C14orf184, CATSPERB |
| CD | rs4789523 | 17q25.3 | 76,012,853 | $2.61 \times 10^{-04}$ | $6.35 \times 10^{-05}$ | 0.936 | $6.2 \times 10^{-05}$ | $5.35 \times 10^{-05}$ | $5.24 \times 10^{-09}$ | $2.89 \times 10^{-05}$ | - | - | - | 11 genes |
| CD | rs4807569 | 19p13.3 | 1,123,377 | $6.06 \times 10^{-06}$ | $5.41 \times 10^{-06}$ | 0.975 | $8.46 \times 10^{-07}$ | $7.21 \times 10^{-07}$ | $3.67 \times 10^{-09}$ | $1.14 \times 10^{-06}$ | $8.63 \times 10^{-06}$ | $7.68 \times 10^{-07}$ | - | 29 genes |
| CD | rs1759092 | 19q13.11 | 34,676,537 | $1.06 \times 10^{-03}$ | $1.46 \times 10^{-04}$ | 0.894 | - | - | $9.31 \times 10^{-09}$ | - | - | - | - | LSM14A, KIAA0355, GPI, PDCD2L, UBA2, WTIP |
| CD | rs3126001 | Xq23 | 112,327,813 | $6.15 \times 10^{-05}$ | $6.15 \times 10^{-05}$ | 0.176 | $1.06 \times 10^{-05}$ | $4.79 \times 10^{-06}$ | $2.58 \times 10^{-09}$ | $4.77 \times 10^{-05}$ | - | - | - | AMOT |
