# Supplementary material for: PUMA: A Unified Framework for Penalized Multiple Regression Analysis of GWAS Data
Source: PLoS Comput Biol. 2013 Jun 27;9(6):e1003101. doi: 10.1371/journal.pcbi.1003101 (PMC3694815; doi:10.1371/journal.pcbi.1003101)
Supplement: Table S7 — Additional associations for rheumatoid arthritis. Additional associations for rheumatoid arthritis identified by PMR methods but not a single marker analysis. (PDF) [file pcbi.1003101.s029.pdf]

Table S7: Additional associations for rheumatoid arthritis identified by PMR methods but not a single marker analysis

				Method										
disease	SNP	chromosome	position	SMA	Conditional	VBAY	Lasso	Adaptive Lasso	2D-MCP	LOG	NEG	1D-MCP	perm-MCP	genes
RA	rs11162922	1p31.1	80,572,057	6.57×10^{-06}	6.45×10^{-06}	0.068	2.03×10^{-06}	1.36×10^{-05}	7.29×10^{-08}	1.56×10^{-06}	4.1×10^{-05}	1.65×10^{-06}	-	
RA	rs9872427	3p26.1	7,164,222	6.16×10^{-04}	6.16×10^{-04}	0.0104	1.33×10^{-05}	3.84×10^{-05}	7.99×10^{-08}	2.03×10^{-05}	-	-	-	GRM7
RA	rs702347	6q24.3	147,887,568	7.25×10^{-04}	1.07×10^{-04}	0.175	1.86×10^{-07}	1.44×10^{-07}	5.86×10^{-06}	1.58×10^{-06}	8.41×10^{-07}	1.58×10^{-06}	-	STXBP5, SAMD5
RA	rs11761231	7q32.3	131,370,038	4.55×10^{-06}	4.55×10^{-06}	0.173	1.19×10^{-05}	6.12×10^{-06}	3.13×10^{-08}	4.94×10^{-06}	1.9×10^{-05}	1.01×10^{-05}	-	MKLN1, PODXL
RA	rs4133002	8q13.3	72,718,580	1.24×10^{-04}	1.24×10^{-04}	-	-	-	9.46×10^{-09}	-	-	-	-	MSC, TRPA1
RA	rs2769190	9q33.1	120,942,581	4.89×10^{-03}	2.82×10^{-04}	-	-	-	1.89×10^{-08}	-	-	-	-	
RA	rs2514189	11q12.1	57,943,327	5.08×10^{-06}	5.08×10^{-06}	0.0837	1.85×10^{-07}	4.99×10^{-08}	1.89×10^{-12}	1.82×10^{-07}	2.44×10^{-09}	3.94×10^{-07}	-	12 genes
RA	rs11068744	12q24.23	118,364,177	5.86×10^{-04}	2.45×10^{-04}	0.0914	5.8×10^{-04}	1.92×10^{-03}	3.95×10^{-08}	6.75×10^{-05}	4.92×10^{-05}	1.24×10^{-04}	-	KSR2, RFC5, WSB2, VSIG10, PEBP1, TAOK3
RA	rs17365438	15q24.3	76,833,837	6.95×10^{-04}	1.53×10^{-05}	0.0717	5.17×10^{-07}	5.7×10^{-07}	5.96×10^{-10}	2.58×10^{-07}	3.4×10^{-07}	7.48×10^{-07}	-	ETFA, ISL2, SCAPER
RA	rs4889989	17q25.3	78,151,859	2.61×10^{-03}	8.45×10^{-04}	-	-	-	4.03×10^{-09}	-	-	-	-	10 genes
RA	rs6526752	Xp22.2	14,606,404	1.31×10^{-04}	3×10^{-05}	-	9.87×10^{-06}	5.24×10^{-06}	3.39×10^{-08}	3.47×10^{-06}	2.08×10^{-05}	-	-	GLRA2, FANCB, MOSPD2
