# Supplementary material for: PUMA: A Unified Framework for Penalized Multiple Regression Analysis of GWAS Data
Source: PLoS Comput Biol. 2013 Jun 27;9(6):e1003101. doi: 10.1371/journal.pcbi.1003101 (PMC3694815; doi:10.1371/journal.pcbi.1003101)
Supplement: Table S8 — Additional associations for type 1 diabetes. Additional associations for type 1 diabetes identified by PMR methods but not a single marker analysis. (PDF) [file pcbi.1003101.s030.pdf]

Table S8: Additional associations for type 1 diabetes identified by PMR methods but not a single marker analysis

				Method										
disease	SNP	chromosome	position	SMA	Conditional	VBAY	Lasso	Adaptive Lasso	2D-MCP	LOG	NEG	1D-MCP	perm-MCP	genes
T1D	rs577193	1q43	236,802,015	2.32×10^{-04}	2.32×10^{-04}	0.0483	2.9×10^{-04}	3.29×10^{-04}	6.09×10^{-08}	1.09×10^{-04}	-	-	-	EDARADD, LGALS8, HEATR1, ACTN2, MTR
T1D	rs10865679	3p13	73,781,216	1.96×10^{-02}	2.2×10^{-03}	-	-	-	5.75×10^{-08}	-	-	-	-	PDZRN3
T1D	rs4707786	6q13	71,058,226	5.11×10^{-04}	1.75×10^{-04}	0.375	1.14×10^{-05}	8.68×10^{-06}	3.32×10^{-08}	1.78×10^{-05}	-	3.5×10^{-05}	-	COL19A1, COL9A1, FAM135A, C6orf57
T1D	rs760500	6q27	170,725,386	5.24×10^{-04}	1.66×10^{-05}	0.133	1.55×10^{-07}	2.04×10^{-07}	6.63×10^{-10}	5.32×10^{-08}	-	1.77×10^{-07}	-	LOC154449, DLL1, FAM120B, PSMB1, TBP, PDCD2
T1D	rs346613	8q13.3	73,242,672	2.6×10^{-03}	2×10^{-05}	0.152	5.5×10^{-06}	3.22×10^{-06}	5.33×10^{-08}	2.32×10^{-06}	-	3.79×10^{-06}	-	TRPA1, KCNB2
T1D	rs10759987	9q33.1	121,364,133	9.98×10^{-05}	9.98×10^{-05}	0.0377	4.61×10^{-05}	5.62×10^{-05}	4.58×10^{-09}	5.07×10^{-05}	-	7.32×10^{-05}	-	
T1D	rs2666236	10p11.22	33,418,871	4.46×10^{-05}	2.24×10^{-06}	0.0374	1.12×10^{-04}	9.66×10^{-05}	3.88×10^{-08}	3.08×10^{-05}	-	1.04×10^{-04}	-	C10orf68, ITGB1, NRP1
T1D	rs4938390	11q23.3	117,358,312	9.24×10^{-05}	3.08×10^{-05}	0.0356	2.21×10^{-06}	7.65×10^{-07}	8.48×10^{-06}	4.78×10^{-07}	-	8.86×10^{-07}	-	7 genes
T1D	rs17756934	12q23.3	107,949,264	2.34×10^{-02}	1.65×10^{-04}	0.0127	3.27×10^{-06}	8.64×10^{-06}	7.99×10^{-12}	8×10^{-06}	-	3.76×10^{-06}	-	BTBD11, PWP1, PRDM4, ASCL4
T1D	rs341557	13q21.2	60,367,013	4.45×10^{-05}	4.45×10^{-05}	-	-	-	2.58×10^{-09}	2×10^{-05}	-	-	-	DIAPH3
T1D	rs1357809	18q12.1	27,656,198	1.8×10^{-02}	5.1×10^{-04}	0.011	-	-	6.04×10^{-08}	6.69×10^{-05}	-	-	-	
