# Supplementary material for: PUMA: A Unified Framework for Penalized Multiple Regression Analysis of GWAS Data
Source: PLoS Comput Biol. 2013 Jun 27;9(6):e1003101. doi: 10.1371/journal.pcbi.1003101 (PMC3694815; doi:10.1371/journal.pcbi.1003101)
Supplement: Text S1 — Parameters for running HyperLasso. (PDF) [file pcbi.1003101.s031.pdf]

Supplementary Text S1

PUMA: A Unified Framework for Penalized Multiple Regression Analysis of GWAS Data

Gabriel E. Hoffman, Benjamin A. Logsdon, Jason G. Mezey

We ran hyperLasso following the instructions on the program website (`http://www.ebi.ac.uk/projects/BARGEN/`) that suggested the following flags:

```
-shape 0.1 -lambda 50 -std
```

Setting the lambda values based on asymptotic arguments described by Hoggart et al. [20] either produced very few markers with non-zero coefficients or caused the program to crash.
