# Supplementary material for: PUMA: A Unified Framework for Penalized Multiple Regression Analysis of GWAS Data
Source: PLoS Comput Biol. 2013 Jun 27;9(6):e1003101. doi: 10.1371/journal.pcbi.1003101 (PMC3694815; doi:10.1371/journal.pcbi.1003101)
Supplement: Text S2 — Efficient coordinate-wise gradient descent algorithms for high-dimensional penalized generalized linear models with convex or nonconvex penalties. (PDF) [file pcbi.1003101.s032.pdf]

Supplementary Text S2

PUMA: A Unified Framework for Penalized Multiple Regression Analysis of GWAS Data

Gabriel E. Hoffman, Benjamin A. Logsdon, Jason G. Mezey

Efficient coordinate-wise gradient descent algorithms for high-dimensional penalized generalized linear models with convex or nonconvex penalties.

Here we derive the algorithms used by PUMA for efficient estimation in penalized generalized linear models (GLM). In order to make the derivations general so that they are applicable to GLM's with normal or binary responses, we use the following standard notation to describe GLM's [1].

$\mathbf{X}$	matrix of features
$\boldsymbol{\beta}$	vector of regression coefficients
$\mathbf{y}$	vector of observed responses
$\boldsymbol{\eta}^{(s)}$	value of linear predictor at s^{th} iteration
$\ell(\boldsymbol{\eta})$	log-likelihood function of the data based on $\boldsymbol{\eta}$
$\ell(\boldsymbol{\beta})$	reparameterized log-likelihood based on $\boldsymbol{\beta}$
$\boldsymbol{\mu}$	estimated response on the same scale at the observed response, $\mathbf{y}$
$V(\boldsymbol{\mu})$	function of estimated response that is proportional to $var(\mathbf{y})$
$g(.)$	link funtion for a GLM so that $g^{-1}(\boldsymbol{\eta}) = \boldsymbol{\mu}$
θ	cannonical parameter of the GLM
$b(\theta)$	defined so that likelihood is a member of the exponential family
$\mathbf{v}$	$\frac{\partial \ell(\boldsymbol{\eta})}{\partial \boldsymbol{\eta}}$
$\mathbf{W}$	$-\frac{\partial^2 \ell(\boldsymbol{\eta})}{\partial \boldsymbol{\eta} \partial \boldsymbol{\eta}^T}$

1 Log-likelihood of a generalized linear model and its derivatives

Consider the log-likelihood of a GLM as a function of $\boldsymbol{\eta}$ and obtain the first derivative using

$$\mu_i = b'(\theta_i) = g^{-1}(\boldsymbol{\eta}_i) \tag{1}$$

and

$$g(\mu_i) = \boldsymbol{\eta}_i = \mathbf{X}_i \boldsymbol{\beta} \tag{2}$$

where vectors are indexed by i [1]. Since the response comes from a distribution in the exponential family, the log-likelihood in cannonical form satisfies

$$\ell(\boldsymbol{\eta}) \quad \propto \quad \sum_{i=1}^{n} [y_i \theta_i - b(\theta_i)] \tag{3}$$

The derivative of $\ell(\boldsymbol{\eta})$ follows from the standard properties of a GLM [1]:

$$\begin{aligned}
\frac{\partial \ell}{\partial \eta_i} &= [y_i - b'(\theta_i)]\frac{\partial \theta_i}{\partial \eta_i} &(4)\\
&= [y_i - b'(\theta_i)]\frac{\partial \theta_i}{\partial \mu_i}\frac{\partial \mu_i}{\partial \eta_i} &(5)\\
&= [y_i - b'(\theta_i)]\frac{1}{b''(\theta_i)}\frac{1}{g'(\mu_i)} &(6)\\
&= [y_i - b'(\theta_i)]\frac{1}{V(\mu_i)g'(\mu_i)} &(7)\\
&= \frac{(y_i - \mu_i)}{V(\mu_i)g'(\mu_i)} &(8)
\end{aligned}$$

Using the fact that $V(\mu_i)g'(\mu_i) = 1$ if the cannonical link is used and expressing the derivative in matrix notation:

$$\begin{aligned}
\mathbf{v}_{n\times 1} &= \frac{\partial \ell}{\partial \boldsymbol{\eta}} &(9)\\
&= \mathbf{y} - \boldsymbol{\mu} &(10)
\end{aligned}$$

The second derivative is:

$$\begin{aligned}
\frac{\partial^2 \ell}{\partial \eta_i \partial \eta_{i'}} &= \frac{\partial \mu_i}{\partial \eta_{i\prime}} &(11)\\
&= \frac{1}{g'(\mu_{i\prime})} &(12)
\end{aligned}$$

Let $\mathbf{W}$ denote the negative Hessian and notice that the off-diagonal elements are zero:

$$\begin{aligned}
\mathbf{W}_{n\times n} &= -\frac{\partial \ell}{\partial \boldsymbol{\eta} \partial \boldsymbol{\eta}^T} &(13)\\
&= \mathrm{diag}\left[\frac{1}{g'(\boldsymbol{\mu})}\right] &(14)
\end{aligned}$$

If the cannonical link is used, the formula for $\mathbf{W}$ is:

Table 1: Formulas for $\mathbf{W}$

response	$g(\mu_i)$	$g'(\mu_i)$	$\mathbf{W}$
normal	1	1	diag(1)
binomial	$\log \frac{\mu_i}{1-\mu_i}$	$\frac{1}{\mu_i(1-\mu_i)}$	$\mathrm{diag}\,[\boldsymbol{\mu}(1-\boldsymbol{\mu})]$

2 Quadratic approximation to the log-likelihood

The standard iteritively reweighted least squares (IRLS) algorithm follows from a quadratic approximation of the log-likelihood. We derive this approximation here and derive the iterative algorithm

in the next section. Based on the second order Taylor expansion of the log-likelihood,

$$\begin{aligned}
\ell(\boldsymbol{\eta}) &\approx \ell(\boldsymbol{\eta}^{(s)}) + (\boldsymbol{\eta} - \boldsymbol{\eta}^{(s)})^T \ell'(\boldsymbol{\eta}^{(s)}) + \frac{1}{2}(\boldsymbol{\eta} - \boldsymbol{\eta}^{(s)})\ell''(\boldsymbol{\eta}^{(s)})(\boldsymbol{\eta} - \boldsymbol{\eta}^{(s)}) && (15)\\
&= (\boldsymbol{\eta} - \boldsymbol{\eta}^{(s)})^T \mathbf{v} - \frac{1}{2}(\boldsymbol{\eta} - \boldsymbol{\eta}^{(s)})^T W (\boldsymbol{\eta} - \boldsymbol{\eta}^{(s)}) && (16)\\
&= -\frac{1}{2}(\boldsymbol{\eta} - \boldsymbol{\eta}^{(s)} - \mathbf{v}\mathbf{W}^{-1})^T \mathbf{W} (\boldsymbol{\eta} - \boldsymbol{\eta}^{(s)} - \mathbf{v}\mathbf{W}^{-1}) && (17)\\
&= -\frac{1}{2}(\tilde{\mathbf{y}} - \boldsymbol{\eta})^T \mathbf{W} (\tilde{\mathbf{y}} - \boldsymbol{\eta}) && (18)
\end{aligned}$$

where the working response, $\tilde{\mathbf{y}} = \boldsymbol{\eta}^{(s)} + \mathbf{v}\mathbf{W}^{-1}$, is a function of the linear predictor at the sth iteration. Reparameterizing as a function of $\boldsymbol{\beta}$,

$$\ell(\boldsymbol{\beta}) = -\frac{1}{2}(\tilde{\mathbf{y}} - \mathbf{X}\boldsymbol{\beta})^T \mathbf{W} (\tilde{\mathbf{y}} - \mathbf{X}\boldsymbol{\beta}) \qquad (19)$$

This is equivalent to the IRLS Fisher scoring system where the Hessian is replaced by its expectation [1]. In standard Fisher scoring notation, the expected Hessian is:

$$E\left[\frac{\partial^2 \ell}{\partial \boldsymbol{\beta} \partial \boldsymbol{\beta}}\right] = -\mathbf{X}^T \mathbf{W} \mathbf{X} \qquad (20)$$

as is the case in equation (19). Expressed in stardard notation, the working responses are

$$\tilde{\mathbf{y}} = g(\boldsymbol{\mu}) + g'(\boldsymbol{\mu})(\mathbf{y} - \boldsymbol{\mu}) \qquad (21)$$

where it is trivial to show this is equivalent to $\tilde{\mathbf{y}} = \boldsymbol{\eta}^{(s)} + \mathbf{v}\mathbf{W}^{-1}$.

2.1 Details of derivation

Equation numbers and an explanation of their derivation:

(15) A second order approximation to $\ell(\boldsymbol{\eta})$[2]. This is equivalent to the Fisher scoring algorithm of IRLS.

(16) Plug in $\mathbf{v}$ for the first derivative and $-\mathbf{W}$ for the Hessian and drop terms that don't depend on $\boldsymbol{\eta}$ (i.e. drop $\ell(\boldsymbol{\eta}^{(s)})$)

(17) Complete the square [2]: In general, $ax^2 + bx + c = a(x-h)^2 + k$ where $h = -\frac{b}{2a}$ and $k = c - \frac{b^2}{4a}$ In this case, $a = -\frac{1}{2}\mathbf{W}$, $b = \mathbf{v}$, $c = 0$ and $x = (\mathbf{X}\boldsymbol{\beta} - \boldsymbol{\eta}^{(s)})$ so $h = \mathbf{v}\mathbf{W}^{-1}$ and k can be dropped since it does not depend on $\boldsymbol{\beta}$.

(18) Plug in $\tilde{\mathbf{y}} = \boldsymbol{\eta}^{(s)} + \mathbf{v}\mathbf{W}^{-1}$

3 Estimation in unpenalized generalized linear models

While standard IRLS methods update all regression parameters in a single step, here we derive a coordinate-wise gradient descent algorithm for estimating $\boldsymbol{\beta}$ in an unpenalized GLM and extend it

to the penalized case in the next section. Following [3, 4, 5], consider the quadratic approximation to the log-likelihood and construct a coordinate-wise Newton-Raphson update of β_j using the first and second derivatives.

$$\begin{aligned}
\ell(\boldsymbol{\beta}) &= -\frac{1}{2}(\tilde{\mathbf{y}} - \mathbf{X}\boldsymbol{\beta})^T \mathbf{W}(\tilde{\mathbf{y}} - \mathbf{X}\boldsymbol{\beta}) & (22)\\
&= -\frac{1}{2}(\tilde{\mathbf{y}} - \sum_{k \neq j} x_k \beta_k - x_j \beta_j)^T \mathbf{W}(\tilde{\mathbf{y}} - \sum_{k \neq j} \mathbf{x}_k \beta_k - \mathbf{x}_j \beta_j) & (23)\\
\frac{\partial \ell}{\partial \beta_j} &= (\tilde{\mathbf{y}} - \sum_{k \neq j} \mathbf{x}_k \beta_k - \mathbf{x}_j \beta_j)^T \mathbf{W}\mathbf{x}_j & (24)\\
&= (\tilde{\mathbf{y}} - \sum_{k \neq j} \mathbf{x}_k \beta_k)^T \mathbf{W}\mathbf{x}_j - \beta_j \mathbf{x}_j^T \mathbf{W}\mathbf{x}_j & (25)\\
&= (\mathbf{x}_j \beta_j^{(s)} + \mathbf{v})^T \mathbf{W}\mathbf{x}_j - \beta_j \mathbf{x}_j^T \mathbf{W}\mathbf{x}_j & (26)\\
&= \beta_j^{(s)} \mathbf{x}_j^T \mathbf{W}\mathbf{x}_j + \mathbf{v}^T \mathbf{W}\mathbf{x}_j - \beta_j \mathbf{x}_j^T \mathbf{W}\mathbf{x}_j & (27)\\
\frac{\partial^2 \ell}{\partial \beta_j \partial \beta_j} &= -\mathbf{x}_j^T \mathbf{W}\mathbf{x}_j & (28)
\end{aligned}$$

The coordinate-wise update follows from the standard Newton-Raphson update formula.

$$\begin{aligned}
\beta_j^{(s+1)} &= \beta_j^{(s)} - \frac{\ell'(\beta_j^{(s)})}{\ell''(\beta_j^{(s)})} & (29)\\
&= \beta_j^{(s)} + \frac{\beta_j^{(s)} \mathbf{x}_j^T \mathbf{W}\mathbf{x}_j + \mathbf{v}^T \mathbf{W}\mathbf{x}_j - \beta_j^{(s)} \mathbf{x}_j^T \mathbf{W}\mathbf{x}_j}{\mathbf{x}_j^T \mathbf{W}\mathbf{x}_j} & (30)\\
&= \beta_j^{(s)} + \frac{\mathbf{v}^T \mathbf{W}\mathbf{x}_j}{\mathbf{x}_j^T \mathbf{W}\mathbf{x}_j} & (31)
\end{aligned}$$

This gives the coordinate-wise update:

$$\boxed{\beta_j^{(s+1)} = \frac{\beta_j^{(s)} \mathbf{x}_j^T \mathbf{W}\mathbf{x}_j + \mathbf{v}^T \mathbf{W}\mathbf{x}_j}{\mathbf{x}_j^T \mathbf{W}\mathbf{x}_j}} \tag{32}$$

4 Estimation in penalized generalized linear models

Following the derivation from the previous section, now consider a penalized GLM where a penalty term is added to the standard log-likelihood of a GLM. Specifically, consider a GLM log-likelihood with an arbitrary penalty $p(.)$ on the magnitute of β_j:

$$\ell(\boldsymbol{\beta}) = -\frac{1}{2}(\tilde{\mathbf{y}} - \mathbf{X}\beta)^T \mathbf{W}(\tilde{\mathbf{y}} - \mathbf{X}\beta) - \sum_j p(\beta_j) \tag{33}$$

The coordinate-wise Newton-Raphson update is:

$$\beta_j^{(s+1)} = \beta_j^{(s)} - \frac{\ell'(\beta_j^{(s)}) - p'(\beta_j^{(s)})}{\ell''(\beta_j^{(s)}) - p''(\beta_j^{(s)})} \quad (34)$$

$$= \beta_j^{(s)} + \frac{\mathbf{v}^T\mathbf{W}\mathbf{x}_j - p'(\beta_j^{(s)})}{\mathbf{x}_j^T\mathbf{W}\mathbf{x}_j + p''(\beta_j^{(s)})} \quad (35)$$

While this update considers $p(.)$ in general, specific penalty functions are considered below.

4.1 LASSO

Proposed by [6]. Consider the penalty function and its derivatives.

$$p_{\text{LASSO}}(\beta_j) = \lambda|\beta_j| \quad (36)$$

$$p'_{\text{LASSO}}(\beta_j) = \lambda sign(\beta_j) \quad (37)$$

$$p''_{\text{LASSO}}(\beta_j) = 0 \quad (38)$$

The coordinate-wise Newton-Raphson update is

$$\beta_j^{(s+1)} = \beta_j^{(s)} + \frac{\mathbf{v}^T\mathbf{W}\mathbf{x}_j - \lambda sign(\beta_j^{(s)})}{\mathbf{x}_j^T\mathbf{W}\mathbf{x}_j} \quad (39)$$

$$= \frac{\beta_j^{(s)}\mathbf{x}_j^T\mathbf{W}\mathbf{x}_j + \mathbf{v}^T\mathbf{W}\mathbf{x}_j - \lambda sign(\beta_j^{(s)})}{\mathbf{x}_j^T\mathbf{W}\mathbf{x}_j} \quad (40)$$

$$= \frac{d - \lambda sign(d)}{\mathbf{x}_j^T\mathbf{W}\mathbf{x}_j} \quad (41)$$

where $d = \beta_j^{(s)}\mathbf{x}_j^T\mathbf{W}\mathbf{x}_j + \mathbf{v}^T\mathbf{W}\mathbf{x}_j$.

Thus $|d| > \lambda$ yields $(|d| - \lambda)sign(d)$ but $|d| \leq \lambda$ yields an update of 0 so that $\beta_j^{(s+1)}$ is set to zero if the derivative of the lasso penalty exceeds the unpenalized update. This can be stated using the soft-threasholding function of [7] (see [6] for orginal use in lasso regression):

$$\boxed{\beta_j = \frac{S(d,\lambda)}{\mathbf{x}_j^T\mathbf{W}\mathbf{x}_j}} \quad (42)$$

where

$$S(d,\lambda) = \begin{cases} d-\lambda & d>0 \text{ and } \lambda<|d| \\ d+\lambda & d<0 \text{ and } \lambda<|d| \\ 0 & \lambda \geq |d| \end{cases} \quad (43)$$

Note the similar application of the soft-threasholding function by [8, 9, 5, 4, 10]. Also note that $\ell(\boldsymbol{\beta})$ is not differentiable when $\beta_j = 0$, but a directional derivative still exists and has magnitude λ [8]. Therefore the updates above are valid even if $\beta_j = 0$.

4.2 MCP

Proposed by [11]. Consider the penalty function and its derivatives.

$$p_{\text{MCP}}(\beta_j) = \begin{cases} \lambda|\beta_j| - \frac{\beta_j^2}{2a} & |\beta_j| \leq a\lambda \\ \frac{1}{2}a\lambda^2 & |\beta_j| > a\lambda \end{cases} \tag{44}$$

$$p'_{\text{MCP}}(\beta_j) = \begin{cases} \lambda sign(\beta_j^{(s)}) - \frac{\beta_j}{a} & |\beta_j| \leq a\lambda \\ 0 & |\beta_j| > a\lambda \end{cases} \tag{45}$$

$$p''_{\text{MCP}}(\beta_j) = \begin{cases} -\frac{1}{a} & |\beta_j| \leq a\lambda \\ 0 & |\beta_j| > a\lambda \end{cases} \tag{46}$$

The coordinate-wise Newton-Raphson update is

$$\beta_j^{(s+1)} = \beta_j^{(s)} + \frac{\mathbf{v}^T\mathbf{W}\mathbf{x}_j - \lambda sign(\beta_j^{(s)}) + \frac{\beta_j^{(s)}}{a}}{\mathbf{x}_j^T W\mathbf{x}_j - \frac{1}{a}} \tag{47}$$

$$= \frac{\beta_j^{(s)}(\mathbf{x}_j^T\mathbf{W}\mathbf{x}_j - \frac{1}{a}) + \mathbf{v}^T\mathbf{W}\mathbf{x}_j - \lambda sign(\beta_j^{(s)}) + \frac{\beta_j^{(s)}}{a}}{\mathbf{x}_j^T\mathbf{W}\mathbf{x}_j - \frac{1}{a}} \tag{48}$$

$$= \frac{S(d,\lambda)}{\mathbf{x}_j^T\mathbf{W}\mathbf{x}_j - \frac{1}{a}} \tag{49}$$

$$\boxed{\beta_j^{(s+1)} = \begin{cases} \frac{S(d,\lambda)}{\mathbf{x}_j^T\mathbf{W}\mathbf{x}_j - \frac{1}{a}} & d \leq a\lambda\mathbf{x}_j^T\mathbf{W}\mathbf{x}_j \\ \frac{d}{\mathbf{x}_j^T\mathbf{W}\mathbf{x}_j} & d > a\lambda\mathbf{x}_j^T\mathbf{W}\mathbf{x}_j \end{cases}} \tag{50}$$

Notice the bounds change in (50) to adjust for the scale of the second derivative according to [3, 2].

4.3 NEG

Proposed by [12, 13], but see [14] for more details. Consider the penalty function and its derivatives.

$$p_{\text{NEG}}(\beta) = -\frac{\beta^2}{4\gamma^2} - \log D_{-2\lambda-1}(\frac{|\beta|}{\gamma}) \tag{51}$$

$$p'_{\text{NEG}}(\beta) = sign(\beta)\frac{2\lambda+1}{\gamma}\frac{D_{-2\lambda-2}(\frac{|\beta|}{\gamma})}{D_{-2\lambda-1}(\frac{|\beta|}{\gamma})} \tag{52}$$

$$p''_{\text{NEG}}(\beta_j) = -\frac{4}{\gamma}\left[(\lambda+1)(\lambda+\frac{1}{2})\frac{D_{-2\lambda-3}(\frac{|\beta|}{\gamma})}{D_{-2\lambda-1}(\frac{|\beta|}{\gamma})} - \left[(\lambda+\frac{1}{2})\frac{D_{-2\lambda-2}(\frac{|\beta|}{\gamma})}{D_{-2\lambda-1}(\frac{|\beta|}{\gamma})}\right]^2\right] \tag{53}$$

$D_{\cdot}(.)$ denotes the parabolic cylinder function [15]. We use a Fortran implentation of this function available in the SciPy library: `http://projects.scipy.org/scipy/export/6949/trunk/scipy/`

special/specfun/specfun.f

The coordinate-wise Newton-Raphson update is

$$\boxed{\beta_j = \beta_j^{(s)} + \frac{\mathbf{v}^T\mathbf{W}\mathbf{x}_j - p'_{\text{NEG}}(\beta_j^{(s)})}{\mathbf{x}_j^T\mathbf{W}\mathbf{x}_j - p''_{\text{NEG}}(\beta_j^{(s)})}} \tag{54}$$

and cannot be simplified due to the complicated form for the penalty.

4.4 LOG penalty

Proposed by [16]. Consider the penalty function and its derivatives.

$$p_{\text{LOG}}(\beta_j) = \lambda \frac{\log(1 + |\beta_j|/\epsilon)}{\log(1 + 1/\epsilon)} \tag{55}$$

$$p'_{\text{LOG}}(\beta_j) = \frac{\lambda}{\log(1 + 1/\epsilon)} \frac{sign(\beta_j)/\epsilon}{1 + |\beta_j|/\epsilon} \tag{56}$$

$$p''_{\text{LOG}}(\beta_j) = \frac{-\lambda}{\log(1 + 1/\epsilon)} \frac{[sign(\beta_j)/\epsilon]^2}{[1 + |\beta_j|/\epsilon]^2} \tag{57}$$

The coordinate-wise Newton-Raphson update is

$$\boxed{\beta_j = \beta_j^{(s)} + \frac{\mathbf{v}^T\mathbf{W}\mathbf{x}_j - p'_{\text{LOG}}(\beta_j^{(s)})}{\mathbf{x}_j^T\mathbf{W}\mathbf{x}_j - p''_{\text{LOG}}(\beta_j^{(s)})}} \tag{58}$$

5 Updating quantities during iteration of algorithm

When β_j is updated, $\mathbf{v}$ and $\mathbf{W}$ must be updated accordingly. In general, initialize $\boldsymbol{\eta}$ as:

$$\boldsymbol{\eta}^{(0)} = \mathbf{X}\boldsymbol{\beta}^{(0)}. \tag{59}$$

At each iteration where $\beta_j^{(s+1)} \neq \beta_j^{(s)}$, update $\boldsymbol{\eta}$, $\mathbf{v}$ and $\mathbf{W}$ according to

$$\boldsymbol{\eta}^{(s+1)} = \boldsymbol{\eta}^{(s)} + \mathbf{x}_j \left(\beta_j^{(s+1)} - \beta_j^{(s)}\right) \tag{60}$$

$$\boldsymbol{\mu} = g^{-1}(\eta) \tag{61}$$

$$\mathbf{v} = \mathbf{y} - \boldsymbol{\mu} \tag{62}$$

$$\mathbf{W} = 1/g'(\boldsymbol{\mu}) \tag{63}$$

6 Convergence and a minorize-maximization algorithm

It is widely known that Newton-Raphson algorithms are not guaranteed to converge unless the objective function is quadratic. Since we are using a quadratic approximation of the objective function, the Fisher scoring system is not guaranteed to converge. The Fisher scoring method of IRLS tends to converge in practice in overdetermined systems, yet the highly underdetermined systems addressed here may be problematic. In fact, [14] and [17] replace the Hessian with an upper bound so that the system always converges. Intuitively, this upper bound prevents taking steps that are too large and actually decrease the value of the objective function.

These examples are generalized by [18], who describe a very general method to construct a surrogate function that minorizes the objective function. They demonstrate that iteratively maximizing the surrogate and constructing a new surrogate can guarantee convergence on non-quadratic surfaces without expensive backtracking checks that evaluate the log-likelihood at each update. One simple method to construct a surrogate function which minorizes the objective function is to replace the Hessian with an upper bound. This effectively decreases the step size so that the value of the objective function always increases and a backtracking step is not needed.

Convergence can therefore be guaranteed if the matrix $\mathbf{W}$ used in the quadratic approximation is replaced by an upper bound. Consider the upper bound on the Hessian for the following links:

- linear: the system is exactly quadratic so that $\mathbf{W} = \text{diag}(1)$ and no bound is needed.
- logistic: $\mathbf{W} = \text{diag}(\boldsymbol{\mu}(1-\boldsymbol{\mu}))$ so that $\mathbf{W}_{upper} = \frac{1}{4}\text{diag}(1)$
  since $\mu_i \in [0, 1]$ and $\max(\mu_i(1-\mu_i)) = .25$
  (see example in [18])

For logistic regression, replacing $\mathbf{W}$ with $\mathbf{W}_{upper}$ to guarantees convergence and increases the speed of each update since it avoids calculating $\boldsymbol{\mu}$ and $\boldsymbol{\mu}(1-\boldsymbol{\mu})$.

7 Implementation

Our implementation of these penalized likelihood methods is especially efficient since we 1) store the dataset to mimimize access time for each feature, 2) use highly optimized Intel® Math Kernel Library® for linear algebra operations and 3) evaluate multiple modes of the nonconvex posterior surface in parallel using OpenMP®. This very fast implementation allowed efficient exploration of the two-dimensional space of tuning parameters for MCP, LOG and NEG penalties using the relaxation method of [16].

References

[1] McCullagh P, Nelder JA (1989) Generalized Linear Models. Chapman & Hall / CRC, 2 edition.

[2] Breheny P, Huang J (2009) Penalized Methods for Bi-level variable selection. Statistics and Its Interface 2: 369–380.

[3] Breheny P, Huang J (2011) Coordinate descent algorithms for nonconvex penalized regression, with applications to biological feature selection. The Annals of Applied Statistics 5: 232–253.

[4] Friedman J, Hastie T, Tibshirani R (2010) Regularization paths for generalized linear models via coordinate descent. Journal of Statistical Software 33.

[5] Friedman J, Hastie T, Höfling H, Tibshirani R (2007) Pathwise coordinate optimization. Annals of Applied Statistics 1: 302–332.

[6] Tibshirani R (1996) Regression shrinkage and selection via the lasso. Journal of the Royal Statistical Society Series B (Methodological) 58: 267–288.

[7] Donoho DL, Johnstone JM (1994) Ideal spatial adaptation by wavelet shrinkage. Biometrika 81: 425–455.

[8] Wu TT, Lange K (2008) Coordinate descent algorithms for lasso penalized regression. Annals of Applied Statistics 2: 224–244.

[9] Wu TT, Chen YF, Hastie T, Sobel E, Lange K (2009) Genome-wide association analysis by lasso penalized logistic regression. Bioinformatics 25: 714–21.

[10] Schifano ED, Strawderman RL, Wells MT (2010) Majorization-Minimization algorithms for nonsmoothly penalized objective functions. Electronic Journal of Statistics 4: 1258–1299.

[11] Zhang CH (2010) Nearly unbiased variable selection under minimax concave penalty. The Annals of Statistics 38: 894–942.

[12] Griffin J, Brown P (2005) Alternative prior distributions for variable selection with very many more variables than observations. Technical Report University of Kent .

[13] Griffin JE, Brown PJ (2007) Bayesian adaptive lassos with non-convex penalization. Technical Report University of Warwick .

[14] Hoggart CJ, Whittaker JC, De Iorio M, Balding DJ, Iorio MD (2008) Simultaneous analysis of all SNPs in genome-wide and re-sequencing association studies. PLoS Genetics 4: e1000130.

[15] Oldham K, Myland J, Spanier J (2008) An atlas of functions: with Equator, the atlas function calculator. Springer Verlag.

[16] Mazumder R (2011) SparseNet : Coordinate Descent With Nonconvex Penalties. Journal of the American Statistical Association : 1–14.

[17] Genkin A, Lewis DD, Madigan D (2007) Large-Scale Bayesian Logistic Regression for Text Categorization. Technometrics 49: 291–304.

[18] Hunter DR, Lange K (2004) A Tutorial on MM Algorithms. The American Statistician 58: 30–37.
